# Generalising electrocardiogram detection and delineation: training convolutional neural networks with synthetic data augmentation

**DOI:** 10.3389/fcvm.2024.1341786

**Published:** 2024-07-19

**Authors:** Guillermo Jimenez-Perez, Juan Acosta, Alejandro Alcaine, Oscar Camara

**Affiliations:** ^1^ Department of Information and Communication Technologies, PhySense Research Group, BCN-MedTech, Barcelona, Spain; ^2^ Arrhythmia Unit, Department of Cardiology, Virgen Del Rocío University Hospital, Seville, Spain; ^3^ Institut d’Investigacions Biomèdiques August Pi i Sunyer, Barcelona, Spain; ^4^ Computing for Medical and Biological Applications (CoMBA) Group, Facultad de Ciencias de la Salud, Universidad San Jorge, Zaragoza, Spain

**Keywords:** digital health, electrocardiogram, convolutional neural network, artificial intelligence, delineation, multi-centre study, data augmentation, segmentation

## Abstract

**Introduction:**

Extracting beat-by-beat information from electrocardiograms (ECGs) is crucial for various downstream diagnostic tasks that rely on ECG-based measurements. However, these measurements can be expensive and time-consuming to produce, especially for long-term recordings. Traditional ECG detection and delineation methods, relying on classical signal processing algorithms such as those based on wavelet transforms, produce high-quality delineations but struggle to generalise to diverse ECG patterns. Machine learning (ML) techniques based on deep learning algorithms have emerged as promising alternatives, capable of achieving similar performance without handcrafted features or thresholds. However, supervised ML techniques require large annotated datasets for training, and existing datasets for ECG detection/delineation are limited in size and the range of pathological conditions they represent.

**Methods:**

This article addresses this challenge by introducing two key innovations. First, we develop a synthetic data generation scheme that probabilistically constructs unseen ECG traces from “pools” of fundamental segments extracted from existing databases. A set of rules guides the arrangement of these segments into coherent synthetic traces, while expert domain knowledge ensures the realism of the generated traces, increasing the input variability for training the model. Second, we propose two novel segmentation-based loss functions that encourage the accurate prediction of the number of independent ECG structures and promote tighter segmentation boundaries by focusing on a reduced number of samples.

**Results:**

The proposed approach achieves remarkable performance, with a $F_{1}$-score of 99.38% and delineation errors of 2.19±17.73 ms and 4.45±18.32 ms for ECG segment onsets and offsets across the P, QRS, and T waves. These results, aggregated from three diverse freely available databases (QT, LU, and Zhejiang), surpass current state-of-the-art detection and delineation approaches.

**Discussion:**

Notably, the model demonstrated exceptional performance despite variations in lead configurations, sampling frequencies, and represented pathophysiology mechanisms, underscoring its robust generalisation capabilities. Real-world examples, featuring clinical data with various pathologies, illustrate the potential of our approach to streamline ECG analysis across different medical settings, fostered by releasing the codes as open source.

## 1 Introduction

The electrocardiogram (ECG) stands as a fundamental tool in clinical practice, offering valuable insights into cardiac electrophysiology. The ECG captures the heart’s electrical activity, presenting it as distinct waves corresponding to different phases in the cardiac cycle. Specifically, the P wave signifies atrial depolarisation, the QRS complex reflects ventricular depolarisation, and the T wave mirrors ventricular repolarization (1). By extracting these waves and related pauses (ST, PQ, and TP segments), we can objectively quantify key aspects of the heart’s electrophysiological function (1). These measurements prove instrumental in characterising deviations from normal sinus rhythm, such as the absence of a P wave in ventricular rhythms or ST segment elevation/depression indicative of myocardial infarction (1). Furthermore, these measurements serve as critical inputs for diagnostic algorithms (2), acting either as clinical thresholds signalling abnormalities or as features for training and testing machine learning models (3). In the realm of ECG analysis, precise and automated measurement of these waves could revolutionise the development of more accurate decision support systems. This involves aggregating information from multiple-lead registries over several heart cycles, a labour-intensive task that currently hinders the efficiency of cardiologists’ workflows (3).

Many computational approaches exist for the automatic quantification of the ECG. Most of these produce detection and delineation of the electrocardiogram. Detection and delineation methods can be divided in two main groups: digital signal processing (DSP) and machine learning (ML) based methods. The latter can be further subdivided into deep learning (DL) and non-DL (hereinafter “hand-crafted”) methods.

Digital signal processing methods (4–8) have the advantage of explicitly imposing priors. Recently, Pilia et al. (9) released ECGdeli, an open source ECG delineation toolbox with state-of-the-art DSP techniques. These methods, however, often generalise poorly to unseen morphologies given their dependence on engineered transformation and rule-based aggregation steps (3), thus becoming more difficult to maintain.

Machine learning methods, on the other hand, have different associated problems that hinder their widespread adoption. Hand-crafted ML algorithms (10, 11) are difficult to train when using large amounts of annotated samples, and usually provide reduced performance as compared to well-tuned DSP-based or DL-based solutions. The reason for this is that feature engineering, a key step in hand-crafted ML-based solutions, is costly and difficult to produce in a robust, fast and comprehensive manner (12).

Various Deep Learning (DL) methods have emerged for ECG data processing, encompassing applications in cardiovascular disease diagnosis, blood pressure estimation, sleep analysis, and broader clinical analysis (13). Pioneering efforts by (14, 15) proposed a convolutional neural network (CNN) with varied kernel sizes to annotate ECG waves on the QT database (16). Subsequently, (17, 18) opted for networks with bilateral long short-term memory (BiLSTM) modules, emphasising the capture of temporal features. Demonstrating superior performance, Peimankar and Puthusserypady (19) advocated for an end-to-end model combining CNN with LSTM, a strategy subsequently embraced by others, yielding excellent results (20, 21).

Noteworthy variations include the replacement of CNN modules with dilated convolution (22) or the integration of residual neural networks (ResNet) (23). More recently, the incorporation of transformers has been explored (24, 25). In our earlier works (26, 27), we proposed an alternative using the U-Net architecture (28) to guide the ECG detection and delineation algorithm. This approach, also tested by others (29), yielded comparable results with robust generalisation capabilities.

However, DL-based methods provide black-box solutions that are difficult to verify, require large amounts of annotated data, have difficulties leveraging *a priori* information, and need quality loss functions for obtaining sensible data representations (3, 30–32). Moreover, both hand-crafted- and DL-based algorithms face difficulties when learning ECG data, given its high beat-to-beat morphological similarity and the small size of current ECG databases for their usage in data-driven approaches.

The main goal of this work was to develop an ECG detection and delineation algorithm addressing the aforementioned issues associated to DL-based data analysis. Firstly, we developed a novel synthetic data generation method for augmenting the database size with *a priori* information on normal and pathological ECG behaviour. Secondly, two loss functions were developed: the BoundaryLoss, which provide enhanced pixel accuracy close to the segmentation borders and is similar to other approaches in the literature (33, 34), and the F1InstanceLoss, which promotes cohesiveness in the predicted pixels regions. Lastly, we explored different modifications on the base U-Net architecture, namely different connectivity patterns such as the W-Net (35, 36), attention-based mechanisms (37) and different number of pooling operations. To the best of our knowledge, these improvements have not been explored in the literature for ECG analysis. A more rudimentary version of this work exists in the literature (27); however, the current approach displays key components that allow the algorithm to generalise better against a wider array of morphologies.

The rest of the paper is organised as follows. Section 2. describes the databases and methodology employed. Section 3. summarises the main results. Finally, Section 4. discusses the obtained results in their context. More details on the rules followed for the synthetic data augmentation procedure are included in the Supplementary Material, together with several examples on the performance of the developed algorithm on real-world clinical data from different pathologies.

## 2 Materials and methods

This section firstly describes the used databases in Section 2.1 for then defining the methodology employed for their analysis. The proposed methodology, on its behalf, can be divided into several steps. The first step consists in the pseudo-synthetic ECG generation from fundamental segments from a probabilistic rule-based algorithm (Section 2.2). The second step involve the definition and training of a deep learning architecture, which is subdivided into the description of the architecture itself (Section 2.3) and the employed loss functions (Section 2.4). Finally, the evaluation metrics are described in Section 2.5. A final section was added for detailing the specific experiments performed (Section 2.6). Our code has beeen made publicly available in https://github.com/guillermo-jimenez/DelineatorSwitchAndCompose.

### 2.1 Databases

The QT (16), the LU (38) and the database from the Ningbo First Hospital of Zhejiang University (39) (hereinafter the “Zhejiang” database) were employed for model training and evaluation. Specifically: the QT database was used for synthetic data generation and model training; the LU database, for synthetic data generation and evaluation; and the Zhejiang database, for model testing. The QT database contains 105 two-lead ambulatory recordings of 15 min sampled at 250 Hz, representing different pathologies (arrhythmia, ischaemic/non-ischaemic ST episodes, slow ST level drift, transient ST depression and sudden cardiac death) as well as normal sinus rhythm. The LU database is composed of 200 12-lead recordings of 10 s of length, sampled at 500 Hz, comprising sinus and abnormal rhythms as well as a variety of pathologies. The Zhejiang database, on its behalf, includes 334 12-lead outflow tract ventricular arrhythmias (OTVA) recordings of variable size (2.8–22.6 s), sampled at 2,000 Hz, and was originally devised for identifying the OTVA site of origin, containing no delineation annotations. These databases are an appropriate sample for testing generalisability, since they present heterogeneity in their represented pathologies, sampling rates, lead configurations (Holter and standard 12-leads) and centres of acquisition.

**Figure 1:**
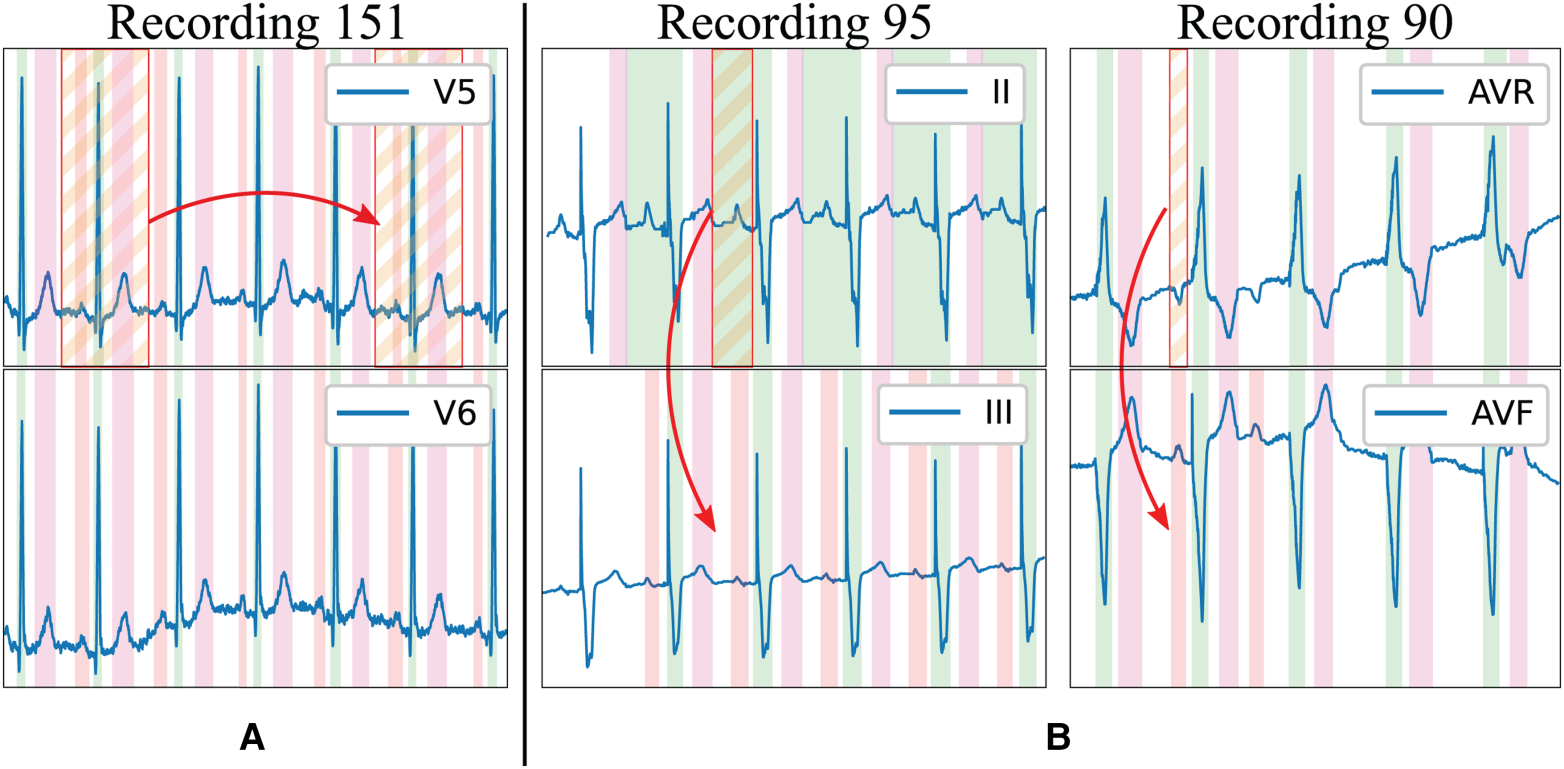
Limitations of existing delineation databases for training deep learning models. Examples in the LU database: (**A**) High beat-to-beat redundancy within recordings; (**B**) Incorrectly-annotated ground truth (top lead) and correction (bottom lead). Colour code: P wave (red), QRS wave (green) and T wave (magenta). Stripped segments highlight the errors.

The existing delineation databases have certain characteristics that hinder the development of reliable delineation algorithms. On the first hand, although they contain a relatively large amount of delineated cardiac cycles (3,528 and 1,830 annotated beats in the QT and LU databases, respectively), these present a high intra- and inter-patient redundancy (i.e., very similar morphologies in different patients for certain pathologies or during sinus rhythm and very stable ECG beat-to-beat morphology in the same trace), which complicates model training due to reduced population variability (Figure 1A). Moreover, given the difficulty and time-consuming process of delineating an ECG, some registries present delineation errors such as skipped beats or inconsistent onset/offset predictions for similar morphologies, among others. Those problems were addressed in two ways. Firstly, those outlier beats were re-annotated when necessary with the help of an expert cardiologist. Secondly, new ground truth was generated for the Zhejiang database, which was not annotated for delineation purposes, and reserved for algorithm testing as an independent set. All these new annotations have been added as Supplementary Material in the digital version. Some examples of annotation corrections can be seen in Figure 1B.

The data and ground truth, either real or synthesised, were then represented as binary masks for their usage in DL-based segmentation architectures, where a mask of shape $\{0,1{\}}^{3\times N}$ was True-valued whenever a specific sample n∈N was contained within a P, QRS or T wave (indices 0, 1 and 2, respectively) and False-valued otherwise (27). Finally, the joint training database was split into 5-fold cross-validation with strict subject-wise splitting, not sharing beats or leads of the same patient in the training and validation sets (27, 40). Given that the proposed method employs pseudo-synthetic data generation, the pseudo-ECGs were also generated using data uniquely from the training set for each fold, ensuring no cross-fold contamination.

### 2.2 Synthetic data generation

The structure of an ECG can be regarded as a combination of the P, QRS and T segments, alongside the PQ, ST and TP pause segments, which represent different phases of the electrical activation of the heart. The ECG is able to represent in its trace many pathological and non-pathological changes, reflecting slight deviations in its different constituting segments. The resulting “modular” structure can be leveraged in data-driven approaches for generating pseudo-synthetic data.

**Figure 2:**
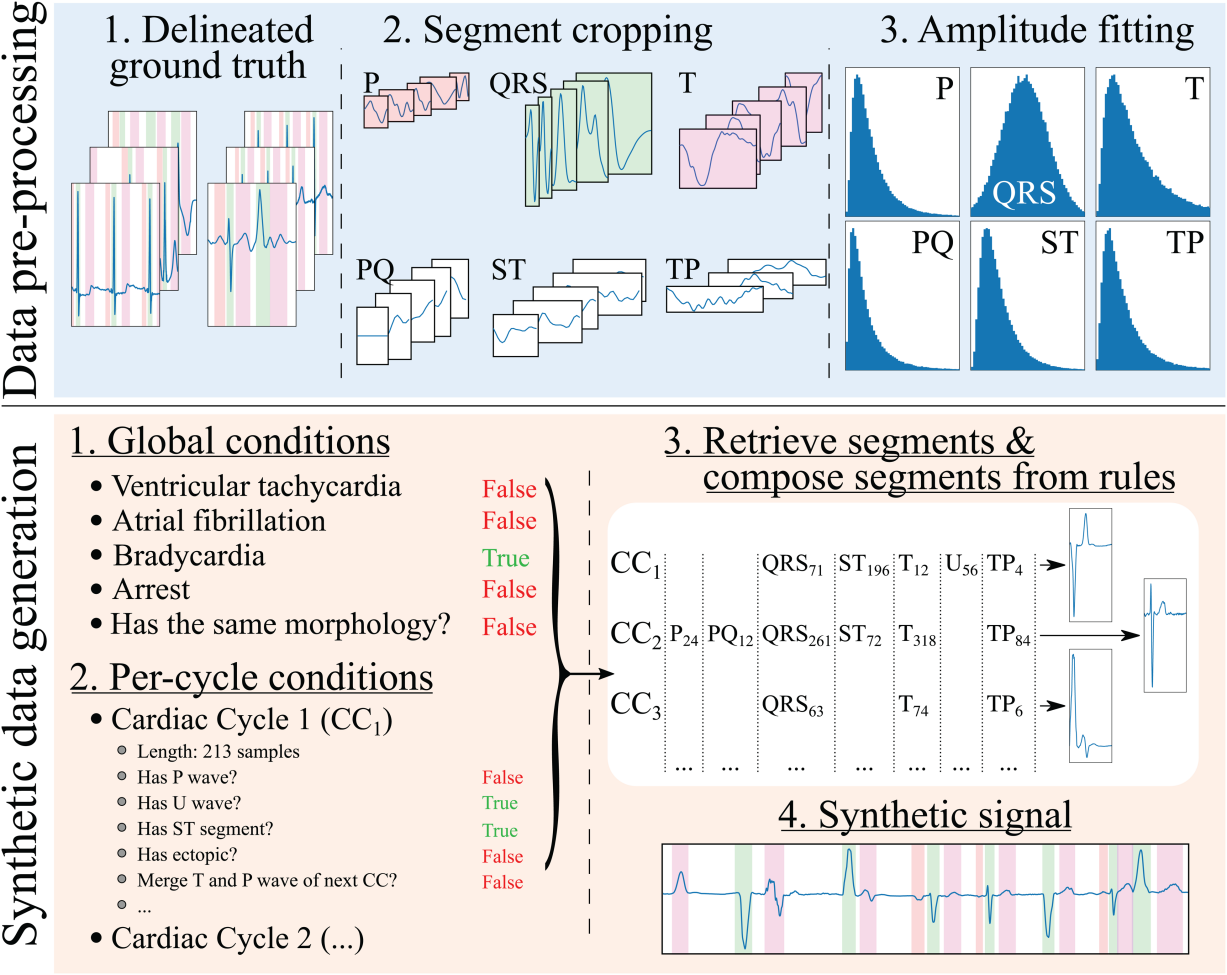
Synthetic electrocardiogam generation pipeline. The data pre-processing step consists in: (1) delineating the ground truth; (2) cropping the different beats contained in the ground truth into their constituent segments (P, PQ, QRS, ST, T and TP), normalising the QRS segment to have an absolute amplitude of 1, and normalising the rest of the segments’ as the amplitude fraction with respect to their (normalised) relative QRS; and (3) fitting the amplitudes to a normal distribution for the QRS wave (fraction with respect to its original amplitude) and log-normal distributions for the rest of the segments (fraction with respect to the QRS’ amplitude). The synthetic data generation step, on its behalf, involves: (1) producing a set of global rules that will be common for all synthesised cardiac cycles (in the example, the registry has bradycardia); (2) producing a set of rules that will affect each cardiac cycle individually (in the example, the first cardiac cycle, ${\mathrm{CC}}_{1}$, skips its P wave to simulate a ventricularly-mediated beat or a very low amplitude P wave); (3) retrieving the specific segments and their amplitude relationships from the “bags” of cropped segments for their composition into independent cardiac cycles; and (4) concatenating the segments into the final synthetic trace.

The developed generation pipeline, depicted in Figure 2, consisted in two main stages: a pre-processing step that prepared the data for its posterior usage and a data generation step that created synthetic ECG traces through composing independently generated cardiac cycles. The data pre-processing step, on its behalf, involved cropping the delineated ground truth (in this case, the QT and LU databases) in its constituent segments and into separate “pools” of segments from which to draw in subsequent stages. Additionally, the segment’s amplitude (relative to their associated QRS) was fitted into independent log-normal distributions, which would be sampled from in the generation step to relate the amplitude of each segment to the amplitude of the QRS in each cardiac cycle. The QRS segment amplitude was normalised with respect to the maximum QRS amplitude in the whole registry (comprising all leads).

The synthetic data generation step has several sub-steps. First, a set of global generation rules that affect all generated cardiac cycles were probabilistically generated for each sample. These have been limited to ventricular tachycardia (VT), atrial fibrillation (AF), atrioventricular (AV) blocks, sinus arrest (and its duration) and ST elevation/depression as a proof of concept. Second, a set of per-cardiac-cycle rules were generated, such as the presence or absence of each specific segment (P, QRS+T, PQ, ST, TP and U), whether the cycle corresponded to a ventricular ectopic (larger QRS amplitude and duration, absence of P wave) or whether there was wave merging (P with QRS, QRS with T, T with the next cycle’s P). In the first and second steps, the rules were defined by drawing samples from a uniform distribution and applying the associated operation (global in the first case and per-cycle in the latter) in case they surpassed a pre-defined threshold.

Third, a set of segments were randomly selected from the segment “pools.” A set of operations were then applied when extracting the segments from the pools as well as on the resulting cardiac cycles to comply with the global and per-cycle conditions. In particular, these operations comprised setting the segment’s amplitude, interpolating the segment to a randomised number of samples to enforce as much variability as possible, cropping the segment, merging of the segment with the next (e.g., merging the T and the P waves, thus enforcing TP segment suppression), sign-correcting the segment to match other cardiac cycles or applying per-segment elevation/depression.

Finally, the final synthetic signal and the ground truth were composed from the individual cardiac cycles. A set of post-composition operations were added to further increase the generated signal’s variability, consisting in adding baseline wander noise, interpolating to slower or faster rhythms, adding flat line noise at the signal’s edge, setting the global amplitude (multiplying the amplitude by a factor) and defining the trace’s starting segment.

**Figure 3:**
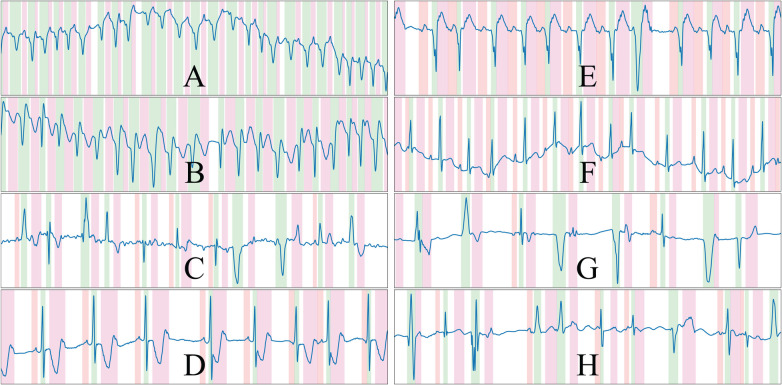
Randomly drawn samples from the developed synthetic data generator. The generator is able to produce samples of a variety of conditions such as ventricular tachycardia or atrial fibrillation, among others. The samples presented display ventricular ectopics (**C**,**G**,**H**), sinus rhythm (**D**,**E**,**F**), atrial fibrillation (**C**) and ventricular tachycardia (**A**,**B**), and are generated alongside their ground truth. Colour code: P wave (red), QRS wave (green) and T wave (magenta).

An important aspect to pseudo-synthetic ECG generation is efficiency, as the samples were generated online rather than offline to avoid restricting the approach to a fixed set of previously drawn samples. This is, however, only relevant during the training phases of the model, but can limit the options of operations that can be performed on the algorithm; in fact, many of the chosen additions were limited in their scope by this constraint, being restricted sometimes to oversimplified operations that offer close-enough approximations of the underlying represented cardiac conditions. Some randomly drawn samples from the synthetic data generator are shown in Figure 3. The complete list of rules is described in the Supplementary Material.

### 2.3 Architecture

**Figure 4:**
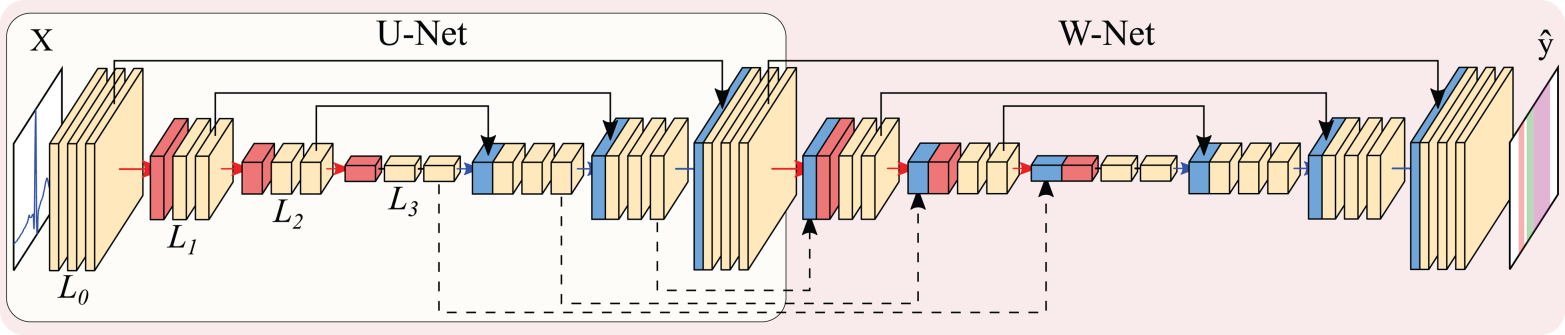
Representation of the U-Net (encircled in yellow) and W-Net architectures (encircled in red, containing the U-Net). Both networks are instantiated with 3 levels and 2 convolutional blocks per level. Arrows represent operations, while blocks are indicative of output tensors. Convolutional filters are doubled at each level, so that level $L_{i}$ has $2^{\text{i}}N$ channels per level (with N being the starting number of channels), whereas pooling and upsampling have a kernel size of 2. Colour code: convolutions (yellow), pooling operations (red), upsampling operations (blue), concatenation operations (black).

The U-Net (28) is a convolutional neural network is an encoder-decoder structure, as depicted in Figure 4. The encoder extracts high-level representations of the input data by means of convolutional operations, which transform an input tensor by convolving it with a trainable kernel, and pooling operations, which allow for reducing computational complexity. The decoder, on the other hand, upsamples the high-level encoder tensor to recover the original input’s resolution while aggregating partial results obtained in different levels of the encoder. This direct feature aggregation between the encoder and the decoder, in the shape of tensor concatenation, allows for finer border definitions while avoiding gradient vanishing problems (28). As in the original article, the number of trainable convolutional filters is doubled after every pooling operation and halved after every upsampling operation.

Many authors have experimented with the hyperparameters governing the U-Net, in the shape of number of convolutional operations (width), the number of upsampling-downsampling pairs (depth), starting number of convolutional filters, type of convolutional operation, type of non-linearity and presence/absence of other post-convolutional operations [batch normalisation (41), spatial dropout (42)], among others, which was partially covered in (27) for the QT database.

Other authors have explored refining further architectural changes. Given the myriad of options, we restricted the exploration to the application of the W-Net architecture due to its good performance in other segmentation domains (43) as well as the usage of self-attention mechanisms in the shape of efficient channel attention (ECA). The W-Net (35, 36) involves the application of a second U-Net whose input is the output of the first U-Net, thus approximately doubling the amount of parameters for the same number of initial channels. The W-Net also concatenates the tensors at the decoder of the first U-Net with the encoder of the second, similarly to the connections established between the encoder and the decoder of a “vanilla” U-Net. This secondary structure makes the network deeper, which usually presents increased performance (44). Self-attention applies the attention mechanism to a tensor, thus allowing different elements of the tensor to evaluate their relative importance for obtaining a certain result. This usually improves overall model performance and explainability (45). ECA, specifically, is an approach to apply this mechanism to CNNs in an efficient manner (37).

### 2.4 Loss functions

Two novel loss functions, the BoundaryLoss and the F1InstanceLoss, were developed with the objective of enhancing the resulting prediction accuracy in two ways: the F1InstanceLoss enforces the retrieval of connected structures so that a penalty term is induced if the number of predicted and present structures differ; the BoundaryLoss attempts at adapting more tightly to the target boundary by means of computing the intersection-over-union of a subset of the original samples present in a mask, as opposed to the usual Dice score computation. These losses were based on the application of edge detectors, allowing automatic differentiation for posterior gradient propagation.

The first step consisted in applying the edge detector along all non-batch and non-channel axes of the input tensors, isolating the segmentation boundary. In the case of the BoundaryLoss, a large kernel size is employed ($K\in R^{n}$, n being an hyper-parameter), whereas in the F1InstanceLoss the kernel size remains small ($K\in R^{3}$). In this case the Prewitt operator was employed as the edge detector, which is defined as:

(1) $$\;{\vec{K}}_{F_{1}}={\left(\begin{array}{ccc} -1 & 0 & +1 \end{array}\right)}^{T},\;{\vec{K}}_{\mathrm{Bound}}={\left(\begin{array}{ccccc} -1 & 0 & \cdots & 0 & +1 \end{array}\right)}^{T}.$$

The second step took the absolute of the edge-detected tensors for both the predicted and the ground truth masks. In the case of the BoundaryLoss, the third step involved the calculation of the Dice coefficient between the resulting tensors. This has the advantage of comparing the mask overlap on a reduced pool of pixels, increasing the precision at the segmentation boundary, as it is the case in image processing pipelines. In the case of the F1InstanceLoss, the third step was based on summing the border activations along each non-batch and non-channel axis separately for both the predicted and ground truth tensors, obtaining the number of discontinuities present in the binary mask. These discontinuities act as surrogates of the onset/offset pairs of the binary masks, thus allowing the computation of the number of predicted and ground truth elements ($P_{\mathrm{elem}}$ and $GT_{\mathrm{elem}}$, respectively) for computing precision and recall metrics in a fully differentiable manner. The true positive ($TP_{\mathrm{loss}}$), false positive ($FP_{\mathrm{loss}}$) and false negative ($FN_{\mathrm{loss}}$) loss metrics to compute the F1InstanceLoss are then computed by clamping these values, so that:

(2) $$\begin{array}{cc} & TP_{\mathrm{loss}}=\left|GT_{\mathrm{elem}}-\max(GT_{\mathrm{elem}}-P_{\mathrm{elem}},\;0)\right|\\ & \;FP_{\mathrm{loss}}=\max(P_{\mathrm{elem}}-GT_{\mathrm{elem}},\;0)\\ & \;FN_{\mathrm{loss}}=\max(GT_{\mathrm{elem}}-P_{\mathrm{elem}},\;0) \end{array}$$

**Figure 5:**
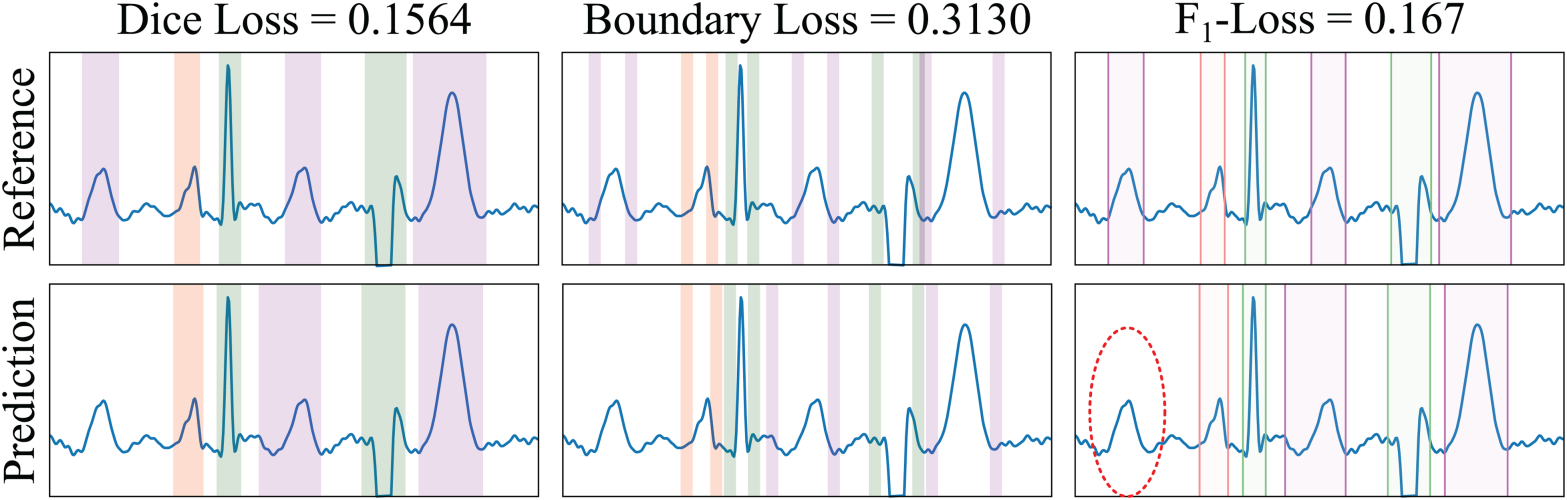
Example of loss functions applied to a sample from the LU database. The Dice Loss measures the overlap between the ground truth reference (GT, top row) and the predicted (bottom row) masks of the different electrocardiogram segments (differently coloured). The BoundaryLoss computes a secondary mask (with two parts for each ECG segment) for isolating samples surrounding the boundaries (i.e., onset and offset) of the GT and predictions, thus more specifically penalising onsets/offset errors. The $F_{1}$-InstanceLoss locates the onset/offset pairs of the masks to estimate and penalise differences in the number of ECG segments between the reference and the prediction. In the example, the ground truth contains three T waves (magenta), whereas only two T waves have been predicted; the $F_{1}$-score loss for each individual wave is 0, 0 and 0.167, for the P, QRS and T waves, respectively (thus having a penalty loss for the un-matching in the T waves). Colour code: P wave (red), QRS wave (green) and T wave (magenta).

Finally, the $TP_{\mathrm{loss}}$, $FP_{\mathrm{loss}}$ and $FN_{\mathrm{loss}}$ values were then used to compute the smoothed $F_{1}$-InstanceLoss between the input and target masks. The computation process of these loss functions is depicted in Figure 5.

### 2.5 Evaluation

The model evaluation is based on the computation of detection metrics, i.e., the model’s precision, recall and $F_{1}$-Score, and delineation metrics, i.e., onset and offset errors on the true positives (mean, M ± standard deviation, SD). The computation of the metrics consisted in three steps. Firstly, the onset and offset fiducials were retrieved from the predicted binary mask (described in Section 2.1) to express the sample of occurrence by retrieving the locations of value change (False to True or vice-versa). Secondly, the ground truth and predicted fiducials were matched through a correspondence matrix. Thirdly, the correspondence matrix was used to compute the detection and delineation metrics.

The correspondence matrices between the true (P, QRS and T) and predicted ($\hat{P}$, $\hat{QRS}$ and $\hat{T}$) fiducials were computed as:

(3) $$\begin{array}{ccc} \;P_{\mathrm{ij}} & \;= & \;\{\begin{array}{c} 1\\ 0 \end{array}\begin{array}{c} \mathrm{if}\\ \mathrm{or}\\ \mathrm{otherwise} \end{array}\begin{array}{c} {\overset{⌢}{P}}_{\mathrm{fid}}[j]\in [P_{\mathrm{on}}[i],P_{\mathrm{off}}[i]]\;\\ P_{\mathrm{fid}}[j]\in [{\overset{⌢}{P}}_{\mathrm{on}}[i],{\overset{⌢}{P}}_{\mathrm{off}}[i]]\;\\ \; \end{array}\;\;\;\;\;\;\;\\ \;QRS_{\mathrm{ij}} & \;= & \;\{\begin{array}{c} 1\;\\ \;\\ 0\; \end{array}\begin{array}{c} \mathrm{if}\\ \mathrm{or}\\ \mathrm{otherwise} \end{array}\begin{array}{c} Q\overset{⌢}{R}S_{\mathrm{fid}}[j]\in [QRS_{\mathrm{on}}[i],QRS_{\mathrm{off}}[i]]\;\\ QRS_{\mathrm{fid}}[j]\in [Q\overset{⌢}{R}S_{\mathrm{on}}[i],Q\overset{⌢}{R}S_{\mathrm{off}}[i]]\;\\ \; \end{array}\\ \;T_{\mathrm{ij}} & \;= & \;\{\begin{array}{c} 1\;\\ \;\\ 0\; \end{array}\begin{array}{c} \mathrm{if}\\ \mathrm{or}\\ \mathrm{otherwise} \end{array}\begin{array}{c} {\overset{⌢}{T}}_{\mathrm{fid}}[j]\in [T_{\mathrm{on}}[i],T_{\mathrm{off}}[i]]\;\\ T_{\mathrm{fid}}[j]\in [{\overset{⌢}{T}}_{\mathrm{on}}[i],{\overset{⌢}{T}}_{\mathrm{off}}[i]]\;\\ \; \end{array}\;\;\;\;\; \end{array}$$

where fid∈{on,peak,off} is the specific fiducial to be explored, and i∈[0,M], j∈[0,N] are the total true and predicted fiducials for each of the waves, respectively.

These correspondence matrices were used to obtain the detection and delineation metrics. The detection metrics (true positives, TP, false positives, FP, and false negatives, FN) were computed as follows: given a correspondence matrix H, true positives were computed as elements that have been matched ($\mathrm{TP}=\sum H_{\mathrm{ij}}$); false positives were elements of a predicted fiducial that did not match any element in the ground truth, corresponding to the difference between the number of predicted fiducials and the cardinality of the matches ($\mathrm{FP}=N-card(\{(i,j)|H_{\mathrm{ij}}=1\})$); and false negatives were computed as elements of the ground truth that did not match any true fiducial, corresponding to the difference between the number of true fiducials and the cardinality of the matches ($\mathrm{FP}=M-card(\{(i,j)|H_{\mathrm{ij}}=1\})$). The TP, FP and FN were in turn used to compute the model’s precision (Pr), recall (Re) and $F_{1}$-score. The delineation error, on its behalf, was computed through the mean and standard deviation (SD) of the difference of the actual and predicted onsets and offsets of the TP in the correspondence matrix:

(4) $$\min_{i,j}\;\;w_{\mathrm{fid}}[i]-{\tilde{w}}_{\mathrm{fid}}[j]\;s.t.\;H_{\mathrm{ij}}=1.$$

These metrics were employed in turn for assessing the performance on the QT, LU and Zhejiang databases. In the case of the QT database, to homogenise the evaluation criteria with the existing literature, the detection and delineation metrics were computed for single-lead and multi-lead approaches, where the single-lead is based on evaluating the performance of both leads in the Holter registry independently, and the multi-lead consists in taking, for each beat, the lead that produces the best adjustment. Contrarily, the LU and Zhejiang databases were evaluated by fusing the individual lead predictions to obtain a single output prediction for, subsequently, comparing this delineation with the annotated ground truth. The final prediction was computed through combining the individual lead results using majority voting of the 12 leads and the different models resulting from training on separate folds of the QT database, forming an ensemble.

Finally, these metrics were also used to define the “best” performing model, which was selected as the one producing good detection performance while attaining the lowest possible delineation error for the QT (in the validation fold), LU and Zhejiang databases. This was addressed through the calculation of two figures of merit: the largest $F_{1}$-score as detection performance and the smallest SD of the error as delineation performance for all three databases across all waves, and reported in Section 3.1. Moreover, this model ranking was employed for producing ablations of the different modifications (Section 2.) by isolating a single modified factor while leaving the rest of the hyperparameters unmodified. These have been reported in Section 3.2.

### 2.6 Experiments

The model’s performance was tested under an array of complementary tests to address the contributions of the different elements to the results. Firstly, the importance of the synthetic data augmentation was addressed by training the same model architecture using augmented data (real and synthetic), synthetic-only data and real-only data. Identical computational budget was ensured by producing the same number of batches (with identical batch size) for the same number of epochs by oversampling the training database. Secondly, the importance of the BoundaryLoss and F1InstanceLoss was addressed also by doubling the number of executions, with and without the proposed losses. The Dice score always remained as a baseline for training in every configuration. Finally, the importance of the architectural modifications was addressed. Several architectures were tested: U-Net for depths 5, 6 and 7; W-Net for depths 5 and 6; and W-Net with ECA for depth 5. In all cases, the number of input channels was kept the same in the W-Net as in its U-Net counterpart, resulting in models with increased number of parameters (capacity). These were selected to have as many candidate architectures as possible but without compromising the computational budget of our equipment. In total, 66 different configurations were tested to address the model’s performance.

Some design choices were kept constant to avoid unfeasibly large hyper-parameter exploration. All model configurations used the same random seed (123456), leaky ReLU non-linearities, zero padding for preserving tensor shape, kernels of size 3, batch normalisation, spatial dropout (42) (p=0.25), Adam optimiser (46) (lr=0.001) and the Dice loss alongside the developed losses. The BoundaryLoss employed a kernel size of 11 samples. The ordering of operations after the convolutional operations was defined to agree with the image segmentation literature (non-linearity → batch normalisation → dropout) (47, 48). All networks were trained using ECG-centred data augmentation, as described elsewhere (27), comprising additive white Gaussian noise, random periodic spikes, amplifier saturation, power-line noise, baseline wander and pacemaker spikes to enhance the model’s generalisability. All executions were performed at the Universitat Pompeu Fabra’s high performance computing environment, assigning the jobs to either an NVIDIA 1080Ti or NVIDIA Titan Xp GPU, and used the PyTorch library (49).

## 3 Results

### 3.1 Best performing model

The best performing model according to the criteria presented in Section 2.5 was a self-attention W-Net model with 5 levels, trained with both real and synthetic data, while excluding the F1InstanceLoss and the BoundaryLoss (around 548 k parameters; see a figure with training and validation losses as Supplementary Material). The model obtained an average $F_{1}$-score of 99.38% and a average delineation error of 2.19±17.73 ms and 4.45±18.32 ms for the onsets and offsets, respectively, across all waves and databases. The per-database and per-wave metrics of the model (precision, recall, onset error and offset error) were reported in Tables 1, 2 for completeness. Examples of ECG delineation with the best performing model in private datasets of patients with different pathologies (e.g., intrauterine growth restriction, hypertrophic cardiomyopathy, Tetralogy of Fallot, Brugada syndrome) are shown in Supplementary Material, demonstrating the generalisation of the developed methodology.

**Table 1:** Precision (Pr, %), recall (Re, %), F1 score, onset error (OnE, mean [M] ± standard deviation [SD], in milliseconds) and offset errors (OffE, M ± SD, in milliseconds) of our best performing single-lead (SL) and multi-lead (ML) models in the QT database. N/R stands for “not reported.” Bold values represent best performance for each fiducial. Ref1: (27). Ref2: (8). Ref3: (22).

		This work (SL)	This work (ML)	Ref1 (SL)	Ref1 (ML)	Ref2	Ref3
	Pr	**99.27**	98.90	90.12	94.17	91.03	94.39
	Re	98.38	**99.72**	98.73	94.70	98.87	92.66
P wave	F1	98.82	**99.31**	–	–	–	–
	OnE	−1.2 ± 17.9	**−0.8 ± 13.5**	1.5 ± 22.9	−1.7 ± 17.8	2.0 ± 14.8	7.01 ± 6.23
	OffE	1.1 ± 16.6	**−0.6 ± 12.7**	0.3 ± 16.0	4.0 ± 16.1	1.9 ± 12.8	6.59 ± 5.54
	Pr	99.31	99.24	99.14	99.40	**99.86**	97.04
	Re	99.94	**99.97**	99.94	99.28	99.80	95.29
QRS wave	F1	99.62	**99.60**	–	–	–	–
	OnE	−0.5 ± 11.2	**0.1 ± 7.5**	−0.1 ± 8.4	−3.8 ± 14.6	4.6 ± 7.7	4.61 ± 4.99
	OffE	3.7 ± 13.1	**1.7 ± 7.8**	3.6 ± 12.6	5.4 ± 16.8	0.8 ± 8.7	4.77 ± 4.65
	Pr	**98.73**	98.24	98.25	96.36	97.79	94.06
	Re	99.78	**99.97**	99.88	99.09	99.77	92.30
T wave	F1	**99.25**	99.10	–	–	–	–
	OnE	5.8 ± 39.6	**5.2 ± 31.1**	21.6 ± 66.3	19.1 ± 66.5	N/R	18.23 ± 16.60
	OffE	2.4 ± 51.3	3.8 ± 37.2	4.6 ± 31.1	9.9 ± 46.3	**−1.6 ± 18.1**	10.15 ± 14.23

**Table 2:** Precision (Pr, %), recall (Re, %), F1 score, onset error (OnE, mean [M] ± standard deviation [SD], in milliseconds) and offset errors (OffE, M ± SD, in milliseconds) of our best performing model in the LU and Zhejiang databases, obtained through pixel-wise majority voting of the model developed for each fold trained on the QT database. Bold values represent best performance for each fiducial. Ref1: (6). Ref2: (7). Ref3: (29). Ref4: (50).

		Zhejiang (This work)	LU (This work)	LU (Ref1)	LU (Ref2)	LU (Ref3)	LU (Ref4)
	Pr	97.57	**99.62**	98.43	98.43	97.69	90.48
	Re	98.65	**99.81**	96.44	96.44	98.01	97.36
P wave	F1	98.11	**99.72**	–	–	97.85	–
	OnE	2.46 ± 12.58	8.23 ± 9.01	**2.2 ± 7.4**	2.8 ± 7.5	−0.6 ± 17.5	3.4 ± 18.4
	OffE	2.87 ± 12.43	3.01 ± 10.40	−6.5 ± 10.7	**−7.3 ± 10.1**	−2.4 ± 18.4	−4.1 ± 19.4
	Pr	99.53	**100.00**	100.0	99.56	99.93	98.27
	Re	99.87	**100.00**	99.86	99.86	100.0	99.86
QRS wave	F1	99.70	**100.00**	–	–	99.97	–
	OnE	4.72 ± 13.35	**4.27 ± 9.75**	15.4 ± 14.6	18.4 ± 14.7	1.5 ± 11.1	1.7 ± 10.0
	OffE	3.26 ± 11.91	**4.00± 9.14**	−3.8 ± 13.6	−5.4 ± 14.3	2.0 ± 10.6	−3.4 ± 12.3
	Pr	98.86	**100.00**	99.21	99.09	99.37	96.23
	Re	99.86	1**00.00**	98.85	98.85	99.68	93.51
T wave	F1	99.36	**100.00**	–	–	99.52	–
	OnE	8.73 ± 28.85	18.26 ± 18.21	**−1.3 ± 8.8**	−2.6 ± 11.4	2.9 ± 23.7	9.2 ± 28.2
	OffE	−3.77 ± 24.32	−8.84 ± 18.05	**−1.2 ± 6.8**	−3.3 ± 7.3	−2.4 ± 30.4	−6.0 ± 25.0

### 3.2 Performance comparison of model additions

**Figure 6:**
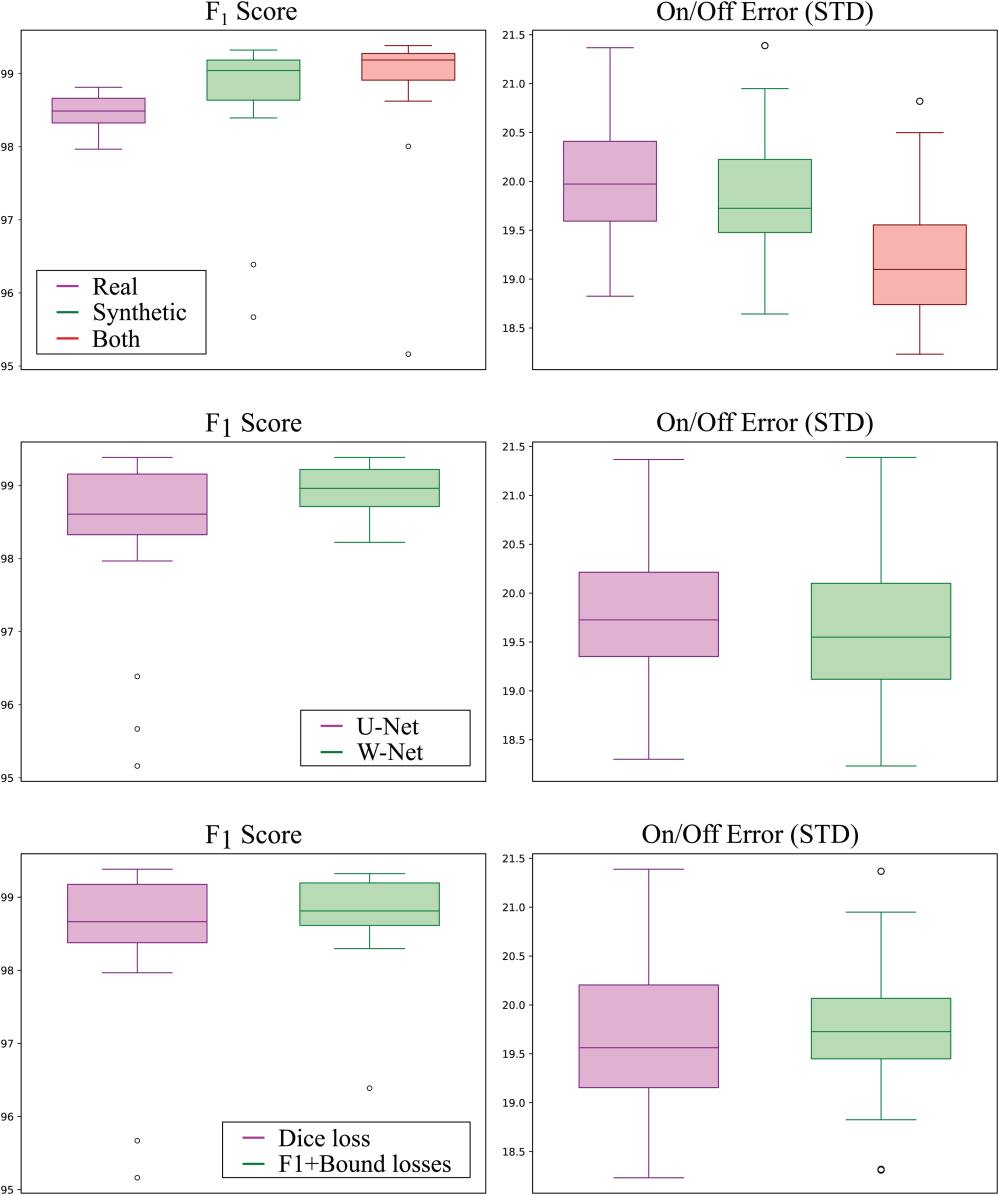
Detection (left; higher is better) and delineation (right; lower is better) performance of all models grouped by training data source (top row), model topology (middle row), and employed loss function (bottom row). Synthetic-only data (green) showed higher detection and delineation performance than real-only data (magenta), whereas using both sources produced the best results for both detection and delineation performance. The W-Net (green) showed slightly higher detection and delineation performance than the U-Net (magenta). Finally, using the $F_{1}$-InstanceLoss and BoundaryLoss (green) resulted in models with higher detection performance but slightly lower delineation performance as compared to using Dice loss only (magenta).

The best performing addition was including synthetic data, where the usage of both real and synthetic data reported an average increased $F_{1}$ score of 0.62% (p<0.05) and a reduced on/off error (p<0.01) with respect to using real data only. Interestingly, using synthetic data only for model training still produced increased performance over using only real data, surpassing its $F_{1}$ Score by 0.35% (although non-significantly, p=0.27) and reducing onset/offset error (p<0.01). Boxplots of the models grouped by data source can be visualised in Figure 6 (top).

The second-to-best model performance addition was the usage of the W-Net architecture, which produced 0.53% less $F_{1}$ error (p<0.05) and a non-statistically-significant reduction in its SD of 1.83 ms and 2.20 ms for the onset and offset metrics (p=0.19) respectively, as compared to its U-Net counterpart (see Figure 6). The addition of the F1InstanceLoss and the BoundaryLoss functions increased predictive performance of 0.26% $F_{1}$ score, and reduced the offset error in 0.07 ms, while increasing the onset error in 0.17 ms (Figure 6). However, these differences were not statistically significant (p=0.176 and p=0.772, respectively). The rest of the improvements (usage of self-attention, increase of model capacity) did not show a consistent effect on model performance.

## 4 Discussion

Analysing electrocardiograms (ECG) remains a pivotal task in hospitals, owing to its widespread availability and the valuable insights derived from the resulting non-invasive measurements. Traditionally, classical signal processing algorithms were employed for ECG analysis, offering accurate results in wave detection and delineation but exhibiting limited generalisation capabilities.

The advent of deep learning algorithms is revolutionising ECG analysis, paralleling advancements in other domains of data processing. Deep learning consistently outperforms many hand-crafted data analysis approaches across a range of tasks (51). Its strengths lie in the ability to harness extensive datasets, adaptability to diverse tasks, built-in feature engineering, and the availability of open-source code and large datasets (31). Despite these advantages, implementing deep learning in data-sensitive contexts, such as ECG detection and delineation, poses challenges. Firstly, these algorithms heavily rely on the size of the training data (3), which can be challenging or costly to obtain and annotate in clinical environments. Secondly, deep learning models encounter difficulties incorporating data priors—information that designers know should be integrated into the system. In the case of ECG, this includes nuances like the sometimes imperceptible amplitude of the P wave or its potential masking within a QRS complex, as well as the inherent relationship between the absence of a QRS complex and the absence of a T wave.

Several solutions exist to tackle these challenges. The issue of data scarcity has been addressed in the literature through the use of pseudo-labels (52) or synthetic data generation, achieved either via simulations (53, 54) or generative adversarial networks (GANs). However, these approaches face efficiency concerns in the case of simulations and encounter difficulties when extending beyond the training data manifold in data-driven methods. In terms of data priors, approaches have been employed to enforce representations that explicitly exclude known information (30). Alternatively, specific priors can be incorporated as input data, such as including labels as input in conditional GANs (55). Despite these strategies, the explicit control of data-side priors remains somewhat limited.

This work presents a DL-based algorithm for ECG detection and delineation that include innovative approaches to address these issues. Given the small size of the available ECG databases, the models were enriched with a novel synthetic data augmentation strategy, which allowed for imposing expert domain knowledge through constraining the topology of the generated data. These priors were further enforced in the shape of two novel loss functions by minimising the boundary error with respect to the reference (BoundaryLoss) and by maximising precision and recall metrics (F1InstanceLoss). To the best of our knowledge, no approaches for ECG detection and delineation exist in the literature that combine a quantification task through explicit (rule-based synthetic data generation) and implicit (application of the BoundaryLoss and F1InstanceLoss functions) prior imposition.

Performance-wise, the developed models compare favourably with existing DSP-based and DL-based approaches found in the literature. We obtained an average $F_{1}$ score of 99.38% and onset and offset errors of 2.19±17.73 ms and 4.45±18.32 ms with respect to the reference for all waves in the QT database, as detailed in Table 1. As illustrated in the table, it signifies a substantial improvement over our previous work without synthetic data generation and advanced losses, notably showcasing an 8% enhancement in P-wave precision and a reduction of onset error in the T-wave from 21.6±66.3 ms to 5.2±31.1, both for a single lead, among other notable examples. The remarkable boost in model performance can be directly attributed to the deliberate design decisions implemented. The incorporation of synthetic data emerged as the most impactful addition, consistently elevating model performance in comparisons between models trained with and without synthetic generation. Intriguingly, models exclusively trained with synthetic data outperformed those trained solely on real data, suggesting a superior capture of data variability in the synthetic database compared to the limited number of real cases. The adoption of the W-Net architecture emerged as the second most effective modification, contributing to an increased model capacity. Following closely, the introduction of novel loss functions stood out as the third most valuable addition. While this enhancement consistently improved model performance across all runs, it was not consistently present in the top-performing models. Further research is warranted to understand the mechanisms by which these loss functions enhance model performance.

**Figure 7:**
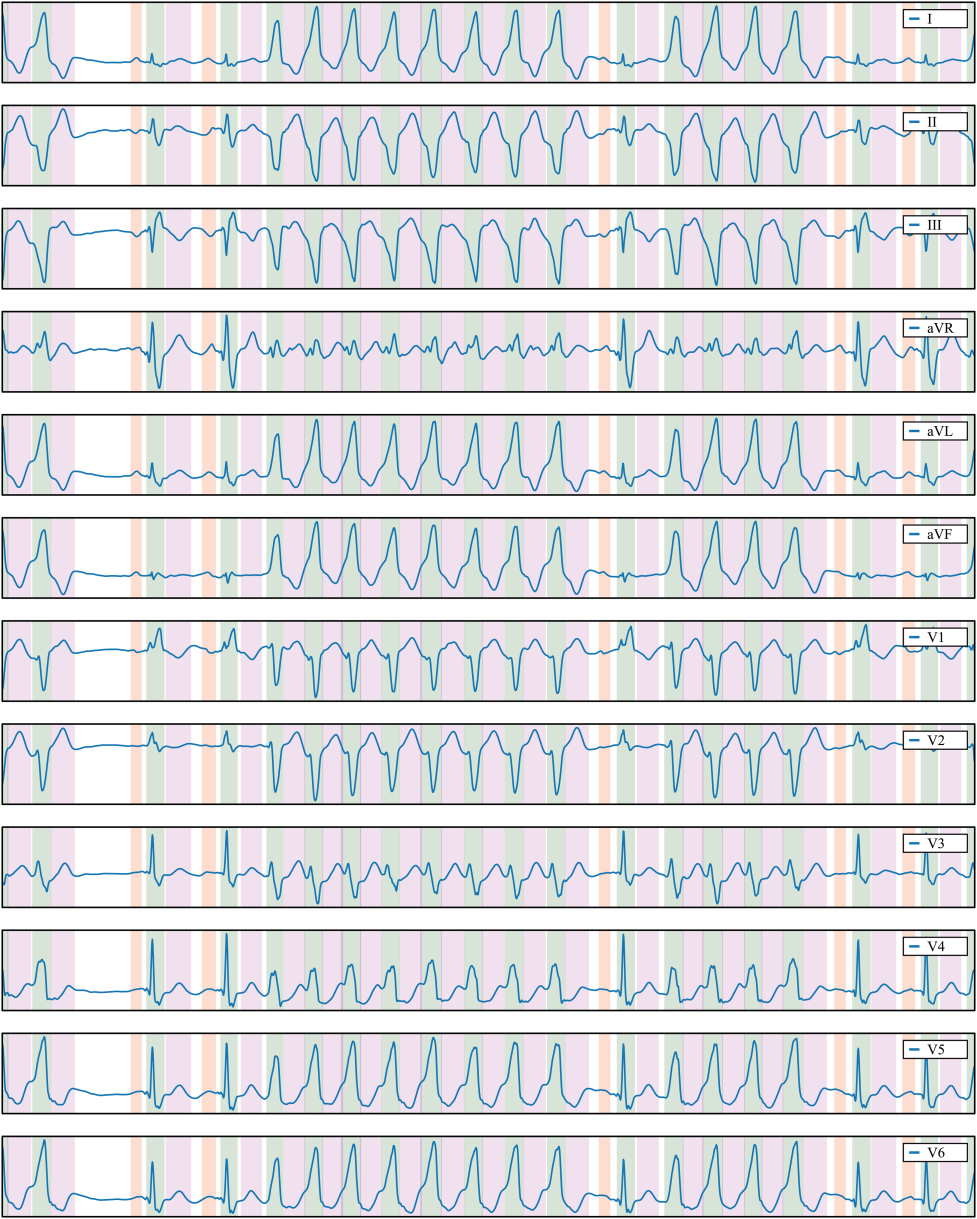
Delineation prediction of the sample “922551” of the Zhejiang database, containing a non-sustained ventricular tachycardia. Colour code: P wave (red), QRS wave (green) and T wave (magenta).

The trained model exhibits strong generalisation capabilities when applied to samples from the QT database (see Table 1), as well as in the LU (38) and Zhejiang (39) databases (see Table 2). Notably, its performance on the LU database achieves impressive $F_{1}$-scores of 100% for the QRS and T waves. This underscores the robustness of our model and the relatively straightforward nature of the rhythms represented in the LU database. Even when faced with the more challenging Zhejiang database, our model maintains a high level of performance, yielding metrics that closely resemble those obtained from the QT database. Figure 7 illustrates a prediction from the Zhejiang database. The examples available in the Supplementary Material also demonstrate that the developed methodology generalises well and can reliably be applied for the ECG detection and delineation for a wide range of different pathologies.

Our top-performing model compares favourably with the state-of-the-art in ECG detection and delineation, showcasing its accuracy when compared with reported results in the literature, particularly on the QT and LU databases. Notably, Darmawahyuni et al. (20) (Table 7 in their manuscript) recently demonstrated the superiority of our modelling strategy over various DL-based approaches, including different combinations of U-Net, ResNet, and LSTM networks (20, 23, 29), particularly on the LU database. Similarly, Liang et al. (22) showcased the superior performance of U-Net-based architectures, akin to ours, over CNN-BiLSTM approaches. Notably, even without the addition of synthetic data augmentation and advanced losses, our approach outshines others. However, recent work by Nurmaini et al. (21) achieved outstanding results with a Convolution BiLSTM, leveraging hyper-parameter tuning optimisation. They achieved a precision of 99.93% and recall of 99.92% for ECG waveform classification in a selected single lead (lead II). Our results, 99.87% precision and 99.93% recall in multi-lead, align closely with their achievements, outperforming alternative methodologies (refer to Table 8 in their manuscript). Notably, recent methods incorporating Transformers (24, 25) did not surpass the previous approaches: they yielded average $F_{1}$ scores of 98.69 and 94.62 for the QT and LU databases, respectively (25).

In our view, the performance reported by the majority of the compared methods, as assessed through metrics such as precision, recall, and $F_{1}$, is commendable and seems suitable for clinical decision-making. However, the inclusion of additional metrics, like onset/offset errors, often omitted in published literature (with Liang et al. (22), reported in Table 1, being a notable exception among recent DL-based papers on ECG detection and delineation), becomes imperative for comprehending the clinical significance of differences between methods.

For example, despite achieving 100% precision and accuracy for QRS detection in the LU database, our proposed method incurs an error of 4.27±9.75 ms and 4.00±9.14 ms for QRS onset and offset, respectively. Considering the current data sample rate of 250 Hz, a 4 ms difference corresponds to a single sample. These errors may be deemed acceptable for QRS width estimation, a parameter crucial in various clinical guidelines (e.g., a QRS width threshold of 120 ms for patient selection in cardiac resynchronization therapy). Conversely, errors in the range of 20–30 ms for T wave onsets/offsets may prove too substantial for specific clinical decisions.

In light of these considerations, it is imperative for the scientific community to collectively establish a consensus on the most pertinent and clinically relevant metrics for ECG detection and delineation. Such consensus is crucial for translating these metrics into daily clinical practice. Additionally, the availability of open-access databases (e.g., QT and LU databases) has played a pivotal role in benchmarking various approaches, significantly advancing progress in the field. Regrettably, only 11% of DL-based techniques for ECG processing, as highlighted in a recent review by Avula et al. (56), have embraced open access principles. To foster further research and encourage external adoption of our model, we have made the developed codes publicly accessible in the following repository: https://github.com/guillermo-jimenez/DelineatorSwitchAndCompose.

Despite the impressive results obtained, certain limitations accompany the presented approach. Firstly, the set of rules devised in the data generation process is somewhat restrictive. There exists potential to represent a broader spectrum of conditions and introduce more intricate modifications to fundamental ECG segments (e.g., incorporating delta, J, or epsilon waves, or simulating atrial/ventricular hypertrophy). Secondly, the computational overhead associated with on-the-fly data generation, coupled with prevalent computational and temporal constraints in the DL literature when training large models, has hindered exhaustive testing of each element’s contribution to the final outcome. This challenge is exacerbated by the multitude of tunable hyperparameters. Although the synthetic generator employs hyperparameters producing visually plausible samples, a comprehensive validation is yet to be conducted.

Additionally, despite dedicated efforts towards generating VT records and achieving success in a significant percentage of predictions, the network encounters challenges in accurately locating the onsets and offsets of very fast VTs/ventricular flutter. This limitation aligns with the inherent difficulty even trained physicians face in precisely delineating such occurrences. Moreover, the network exhibits sensitivity to input normalisation. Given that amplitude normalisation for sinus rhythm QRS is set to values in the range [0.5,1], larger values for other rhythms, such as extra-systoles, have been adopted. To address this, we have normalised the model’s input using the median of a moving average over the signal, employing a window of 256 samples. However, this criterion remains open for improvement. Future research will also explore the potential added value of incorporating temporal features through modules like LSTM or Transformers within the current model architecture.

## 5 Conclusions

The detection and delineation of electrocardiograms represent crucial clinical steps, and the application of deep learning techniques holds the promise of automating the often manual and subjective task of characterising ECG waves. Nevertheless, the adoption of DL-based analysis introduces challenges, including the interpretability issues associated with classification-based models, limitations imposed by reduced database sizes, and the need to establish effective data priors. In response, we have developed a DL-based pipeline tailored for the automatic quantification of electrocardiograms, incorporating innovative strategies such as synthetic data generation and shape regularisation losses to address these challenges.

The resulting network has showcased commendable metrics for detection and delineation, coupled with robust generalisation across diverse samples from various open-source databases and real-world datasets. This versatility positions the pipeline for application in a myriad of downstream tasks, facilitating the automated generation of objective metrics for clinical data and serving as a pivotal technology for advancing the automation of ECG analysis. However, it is essential to acknowledge certain limitations. Firstly, the synthetic data generation introduces a reliance on input data normalisation when predicting samples, albeit the commonly employed techniques such as windowing and normalisation to the median usually yield satisfactory results. Secondly, to enhance the versatility of the synthetic data generation algorithm, there is a need for a broader range of cardiac conditions and a more thorough exploration of generative parameters. Lastly, to comprehensively assess the performance gains of each model addition, a more exhaustive testing protocol could be explored.

## Data Availability

The databases used in this work are publicly available at https://physionet.org/content/qtdb/1.0.0/ (QT database), at http://www.cyberheart.unn.ru/database (LU database) and at https://doi.org/10.6084/m9.figshare.c.4668086.v2 (Zhejiang Hospital database). The ground truth revisions in the QT database and the annotations over the Zhejiang database have been uploaded to Figshare in Jimenez-Perez (57), Jimenez-Perez (58) and Jimenez-Perez (59). The tool employed for producing the revised ground truth annotations used the Bokeh python library Bokeh Development Team (60) and is available in the author's repository at https://github.com/guillermo-jimenez/QRSAnnotator.
