# Supplementary material for: Generalising electrocardiogram detection and delineation: training convolutional neural networks with synthetic data augmentation
Source: Front Cardiovasc Med. 2024 Jul 19;11:1341786. doi: 10.3389/fcvm.2024.1341786 (PMC11294154; doi:10.3389/fcvm.2024.1341786)
Supplement: Supplementary file 1 [file Datasheet1.zip › frontiers_SupplementaryMaterial.pdf]

Supplementary Materials of "Generalising electrocardiogram detection and delineation: training convolutional neural networks with synthetic data augmentation"

1 SYNTHETIC DATA AUGMENTATION RULES

In this section, the global and per-cycle rules mentioned in Section II.B and the default hyperparameters will be detailed. Where not stated, the uniform distribution is applied. Nomenclature:

SIGNAL Generated registry, as generated by composing the cardiac cycles.
N Registry's length.
M Number of cardiac cycles.
K Total number of generated segments.

1.1 Global rules

Global rules are those which affect all cardiac cycles (CC) within a generated registry. All the rules could be active at the same time. For this project, the following global rules were designed:

1. **(True/False; p $<$ 25%) Has the same morphology?**
   If set to True, the selected segments are the same for all CCs. If False, segments need not coincide for any CC. As an example, if True and if the selected P wave has the ID 128, all cardiac cycles have that same P wave.
2. **(True/False; p $<$ 5%) Does the registry flatline at any point?**
   If set to True, a flatline length is generated ($\text{FLAT\_LENGTH} = \text{N}/RandomInteger([2,5])$, where N is the registry's length) and set to start at a random location in $\text{FLAT\_ON} = RandomInteger([0, \text{FLAT\_LENGTH}])$. Once these parameters are generated, the amplitude values in the samples $[\text{FLAT\_ON}, \text{FLAT\_ON} + \text{FLAT\_LENGTH}]$ are replaced with an "edge" padding, repeating the last generated value ($\text{SIGNAL}[\text{FLAT\_ON} : \text{FLAT\_ON} + \text{FLAT\_LENGTH}] = \text{SIGNAL}[\text{FLAT\_ON}]$.
3. **(True/False; p $<$ 10%) Has atrial fibrillation (AF)?**
   If set to True, two events are triggered. On the one hand, all P waves are set to be the same for all CCs. On the other hand, after composing all CCs, an extra P wave is selected as a template and tiled to fill the signal's width, which is then added to the registry to simulate F waves.
4. **(True/False; p $<$ 10%) Has atrioventricular (AV) block?**
   If set to True, if Global rule [1] is set to True and the Global rules [3] and [6] are set to False, this global rule skips the PQ, QRS, ST and T segments of selected segments, resulting in some CCs that only contain P and TP (rest) segments only. Firstly, the periodicity of the AV block is defined (e.g. 1:1, 2:1, 3:1 AV block) so that $\text{N\_AV\_SKIPPED} = RandomInteger(1, 3)$ and $\text{N\_AV\_NORMAL} = RandomInteger(2, 5)$. Secondly, to avoid too short CCs, the length of each CC before discarding the PQ, QRS, ST and T segments is computed. Thirdly, the PQ,

QRS, ST and T segments of the marked CCs are set to be skipped. Fourthly, segment merging (see Per-cycle rules [1]) is disabled for the PQ, ST and TP segments where AV block is present. Finally, the previously computed CC length is used to substitute the TP segment by a random walk of said size.

5. **(True/False; p $<$ 10%) Has tachycardia?**
   If set to True, the following events are triggered. Firstly, the probability of merging the PQ, ST or TP segments is doubled (30%, 50% and 50%, respectively; see Per-cycle rules [1]). Secondly, a maximum TP segment length is established (maximum TP length of CC i is set to $\text{TP\_LENGTH}_i = RandomInteger([75, 125])$ samples). Thus, the TP segment is cropped to the aforementioned fixed length and zero-corrected.
6. **(True/False; p $<$ 10%) Has ventricular tachycardia (VT)?**
   If set to True, three events are triggered. Firstly, same morphology is set as True (see Global rules [1]), (non-ventricular) tachycardia is set as True (see Global rules [5]), AV block is set as False to prevent unforeseeable generated registries (see Global rules [4]), and all CCs are marked as ectopics (see Per-cycle rules [3]). Secondly, for all cardiac cycles, P waves and PQ segments are removed. Thirdly, in addition to the changes mentioned in Global rules [4], the ST segment is substituted by a random walk of fixed length ($ST = CumulativeSum(\mathcal{N}(\mu, \sigma, size = \text{ST\_LENGTH}))$, where $\mu = 0.15$, $\sigma = 1$ and $\text{ST\_LENGTH} = RandomInteger(2, 15))$ and smoothed by convolving the signal with a Hamming window.
7. **(True/False; p $<$ 10%) Has sinus arrest?**
   If set to True, the P, PQ, QRS, ST and T segments are skipped for $\text{N\_ARREST} = RandomInteger(1, 3)$ CCs, effectively generating CCs that are composed solely of TP segments (which serve as rest segments). To increase variability, the onset of the arrest is also randomly generated: $\text{ON\_ARREST} = RandomInteger(2, 5)$.
8. **(True/False; p $<$ 20%) Has segment elevations?**
   If set to True, segment elevations are generated for all segments. These elevations ELEV_i are modelled as increases or decreases in amplitude (10% for the PQ, ST and TP segments and 20% for the P, QRS and T segments), so that $\text{ELEVATIONS}_i = \sum_{j=0}^{j=i} \text{ELEV}_j$ for all generated segments i. The cumulative elevations are added for all existing segments in the final registry by generating a noise vector of size N: $\text{NOISE} = Concatenate(LinSpace(\text{ELEVATIONS}_i, \text{ELEVATIONS}_{i+1}, size = \text{N\_SEGMENT}_i)) \; \forall i \in [0, K]$. The resulting elevation profile is then smoothed and added back to the generated signal: $\text{SIGNAL}Smoothing(\text{NOISE})$. It is important to notice that the elevations need not be the same if Global rules [1] is active.

1.2 Per-cycle rules

Per-cycle rules are those which affect single cardiac cycles (CC) within a generated registry. All rules below could potentially be active at the same time. For this project, the following per-cycle rules were designed:

1. **(True/False; PQ: p $<$ 15%; ST: p $<$ 25%; TP: p $<$ 25%) Merge PQ/ST/TP segment?**
   If set to True, this instruction allows partially overlapping different segments, effectively skipping the electrical pause segments (e.g. merge the P and QRS waves, skipping the PQ segment). Firstly, if the neighbouring segments exist (i.e., both the P and QRS waves are present when merging the PQ segment), the segment is marked to be skipped and the neighbouring segments are marked to be merged. Secondly, the two

segments are overlapped. For this purpose, an array MERGED is created with a size N_MERGED $=$ SEGMENT$_1$ $+$ PTG_SEGM$_2$ $\cdot$ SEGMENT$_2$, where PTG_SEGM$_2$ is [75-95]$\%$ in the case of the PQ and ST segments and [50-80]$\%$ in the case of the TP segment, as generated by uniform distributions. Then, the first array is added in MERGED$[0$ $:$ N_SEGMENT$_1]$SEGMENT$_1$ and the second array is added in MERGED$[-$N_SEGMENT$_2$ $:$ $end]$SEGMENT$_2$. Finally, the sample where the final waveform accumulates an error higher than 50$\%$ is sought for (LOCATION $= ArgMax(Absolute($SEGMENT$_1$ $-$ MERGED$[0$ $:$ N_SEGMENT$_1])/Amplitude($SEGMENT$_1) > 0.5)$, so that the MERGED array is cropped into a first segment MERGED$_1$ $=$ MERGED$[0$ $:$ LOCATION$]$ and a second segment MERGED$_2$ $=$ MERGED$[$LOCATION $:$ $end]$. This way, the ground truth of the P, QRS and T waves is altered to accommodate large changes in its waveform, which would correspond to the next wave starting.

2. **(True/False; p $<$ 10%) Has an U wave?**
   If set to True, the CC is set to have an U wave. Given the lack of annotated ground truth with respect to U waves, these were modelled as T waves with smaller amplitude (5-15$\%$ of the amplitude of the T wave). The U waves are placed as extensions of the T waves, merging both waves with an 5-25$\%$ overlap on the T wave offset, similarly to the process explained in the Per-cycle rules [1].
3. **(True/False; p $<$ 10%) Has a ventricular ectopic?**
   If set to True, the CC is marked to contain a ventricular ectopic, which was modelled as follows. Firstly, the CC's QRS and T segments are redrawn from the segment pool (for the cases where the Global rule [1] is active), and the probability of having a U wave is increased by 50$\%$. Secondly, the P and PQ segments are skipped for this CC. Thirdly, the amplitudes of the QRS and T waves are re-drawn until they are valued above a certain threshold (THR_ECTOPIC $= 0.1$). Fourthly, the QRS segment is interpolated to a width of QRS_WIDTH $= RandomInteger(30, 70)$ samples (12-28 ms) and the T wave is aligned to oppose the direction of the QRS wave. It is important to notice that naturally long QRS complexes (defined as longer than 0.16 ms, as a rule of thumb by looking at the QRS length histogram of the database) are marked as ventricular ectopics due to not having access to beat type classification. This should be improved in the future.
4. **(True/False; p $<$ 20% for every segment independently) Interpolate to a different length?**
   If set to True, each segment of the CC is interpolated (linear interpolation) to a new size in the range [85-115$\%$] of the original segment length. This allows for extended variability in the segment pool.
5. **(True/False; p $<$ 25% for the P, QRS and T segments independently) Apply mixup?**
   If set to True, mixup is applied [1■] to the P, QRS and T segments given a certain probability. Mixup involves the application of linear combination of data samples ($\hat{y} = \lambda y_1 + (1 - \lambda)y_2$, where $\lambda \sim Beta(\alpha, \beta)$, $\alpha = 25$ and $\beta = 5$). The selected values for α and β allowed obtaining mixup samples that more closely resembled y_1. For matching samples y_1 and y_2, which can potentially contain a different number of samples, y_2 was interpolated to the number of samples of y_1. Moreover, to avoid gross mismatches, if the signed maxima of y_1 and y_2 differed, the polarity of y_2 would be inverted.

2 TRAINING AND VALIDATION CURVES FOR THE ORIGINAL AND SYNTHETIC DATASETS.

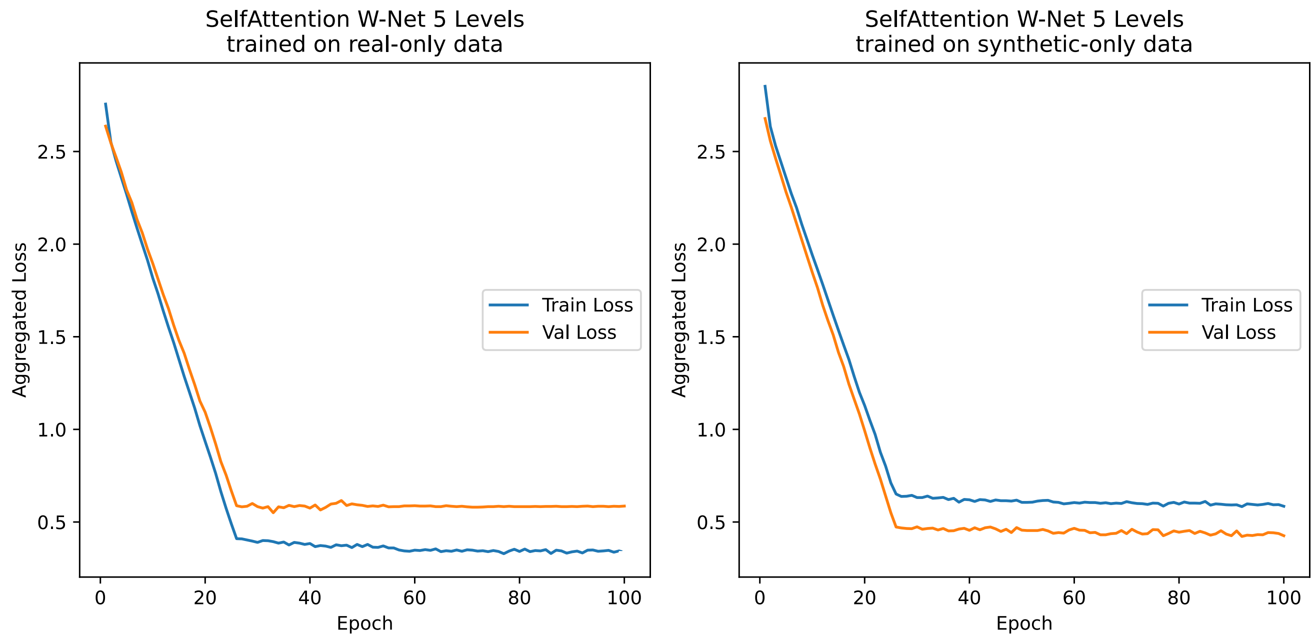

Figure S1. Training (blue) and validation (orange) losses for the original and synthetic datasets in the best performing model, a W-Net with 5 levels and self-attention.

3 EXAMPLES OF PREDICTIONS WITH THE BEST PERFORMING MODEL

In this section, a set of examples of predictions in private datasets of patients with different pathologies are represented to give an overview of the algorithm's performance.

3.1 Patients with Long QT syndrome

Predictions on a private dataset of patients diagnosed with long QT syndrome.

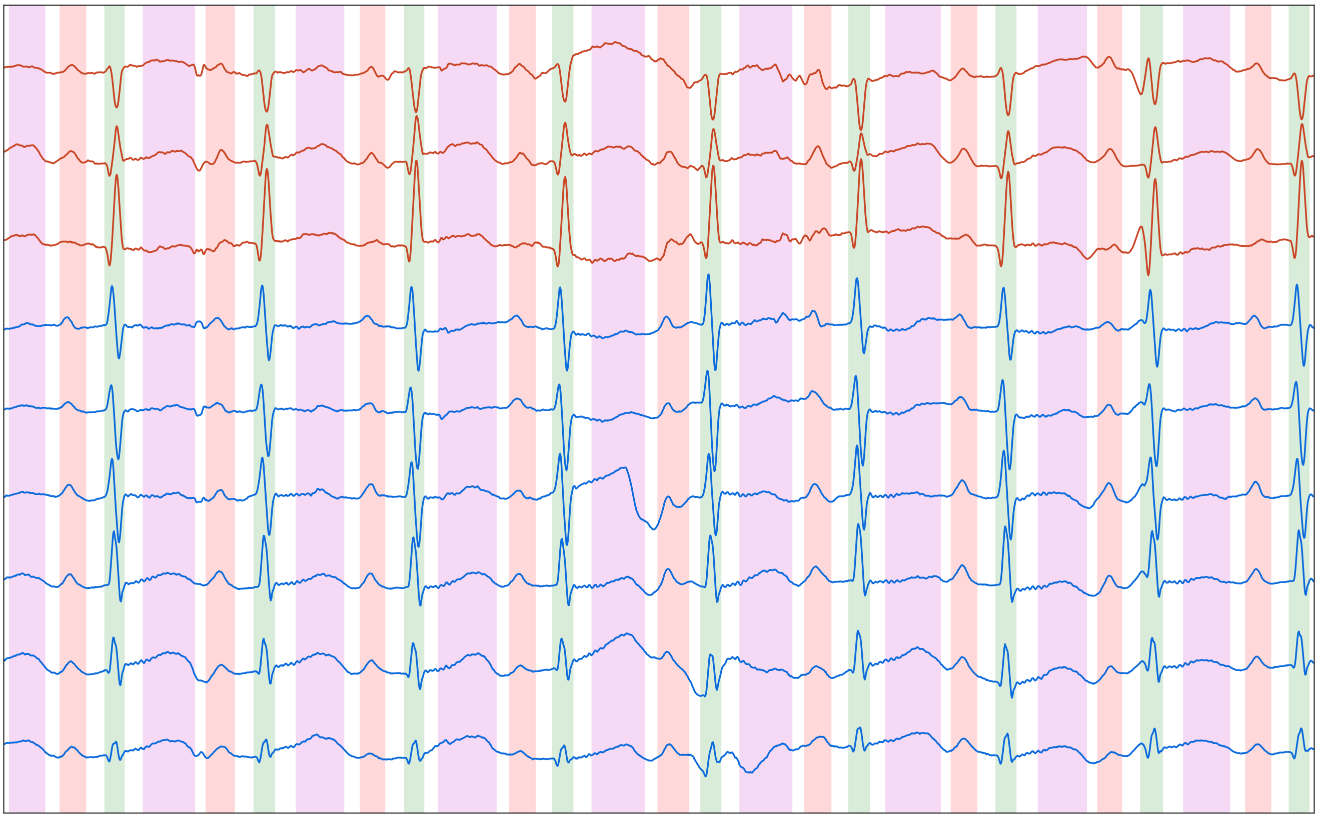

Figure S2. Colour code: P wave (red), QRS wave (green) and T wave (magenta).

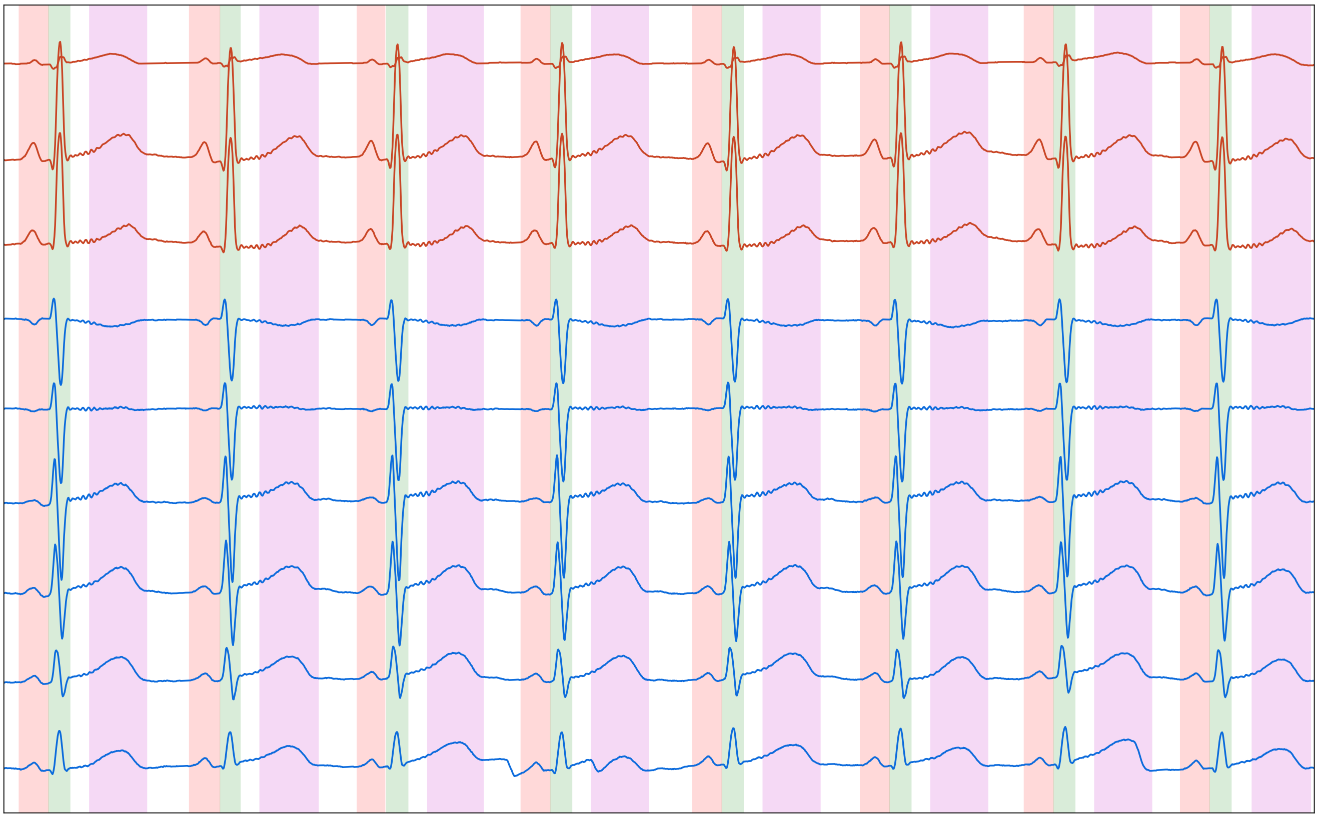

Figure S3. Colour code: P wave (red), QRS wave (green) and T wave (magenta).

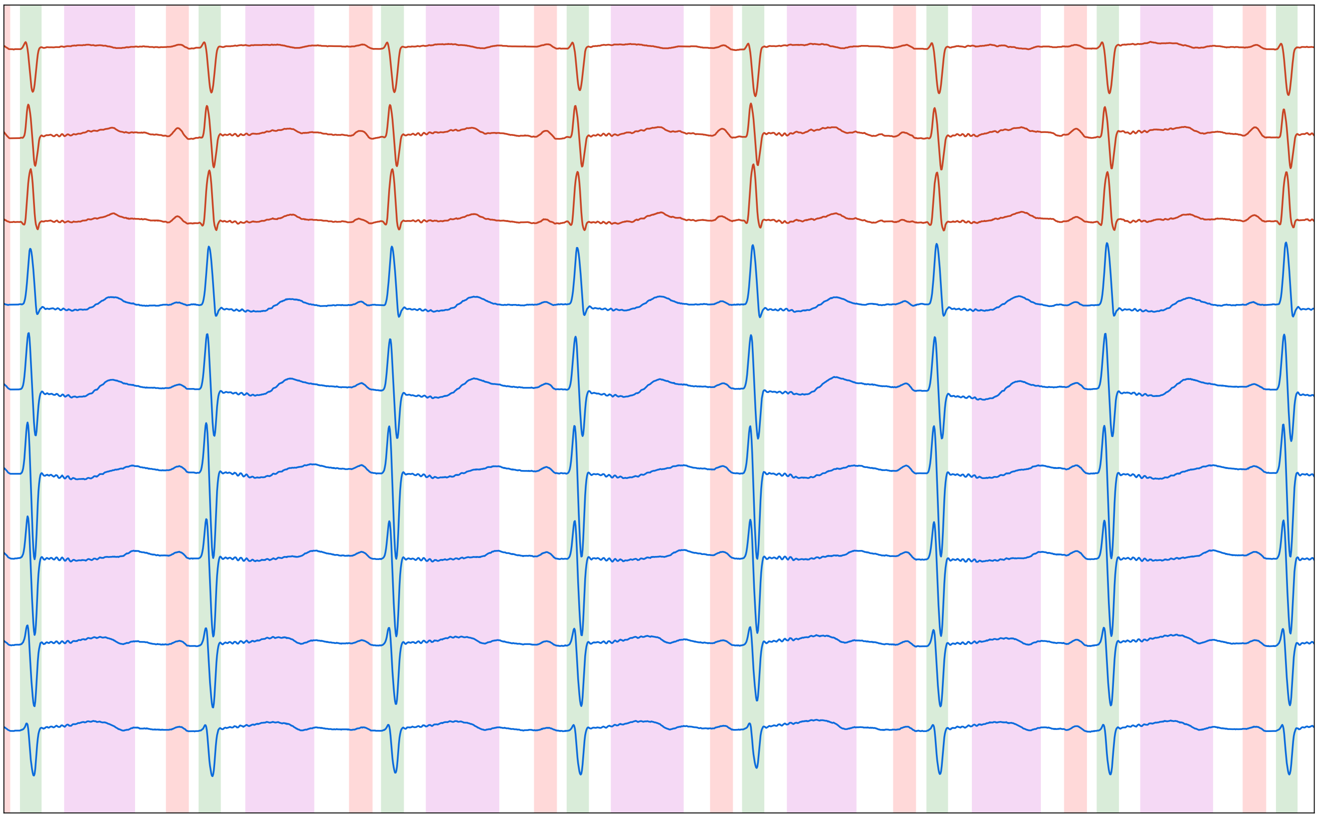

Figure S4. Colour code: P wave (red), QRS wave (green) and T wave (magenta).

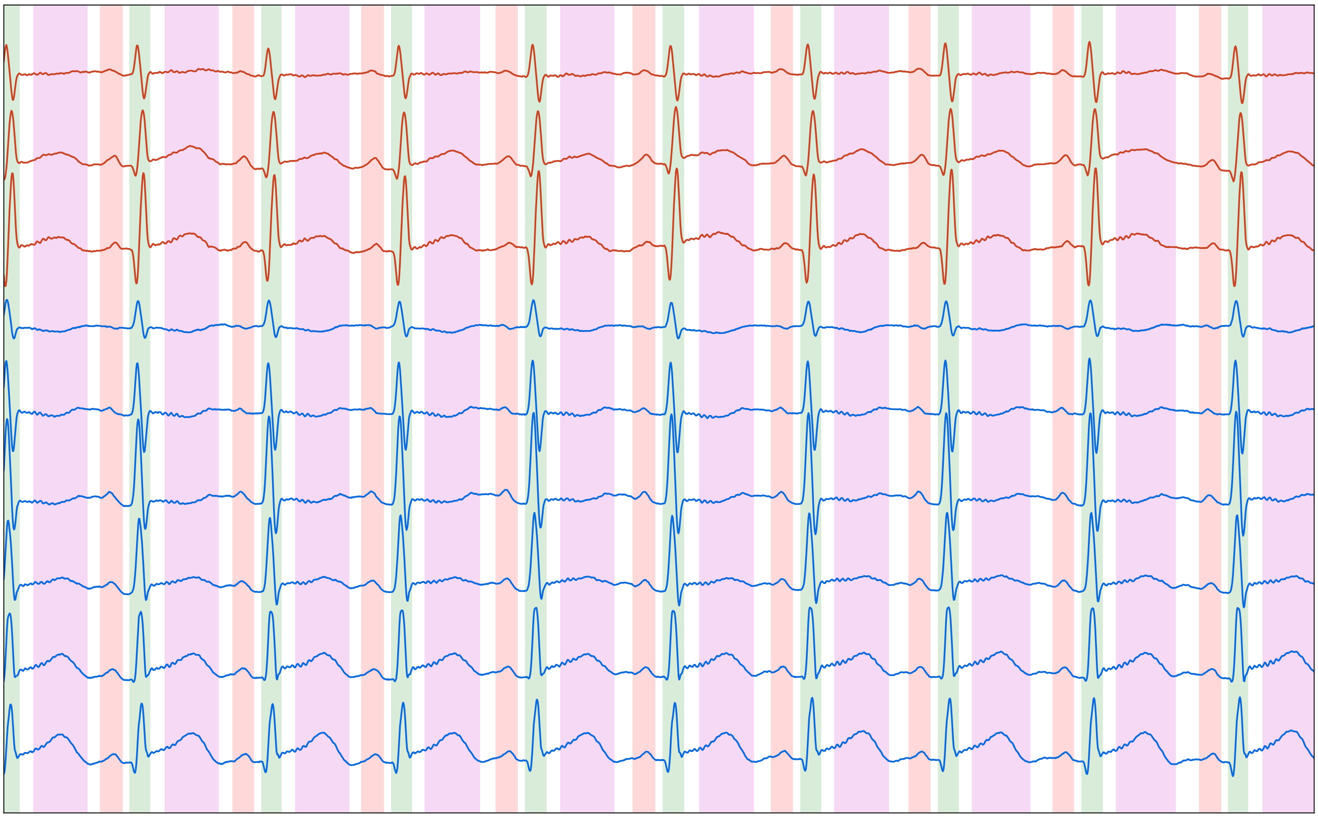

Figure S5. Colour code: P wave (red), QRS wave (green) and T wave (magenta).

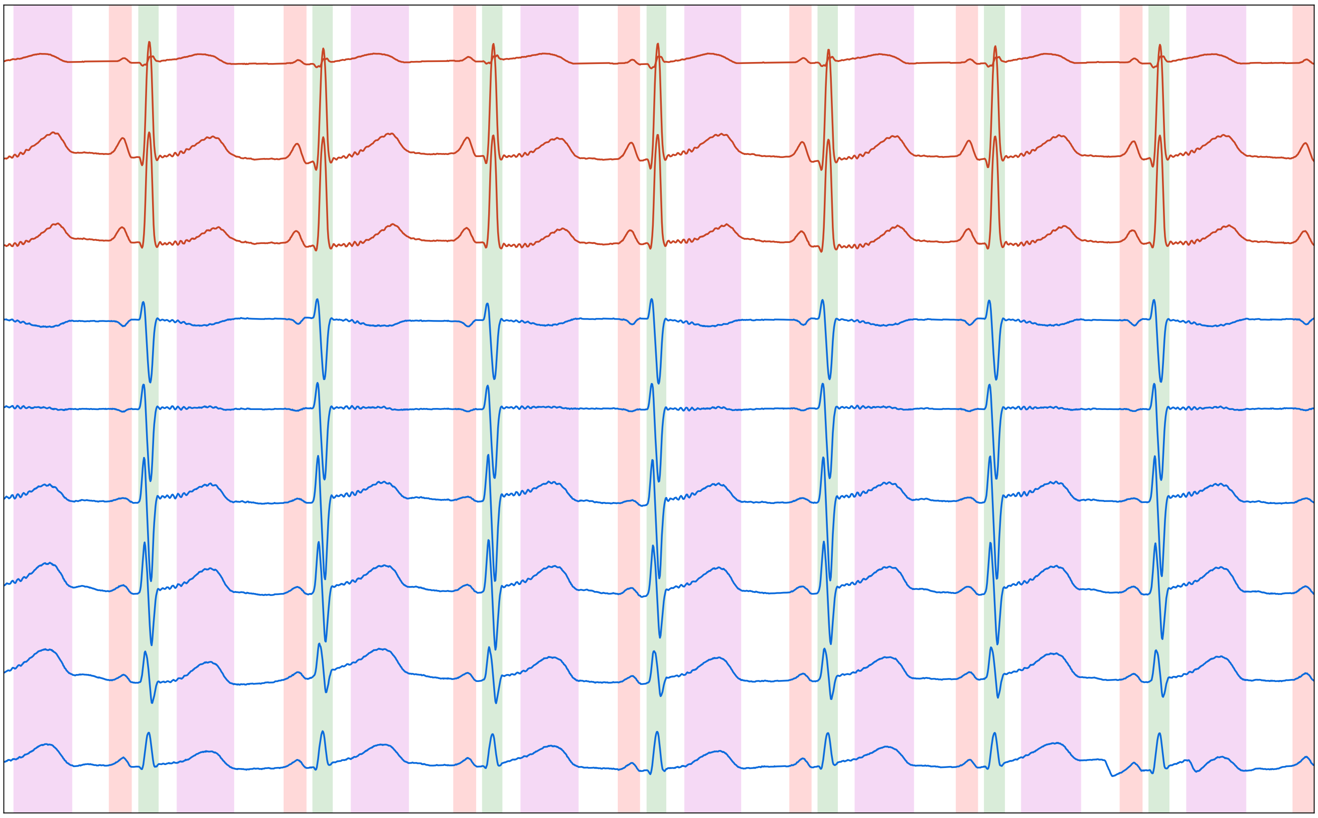

Figure S6. Colour code: P wave (red), QRS wave (green) and T wave (magenta).

3.2 Patients with intrauterine growth restriction

Predictions on a private dataset of adolescent patients who suffered intrauterine growth restriction.

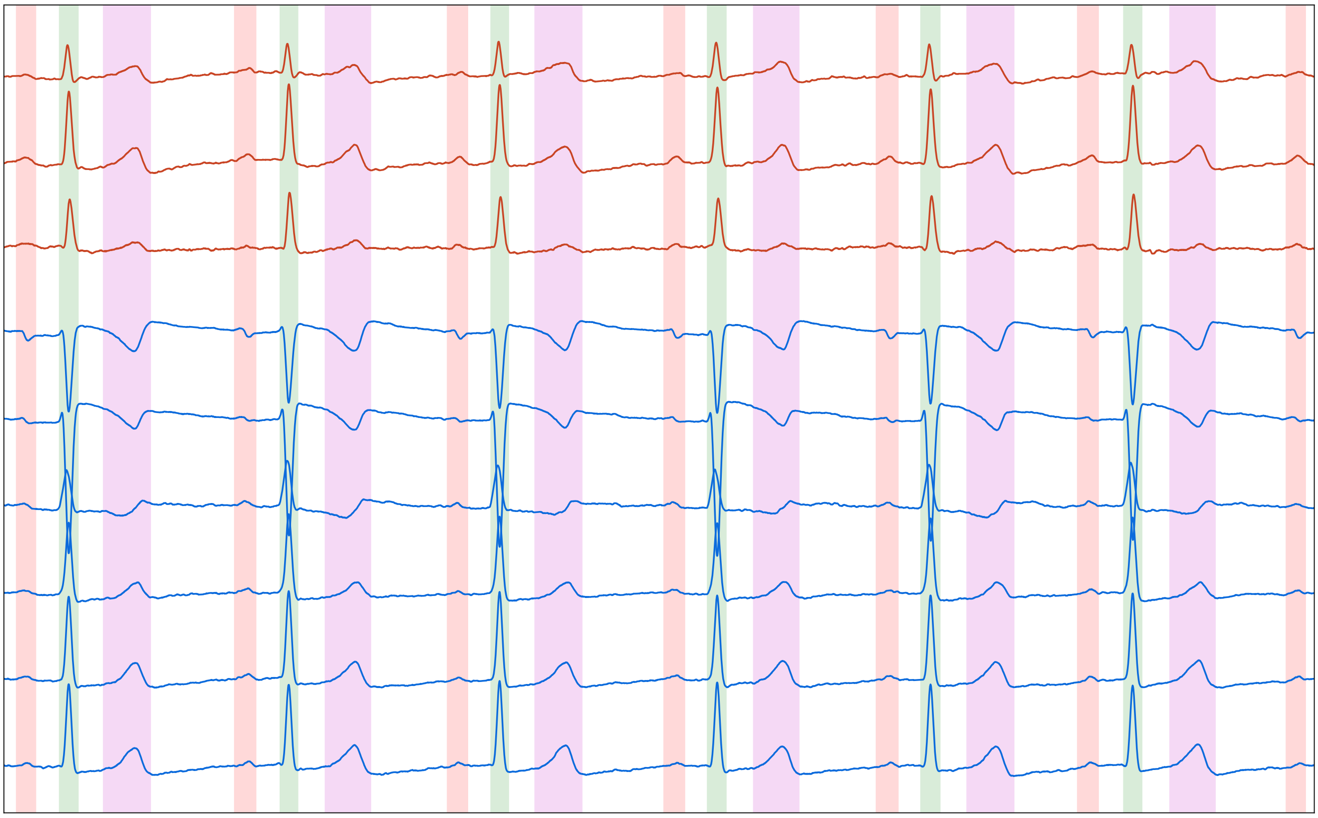

Figure S7. Colour code: P wave (red), QRS wave (green) and T wave (magenta).

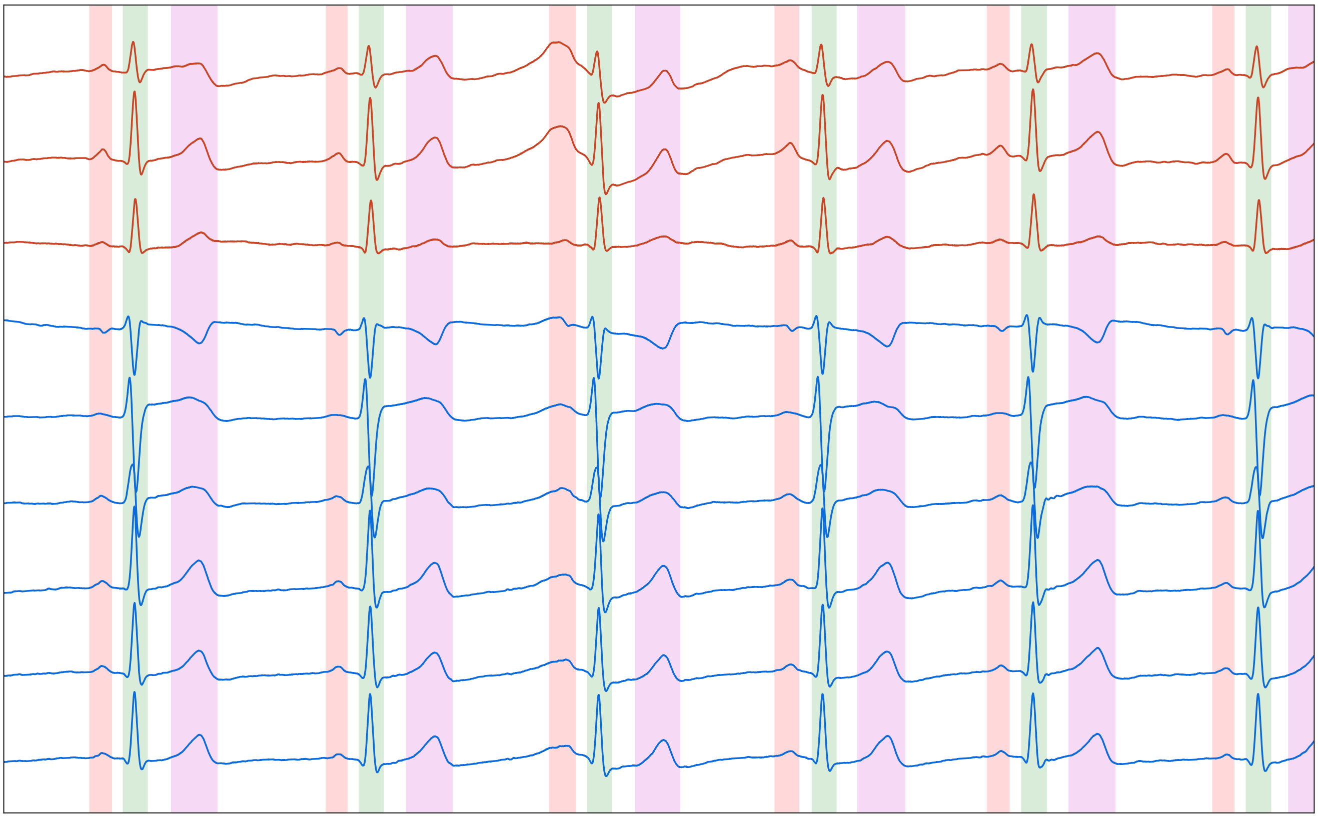

Figure S8. Colour code: P wave (red), QRS wave (green) and T wave (magenta).

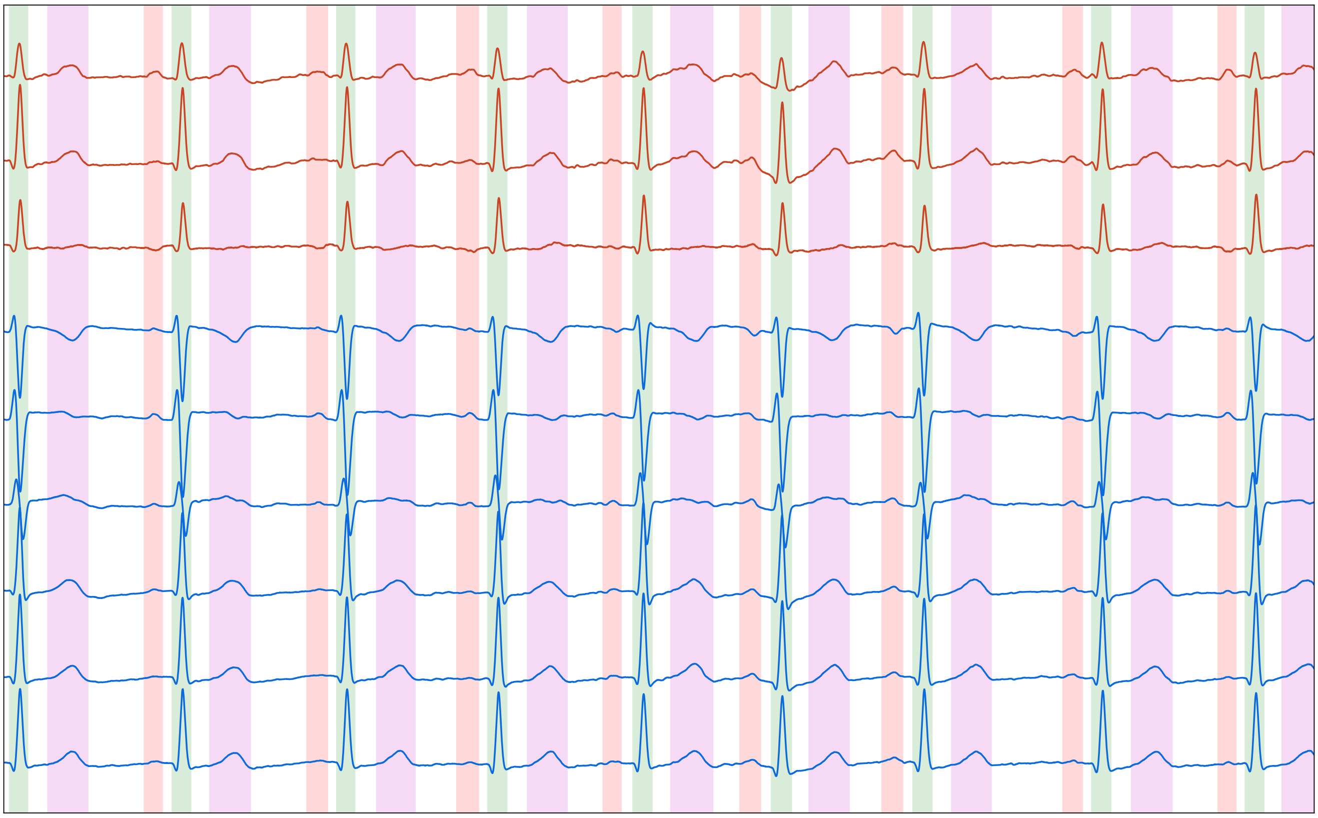

Figure S9. Colour code: P wave (red), QRS wave (green) and T wave (magenta).

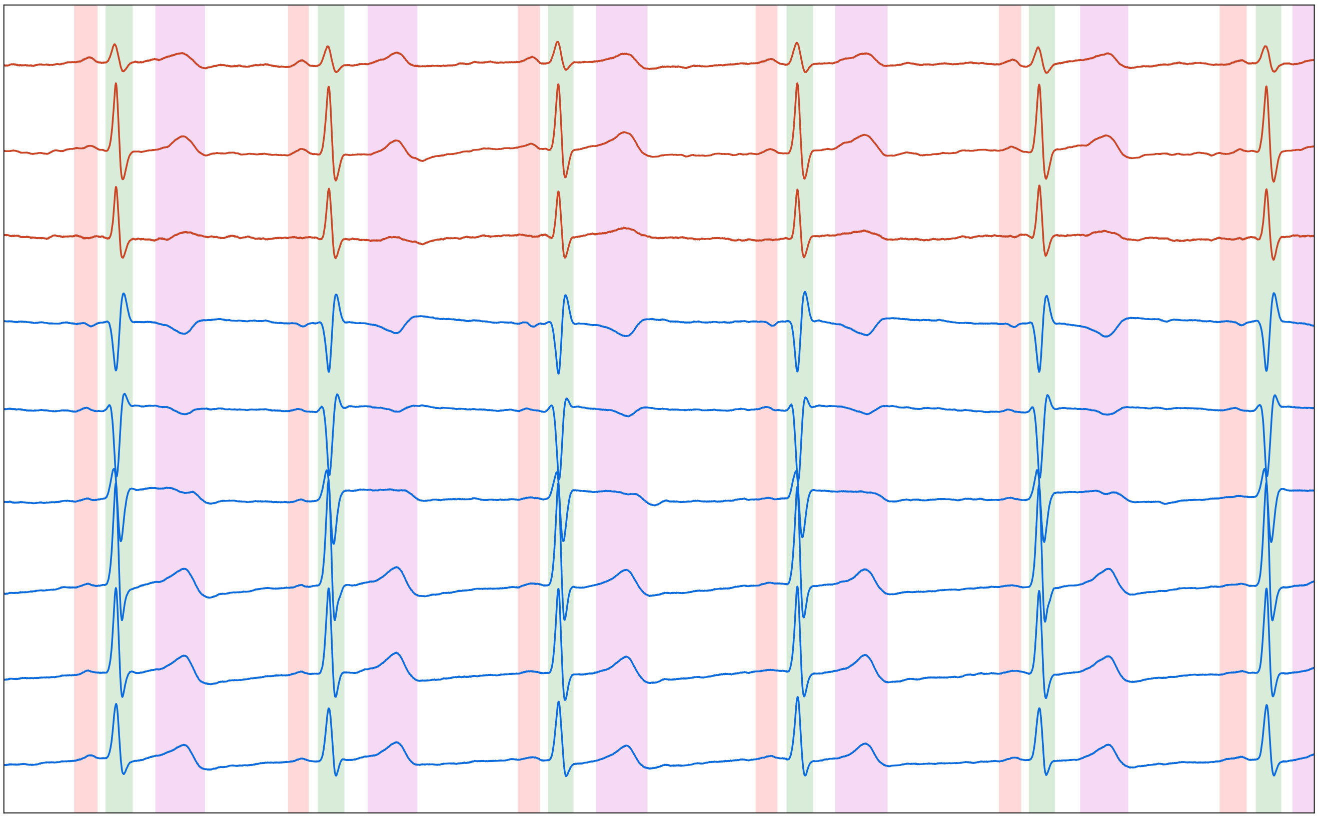

Figure S10. Colour code: P wave (red), QRS wave (green) and T wave (magenta).

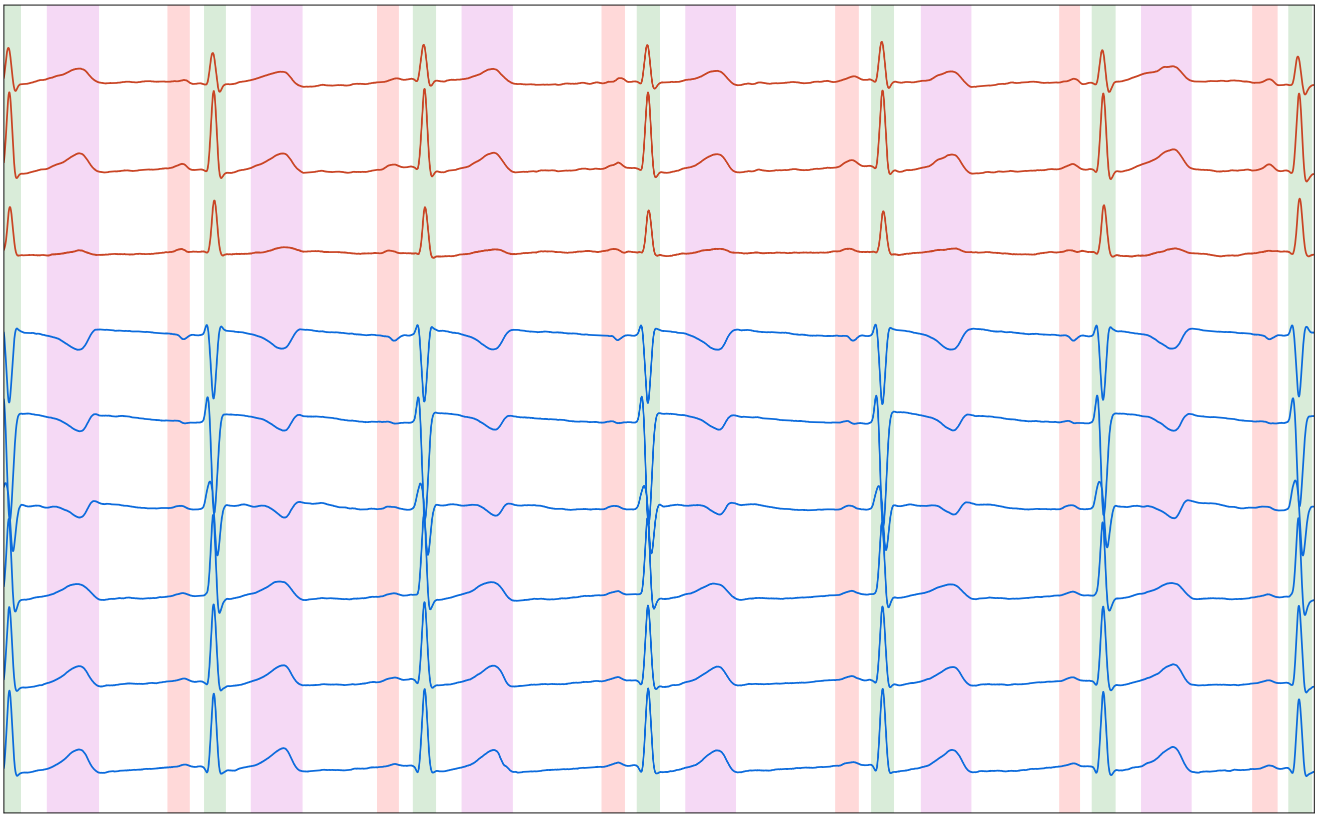

Figure S11. Colour code: P wave (red), QRS wave (green) and T wave (magenta).

3.3 Patients with hypertrophic cardiomyopathy

Predictions on a private dataset of patients diagnosed with hypertrophic cardiomyopathy.

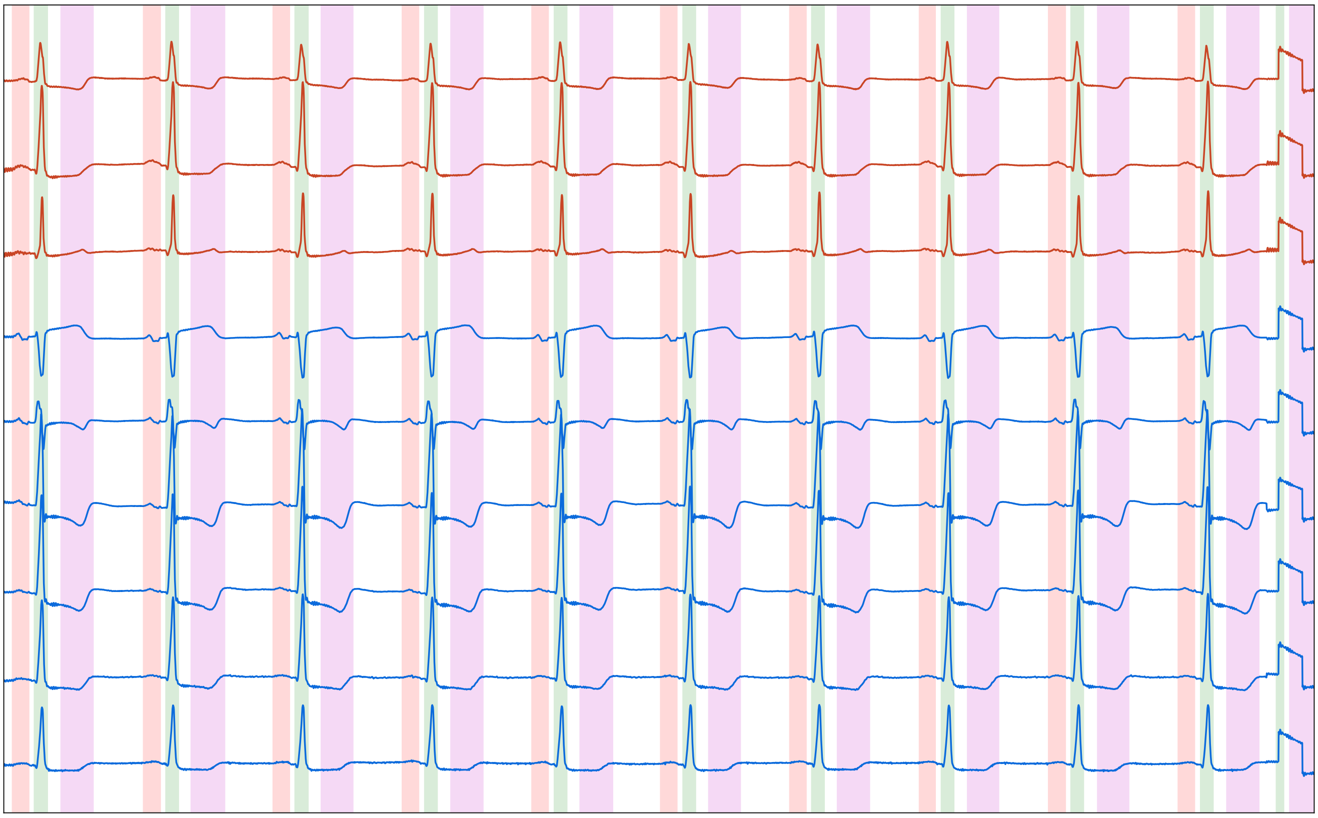

Figure S12. Colour code: P wave (red), QRS wave (green) and T wave (magenta).

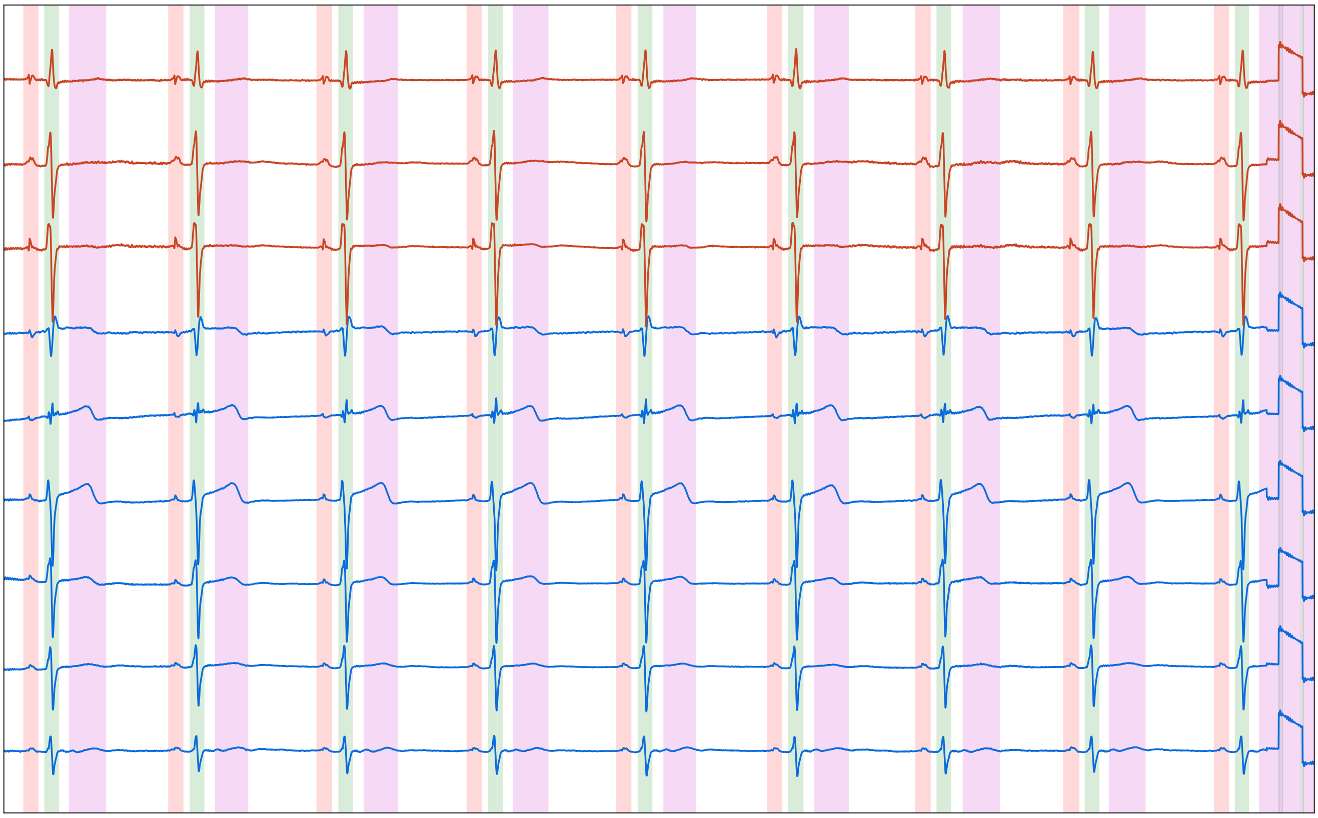

Figure S13. Colour code: P wave (red), QRS wave (green) and T wave (magenta).

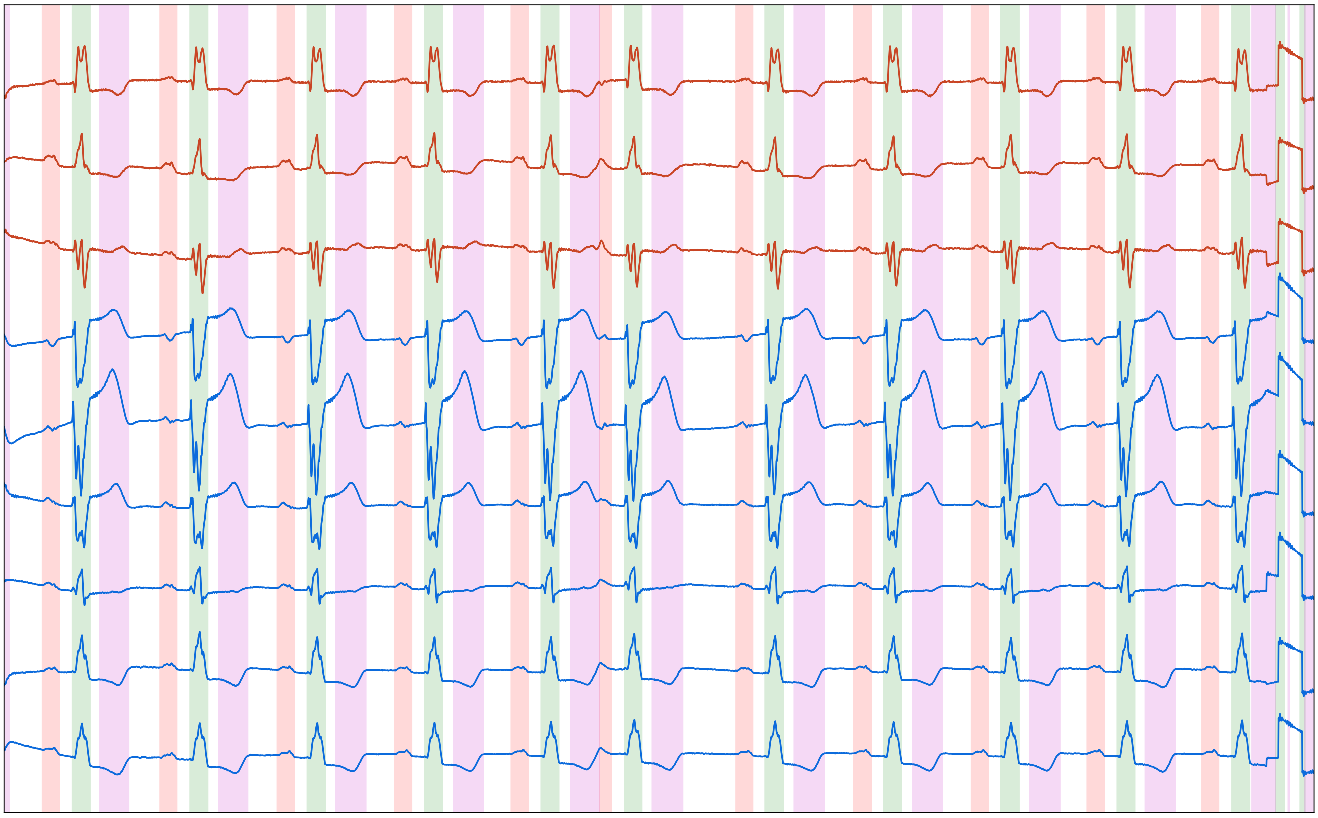

Figure S14. Colour code: P wave (red), QRS wave (green) and T wave (magenta).

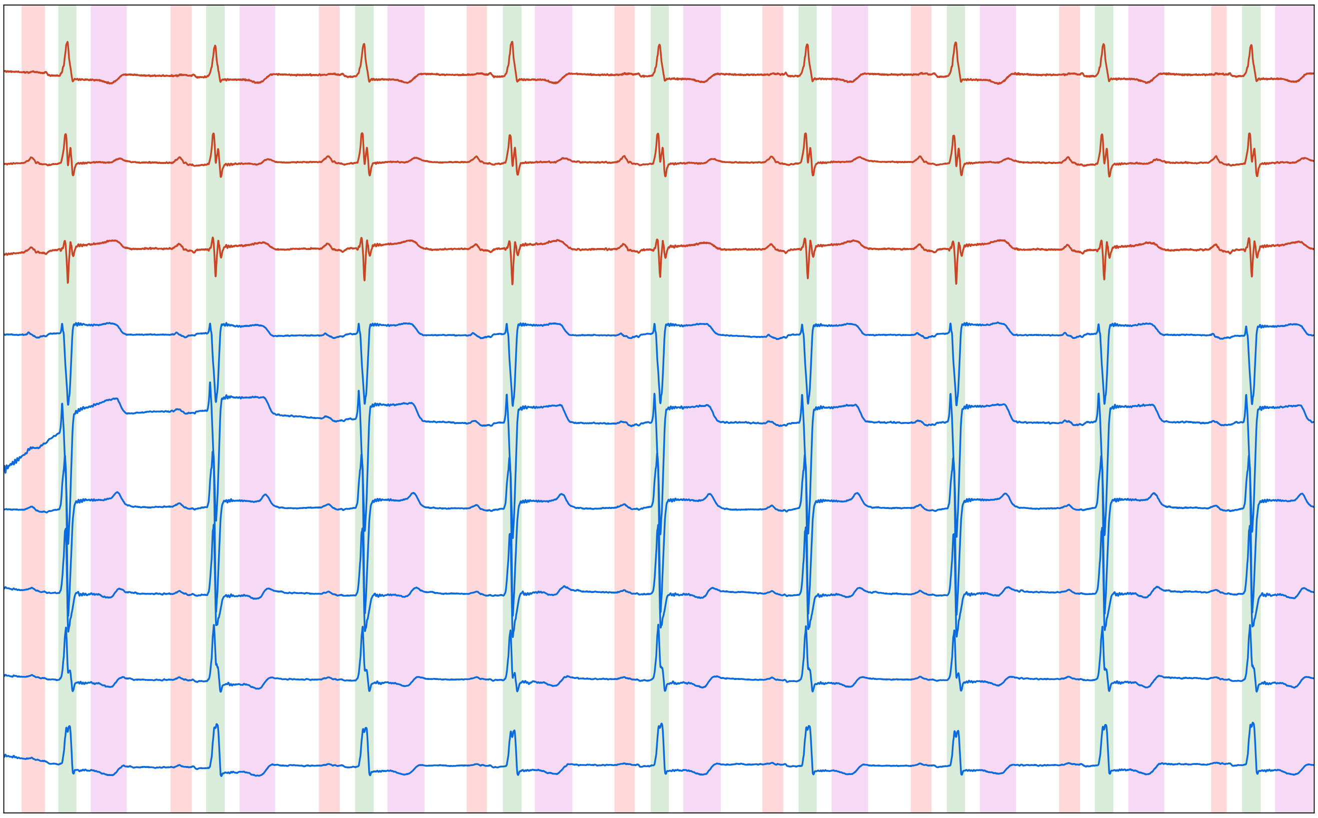

Figure S15. Colour code: P wave (red), QRS wave (green) and T wave (magenta).

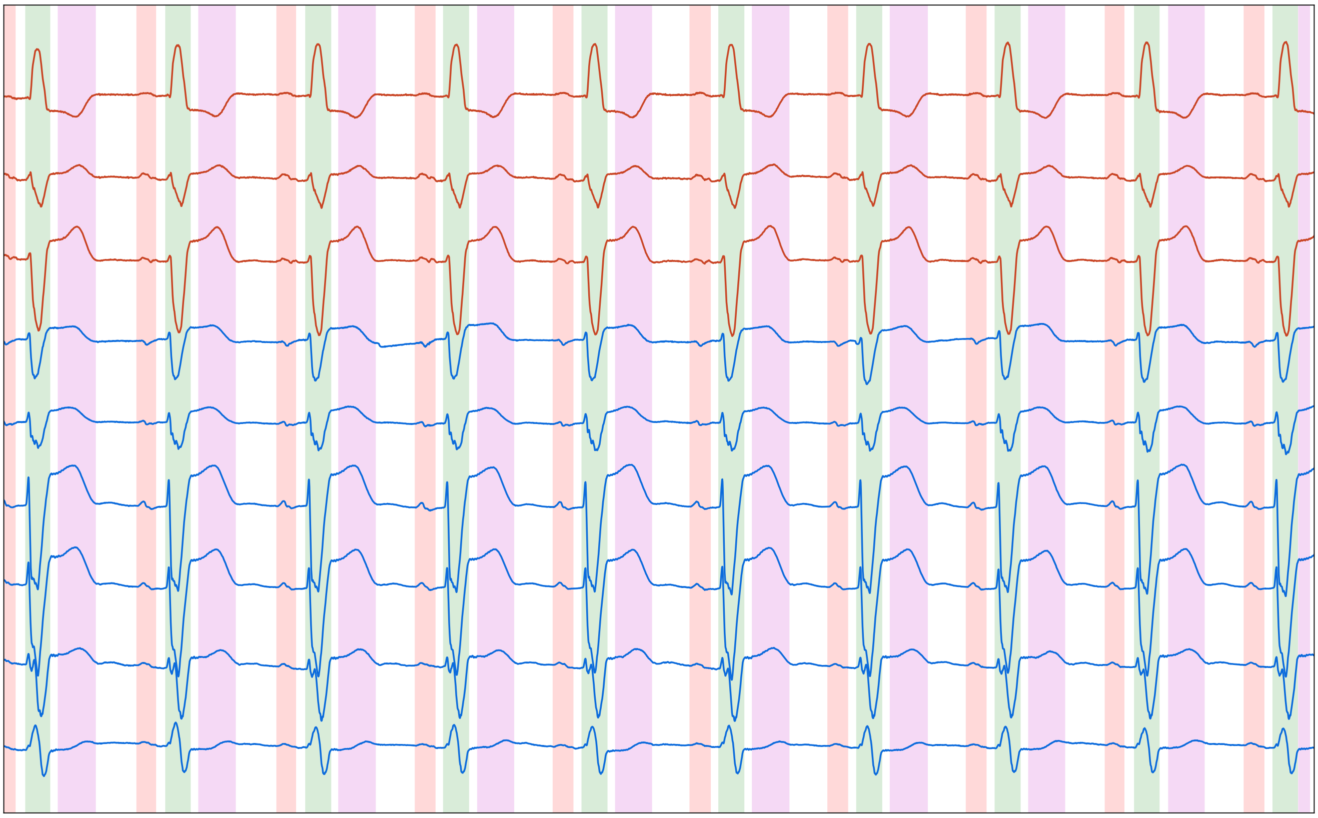

Figure S16. Colour code: P wave (red), QRS wave (green) and T wave (magenta).

3.4 Patients with Tetralogy of Fallot

Predictions on a private dataset of patients diagnosed with Tetralogy of Fallot.

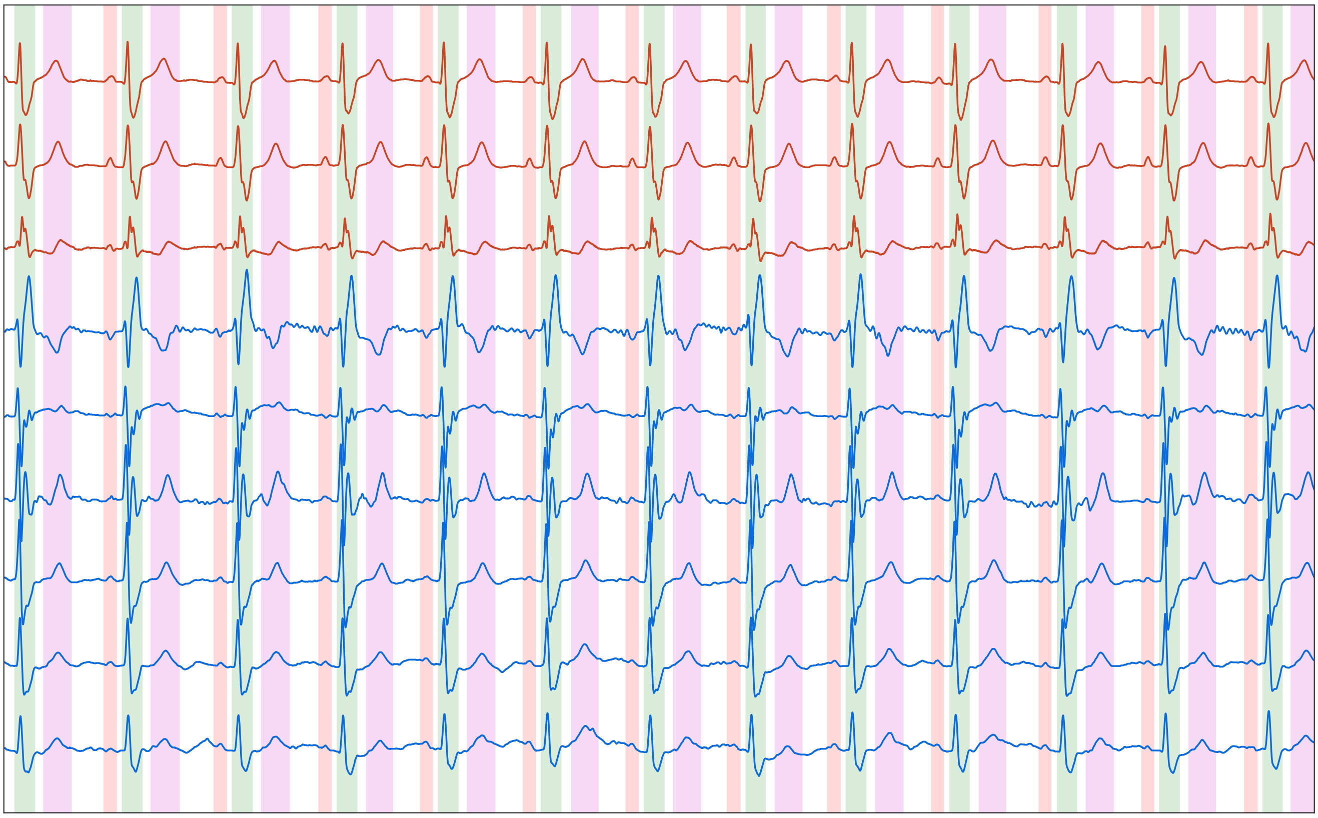

Figure S17. Colour code: P wave (red), QRS wave (green) and T wave (magenta).

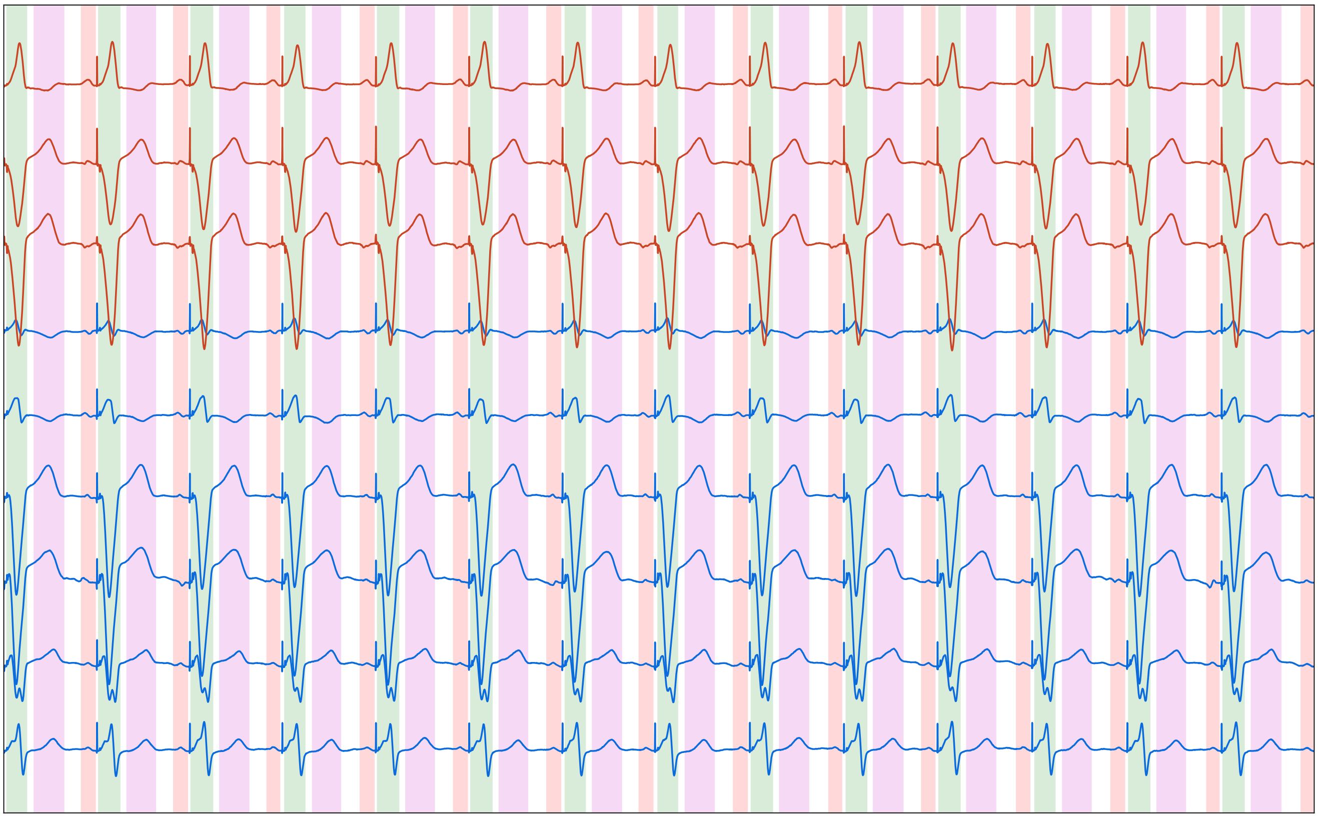

Figure S18. Colour code: P wave (red), QRS wave (green) and T wave (magenta).

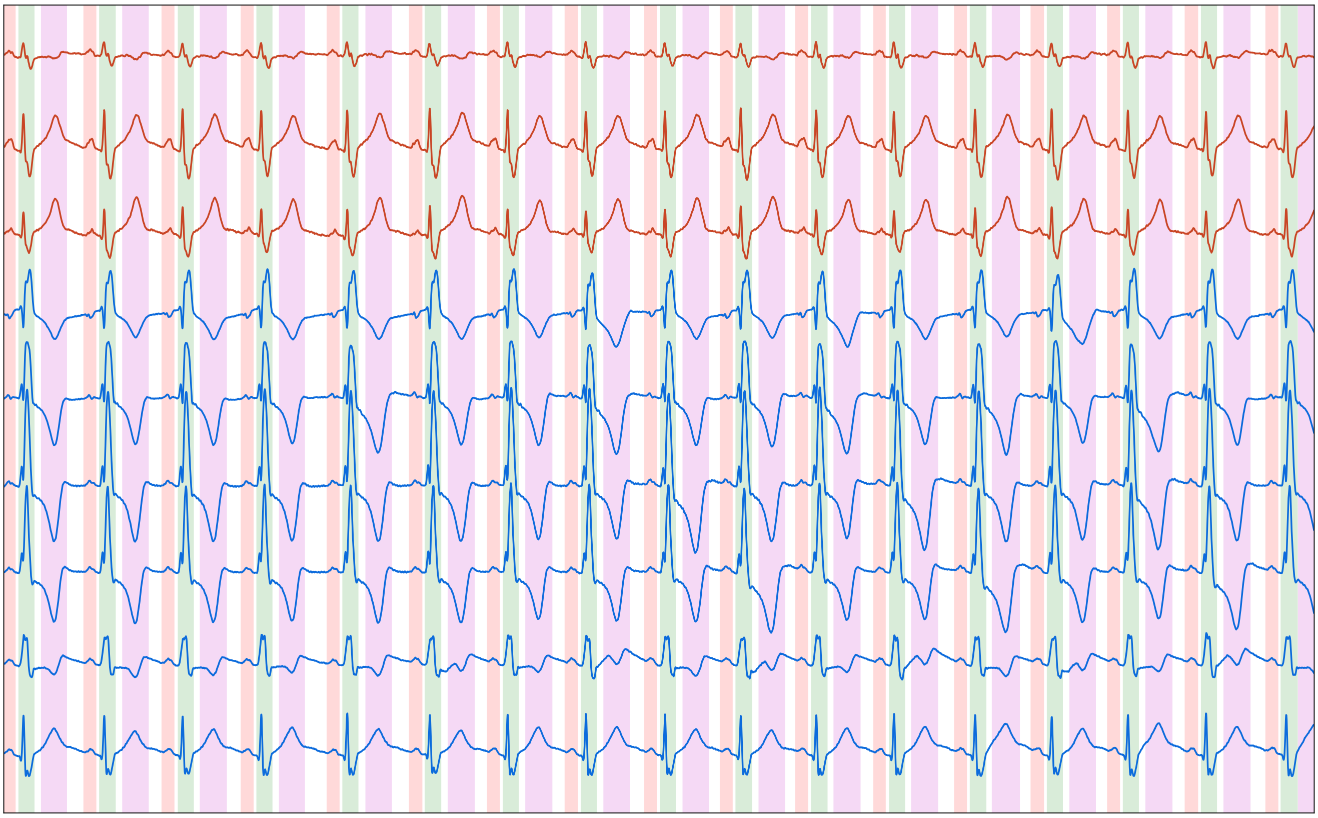

Figure S19. Colour code: P wave (red), QRS wave (green) and T wave (magenta).

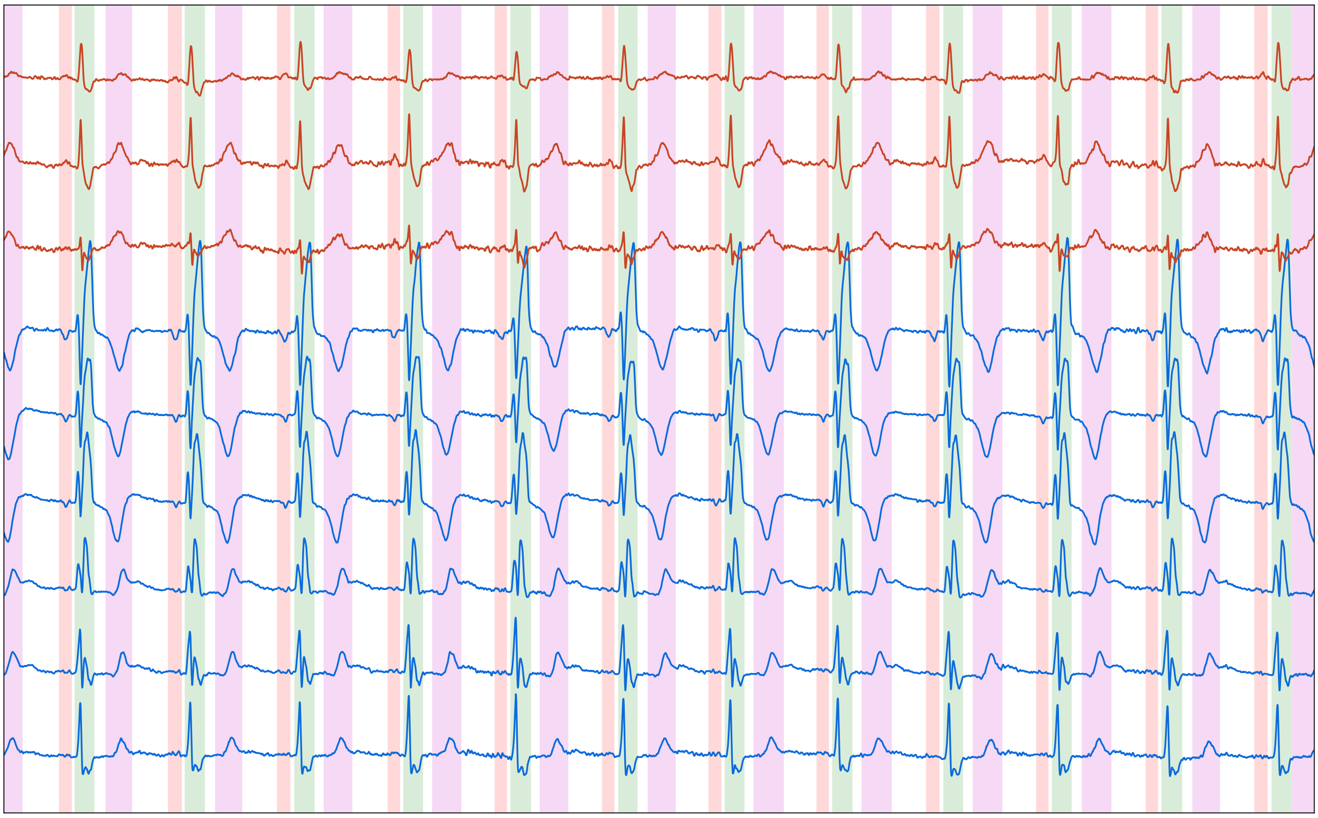

Figure S20. Colour code: P wave (red), QRS wave (green) and T wave (magenta).

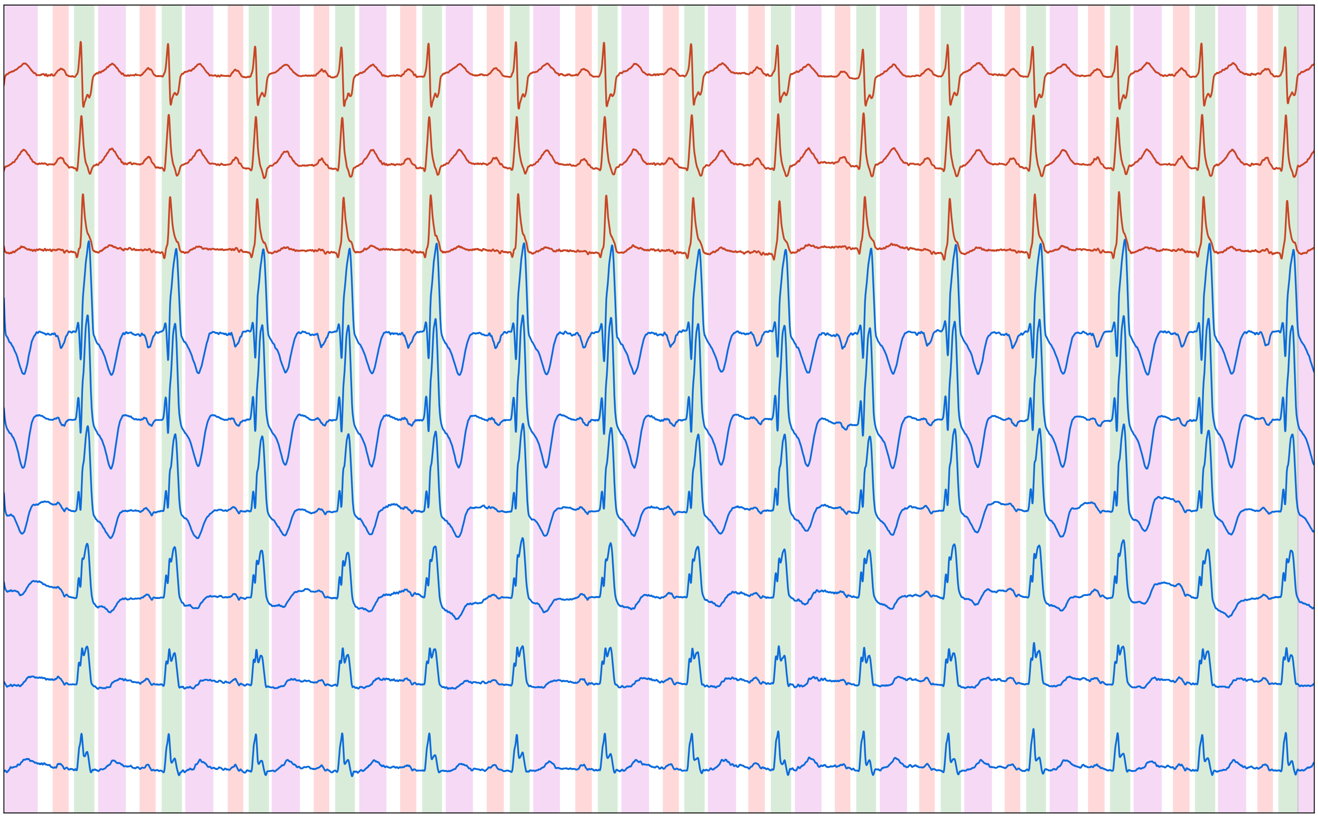

Figure S21. Colour code: P wave (red), QRS wave (green) and T wave (magenta).

3.5 Patients with Brugada syndrome

Predictions on a private dataset of patients diagnosed with Brugada syndrome.

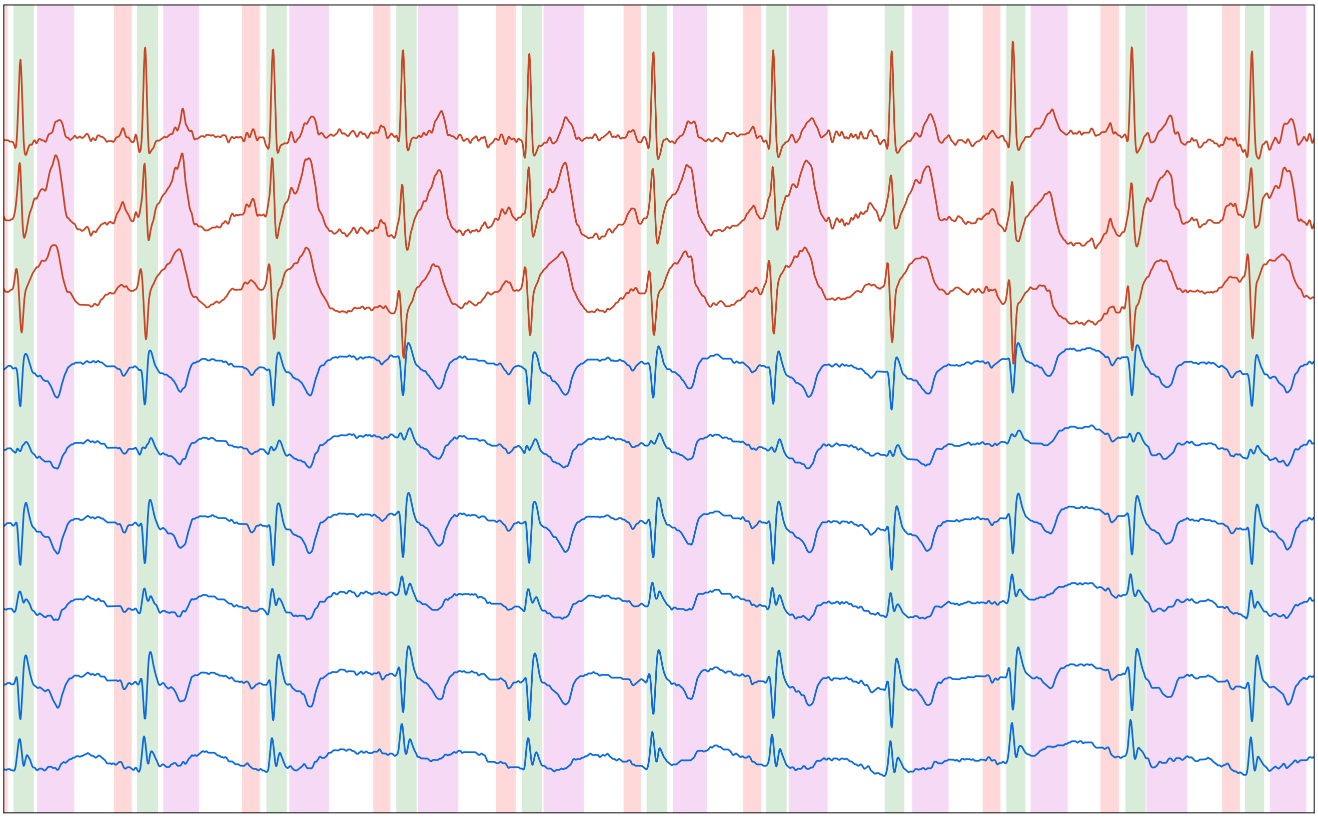

Figure S22. Colour code: P wave (red), QRS wave (green) and T wave (magenta).

REFERENCES

[1■]H Zhang, M Cisse, Y N Dauphin, and D Lopez-Paz, "mixup: Beyond Empirical Risk Minimization," in *International Conference on Learning Representations* 2018.

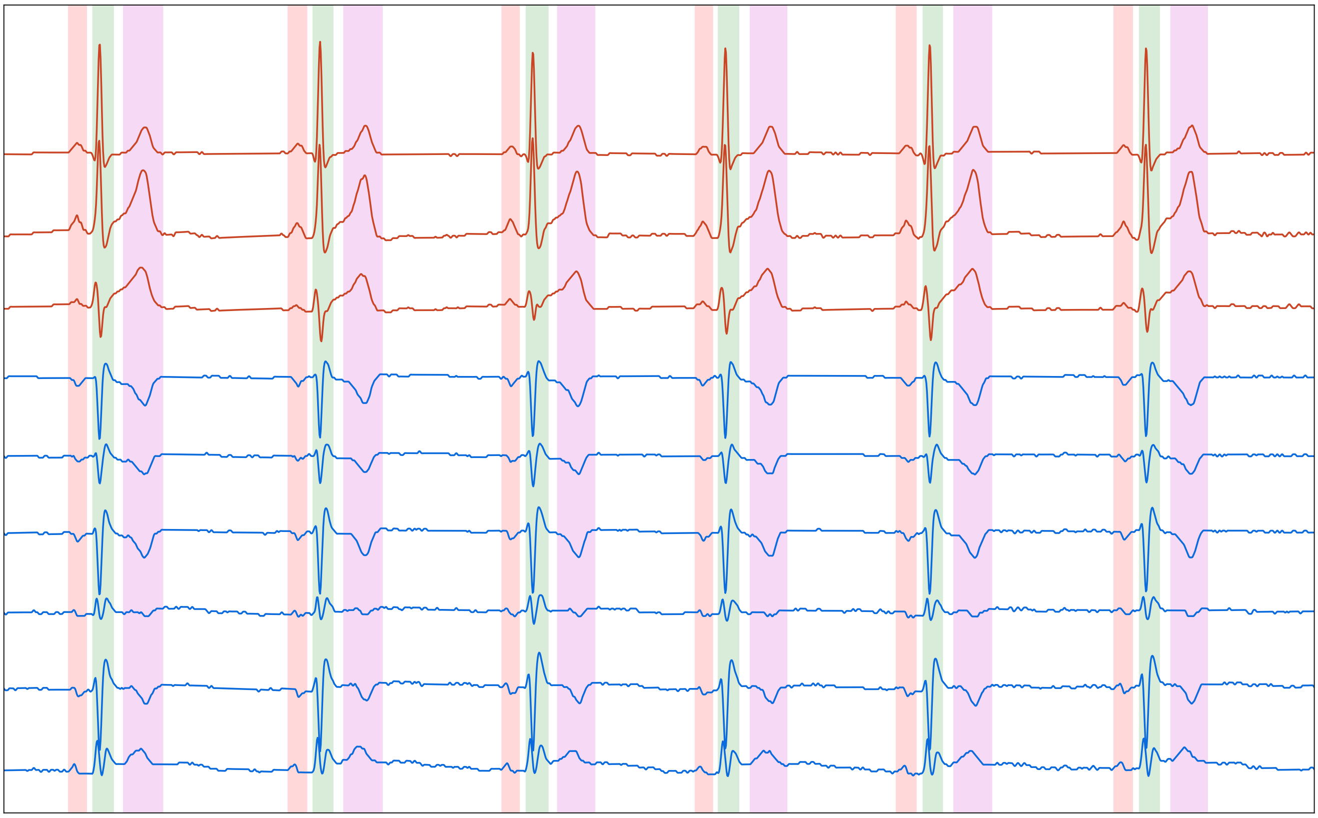

Figure S23. Colour code: P wave (red), QRS wave (green) and T wave (magenta).

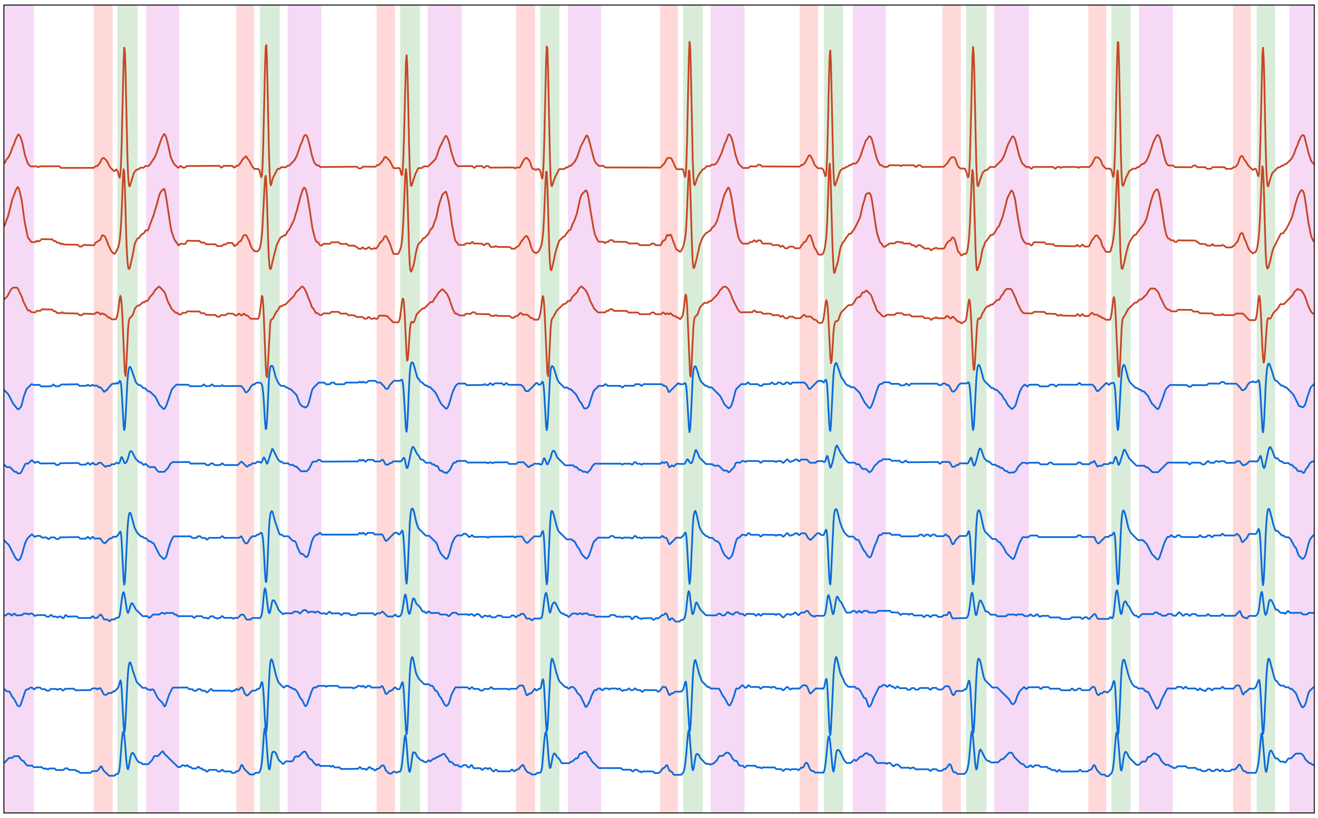

Figure S24. Colour code: P wave (red), QRS wave (green) and T wave (magenta).

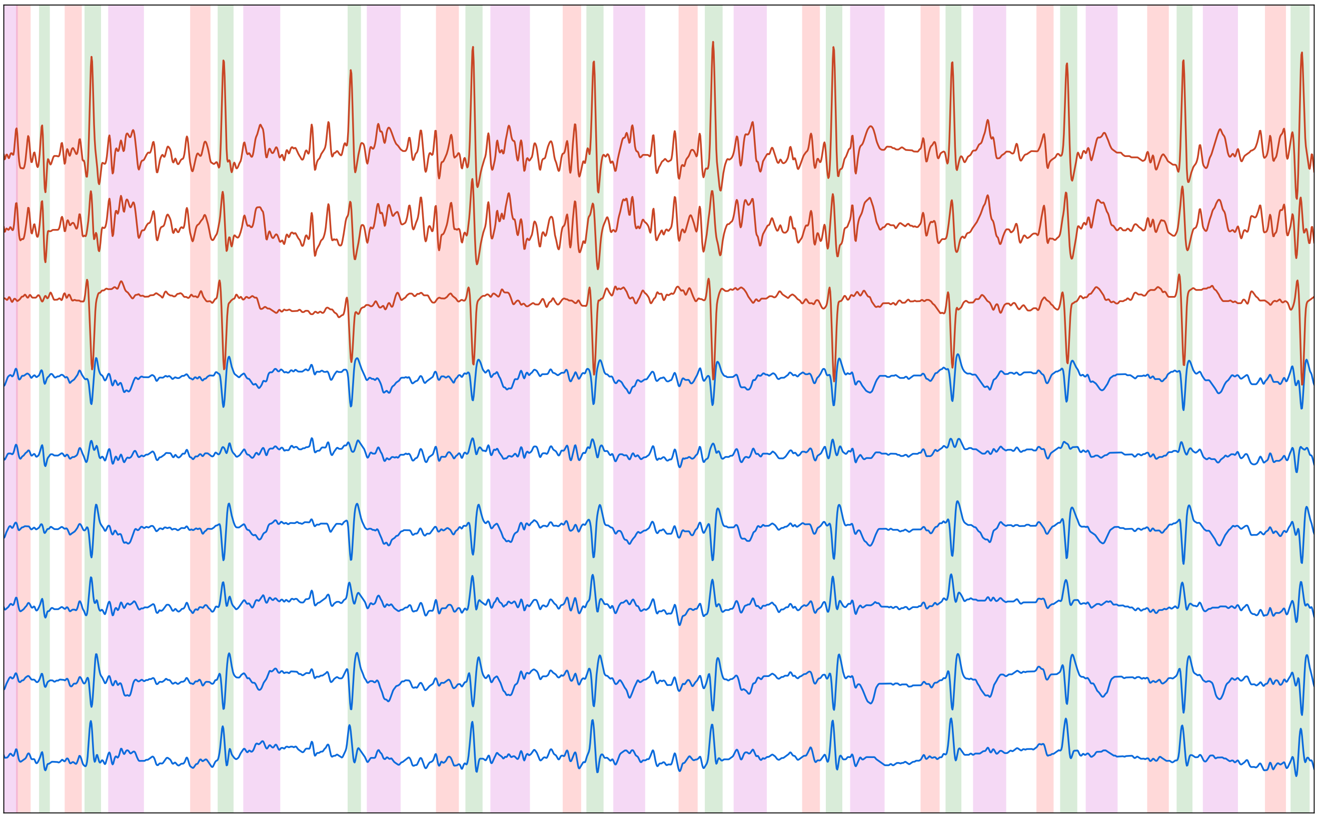

Figure S25. Colour code: P wave (red), QRS wave (green) and T wave (magenta).

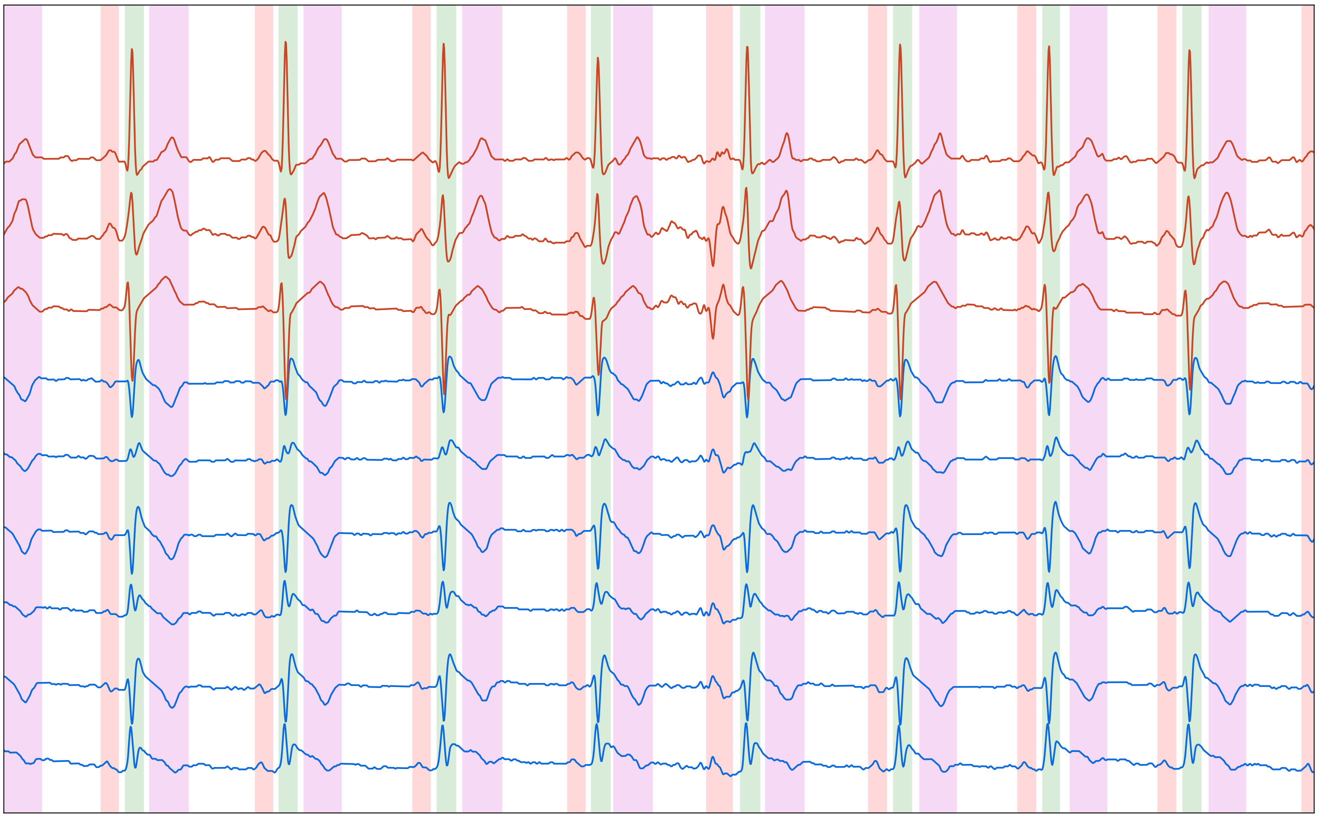

Figure S26. Colour code: P wave (red), QRS wave (green) and T wave (magenta).
