# Supplementary figures and images for: Generalising electrocardiogram detection and delineation: training convolutional neural networks with synthetic data augmentation
Source: Front Cardiovasc Med. 2024 Jul 19;11:1341786. doi: 10.3389/fcvm.2024.1341786 (PMC11294154; doi:10.3389/fcvm.2024.1341786)

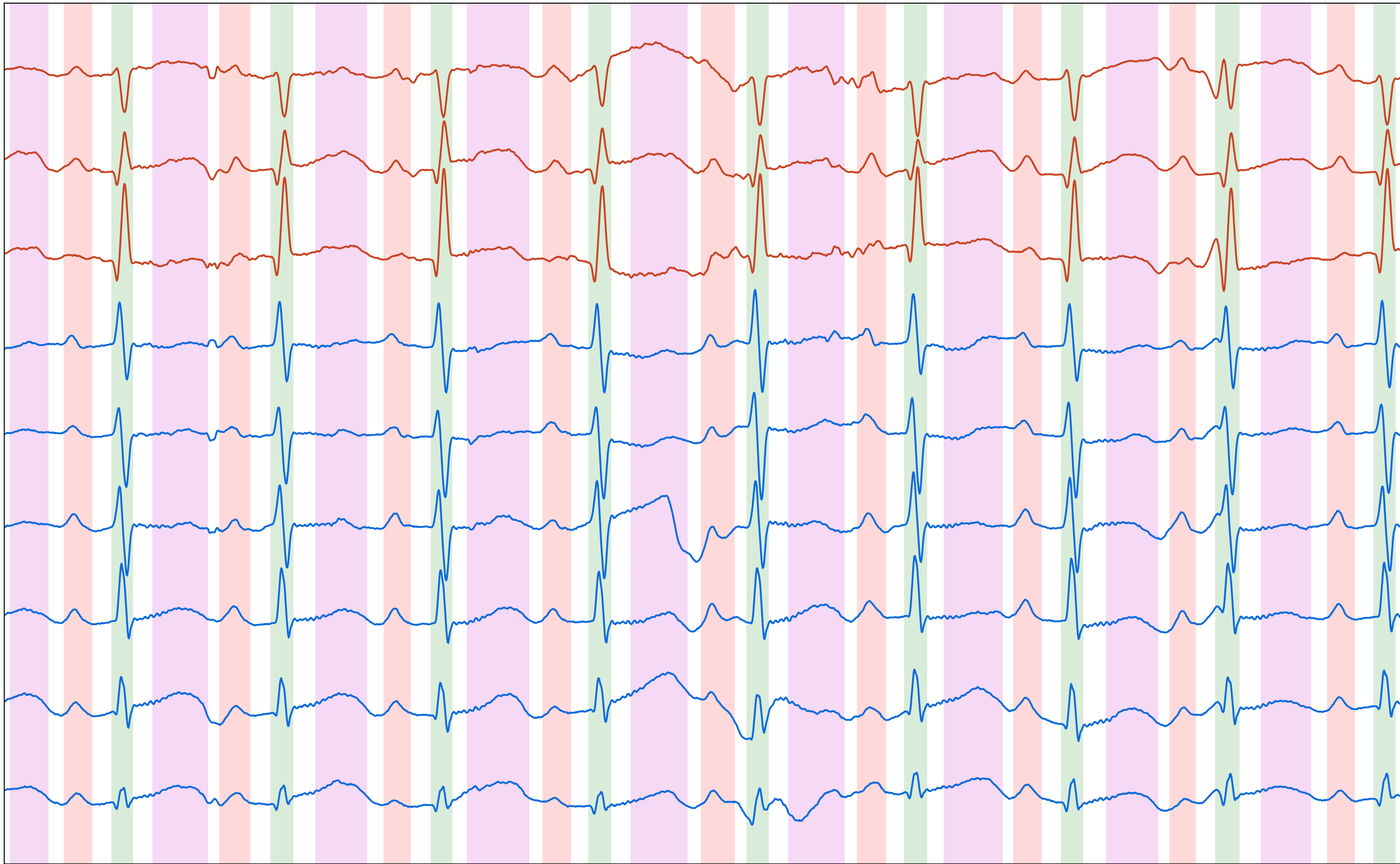

Supplement: Supplementary file 1 [file Datasheet1.zip › longqt5.pdf]

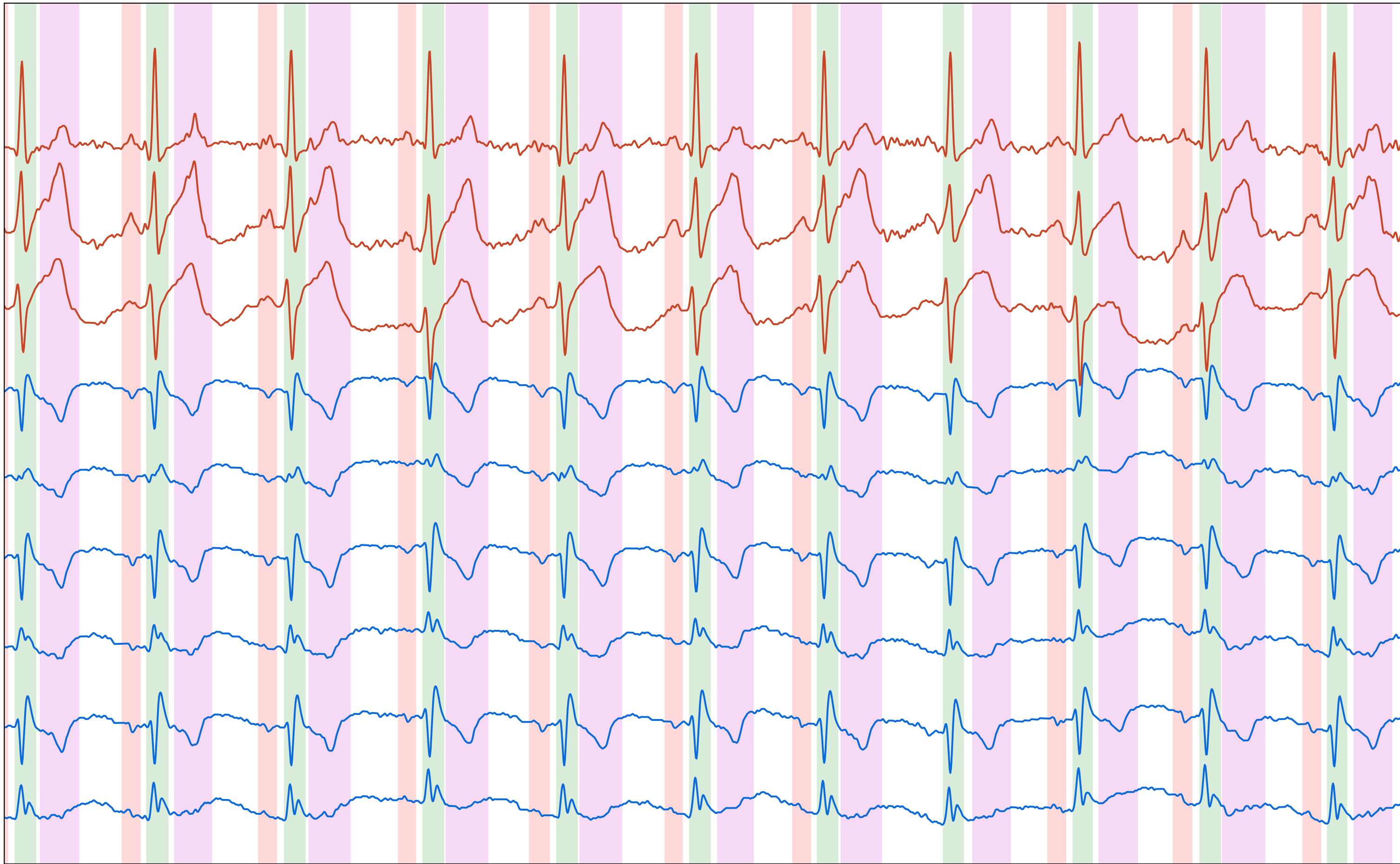

Supplement: Supplementary file 1 [file Datasheet1.zip › brugada1.pdf]

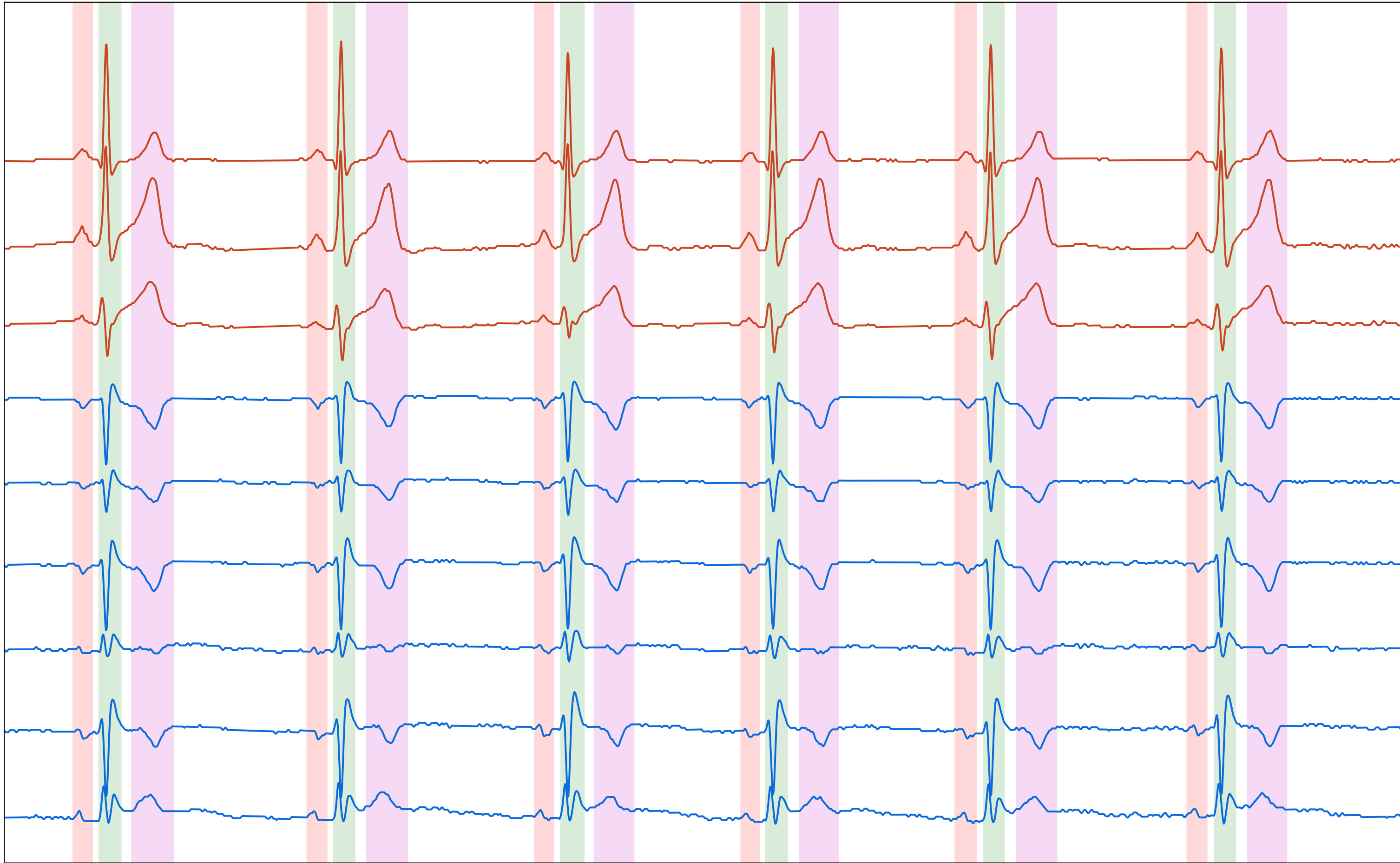

Supplement: Supplementary file 1 [file Datasheet1.zip › brugada2.pdf]

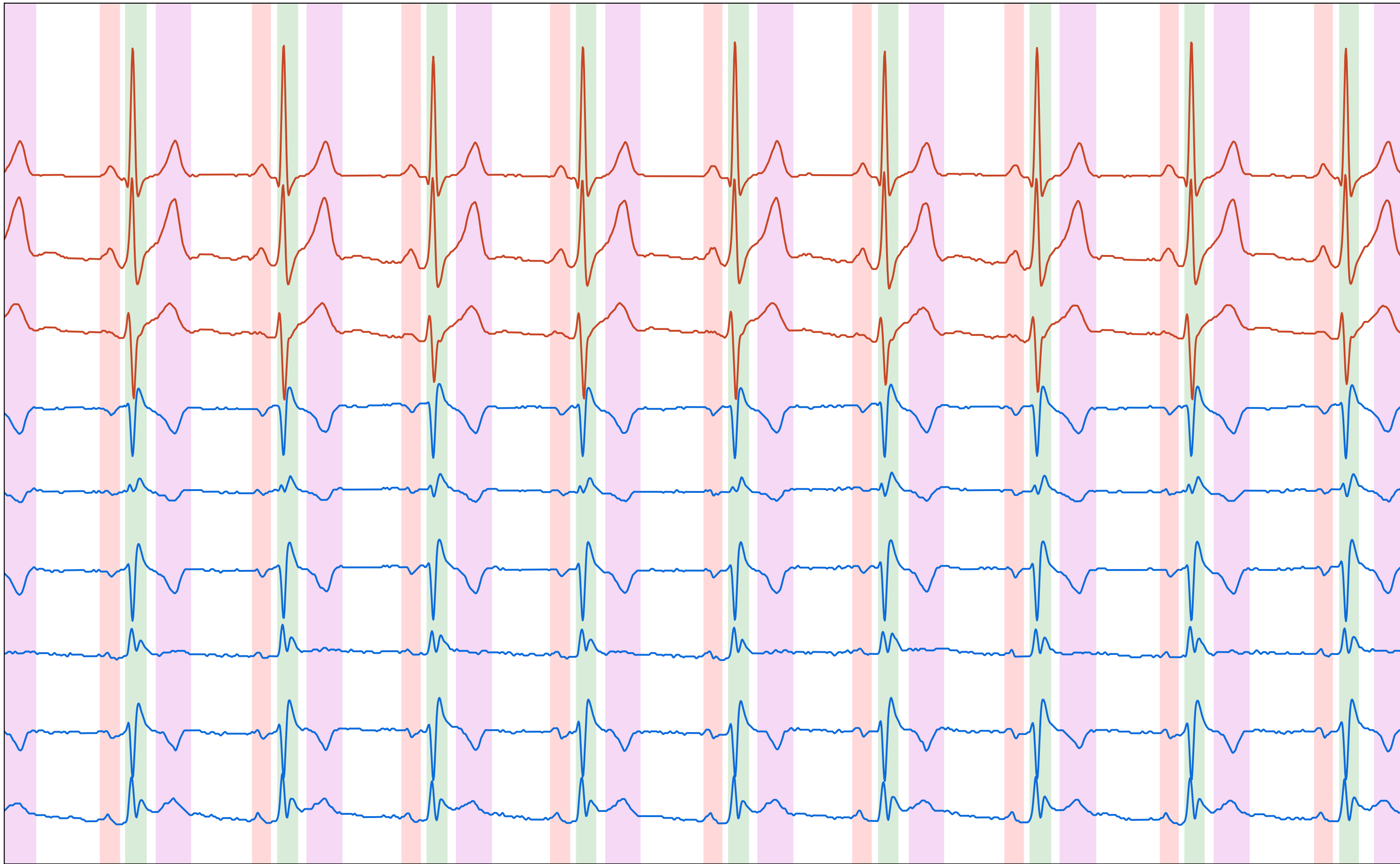

Supplement: Supplementary file 1 [file Datasheet1.zip › brugada3.pdf]

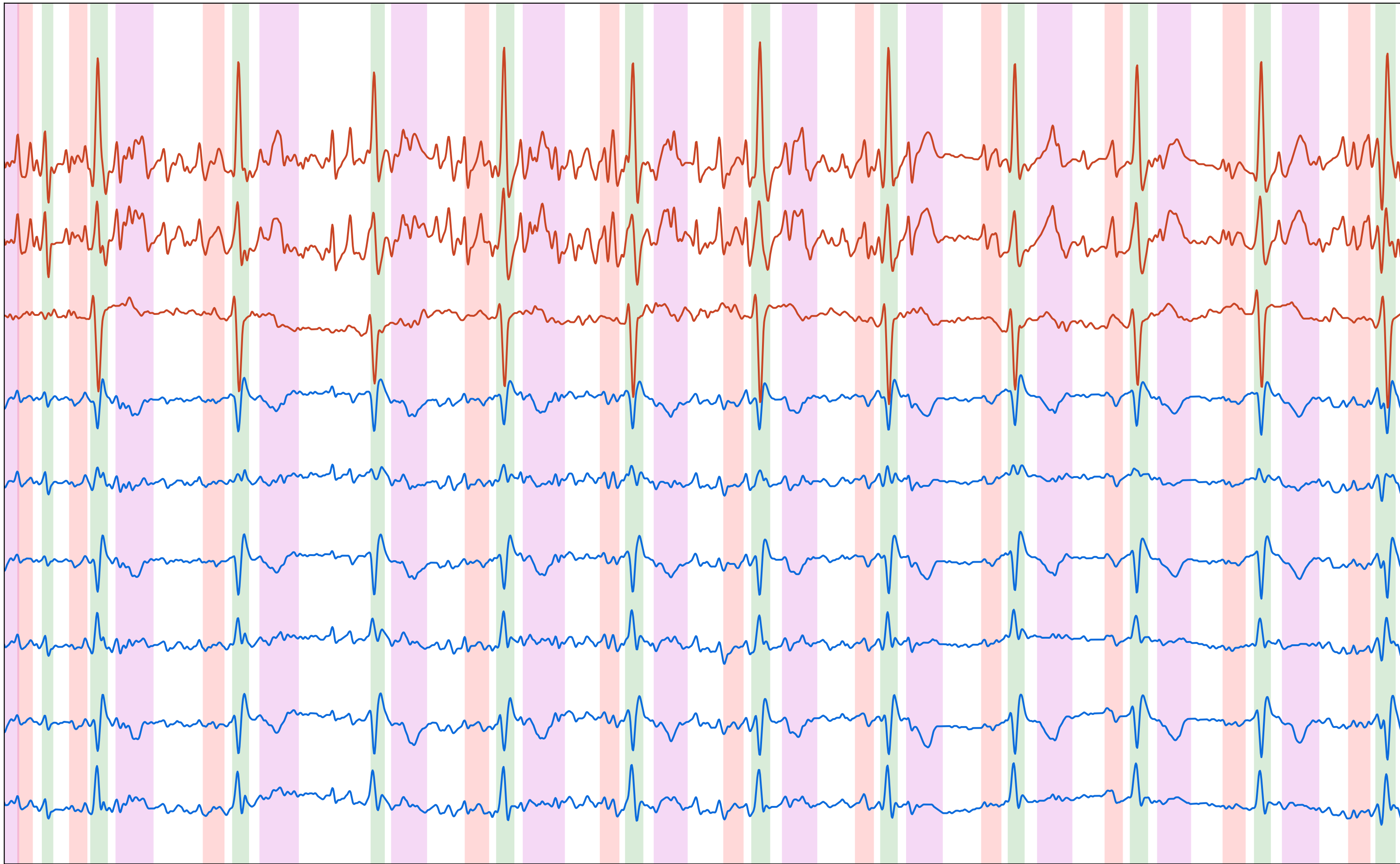

Supplement: Supplementary file 1 [file Datasheet1.zip › brugada4.pdf]

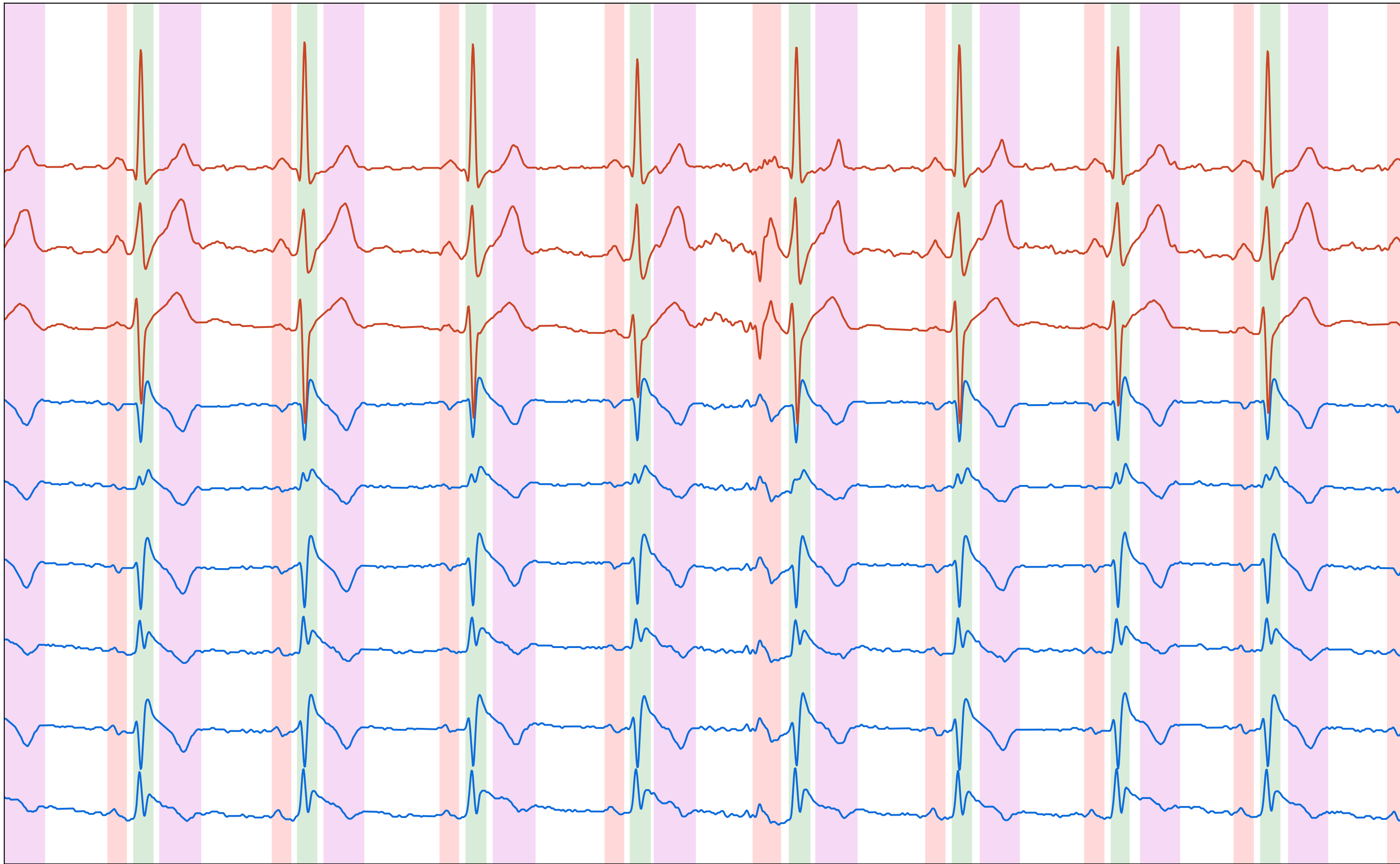

Supplement: Supplementary file 1 [file Datasheet1.zip › brugada5.pdf]

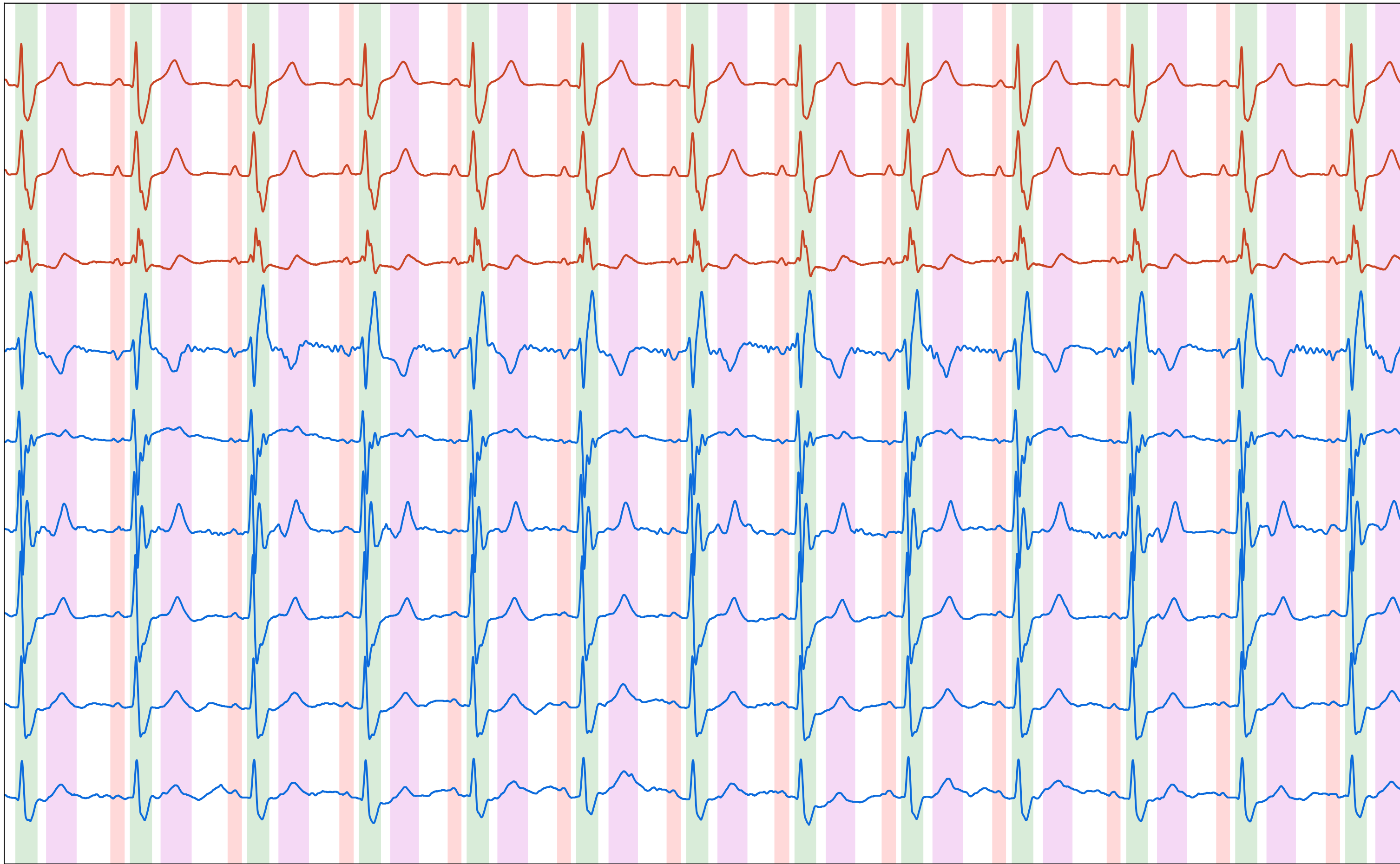

Supplement: Supplementary file 1 [file Datasheet1.zip › fallot1.pdf]

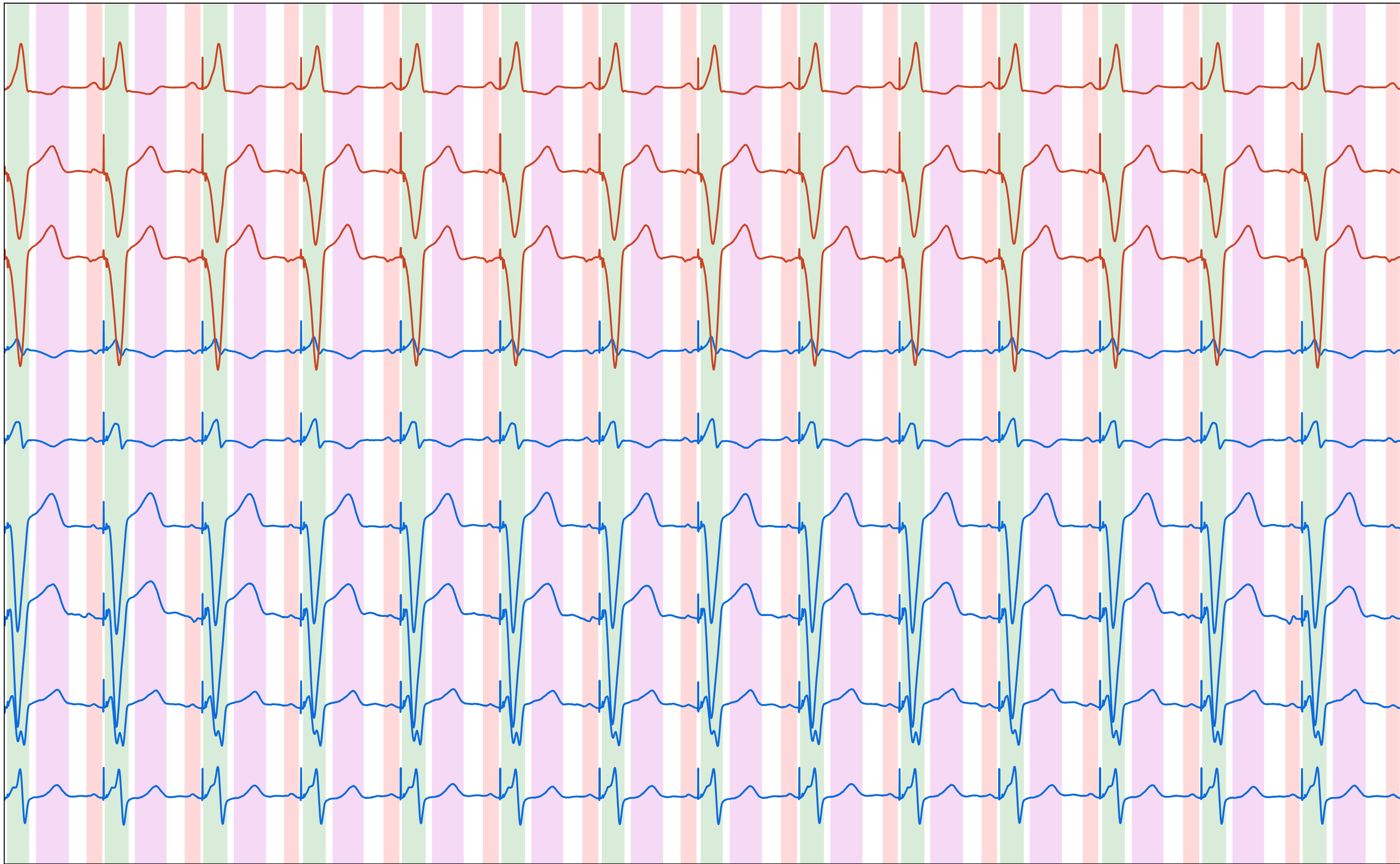

Supplement: Supplementary file 1 [file Datasheet1.zip › fallot2.pdf]

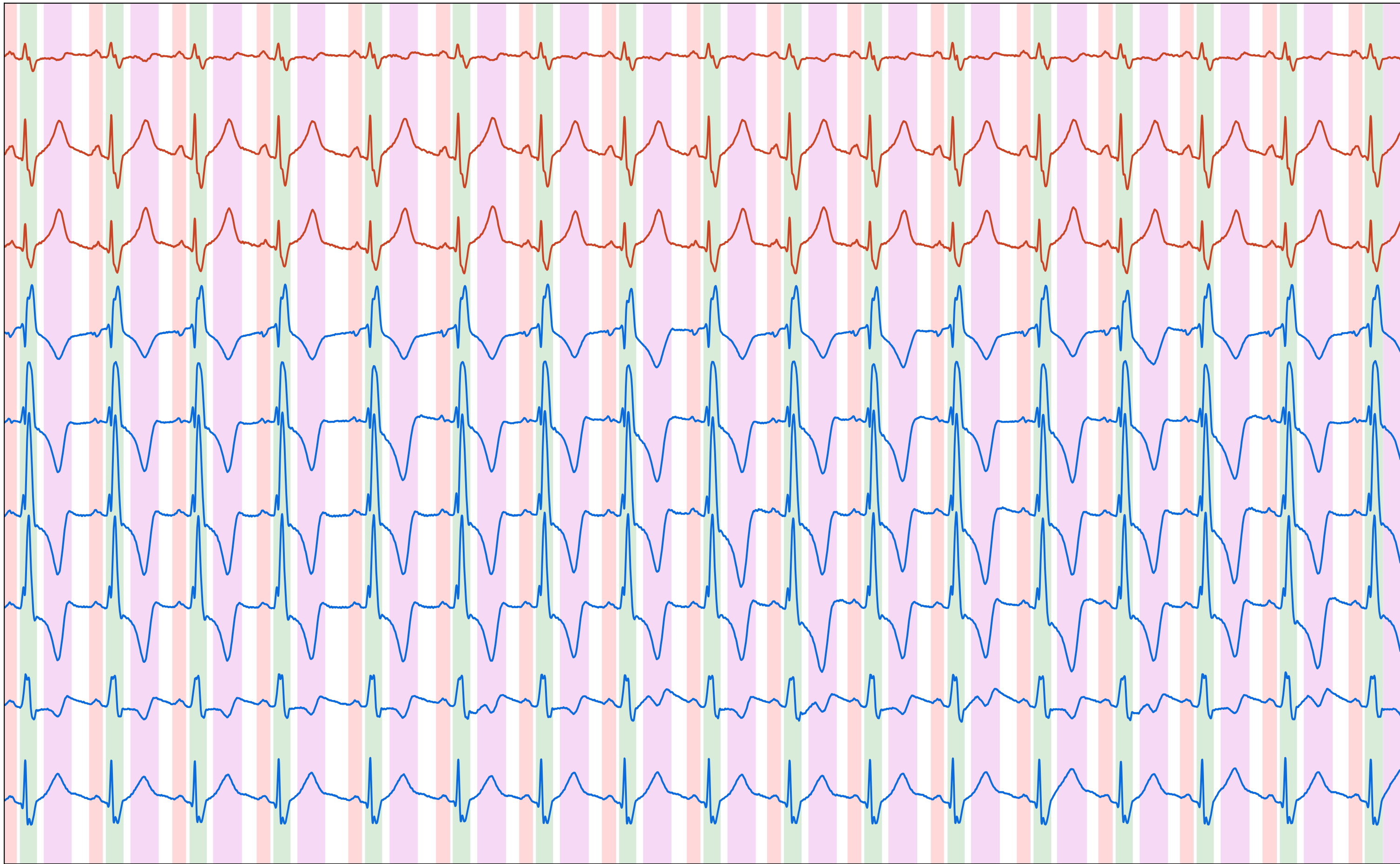

Supplement: Supplementary file 1 [file Datasheet1.zip › fallot3.pdf]

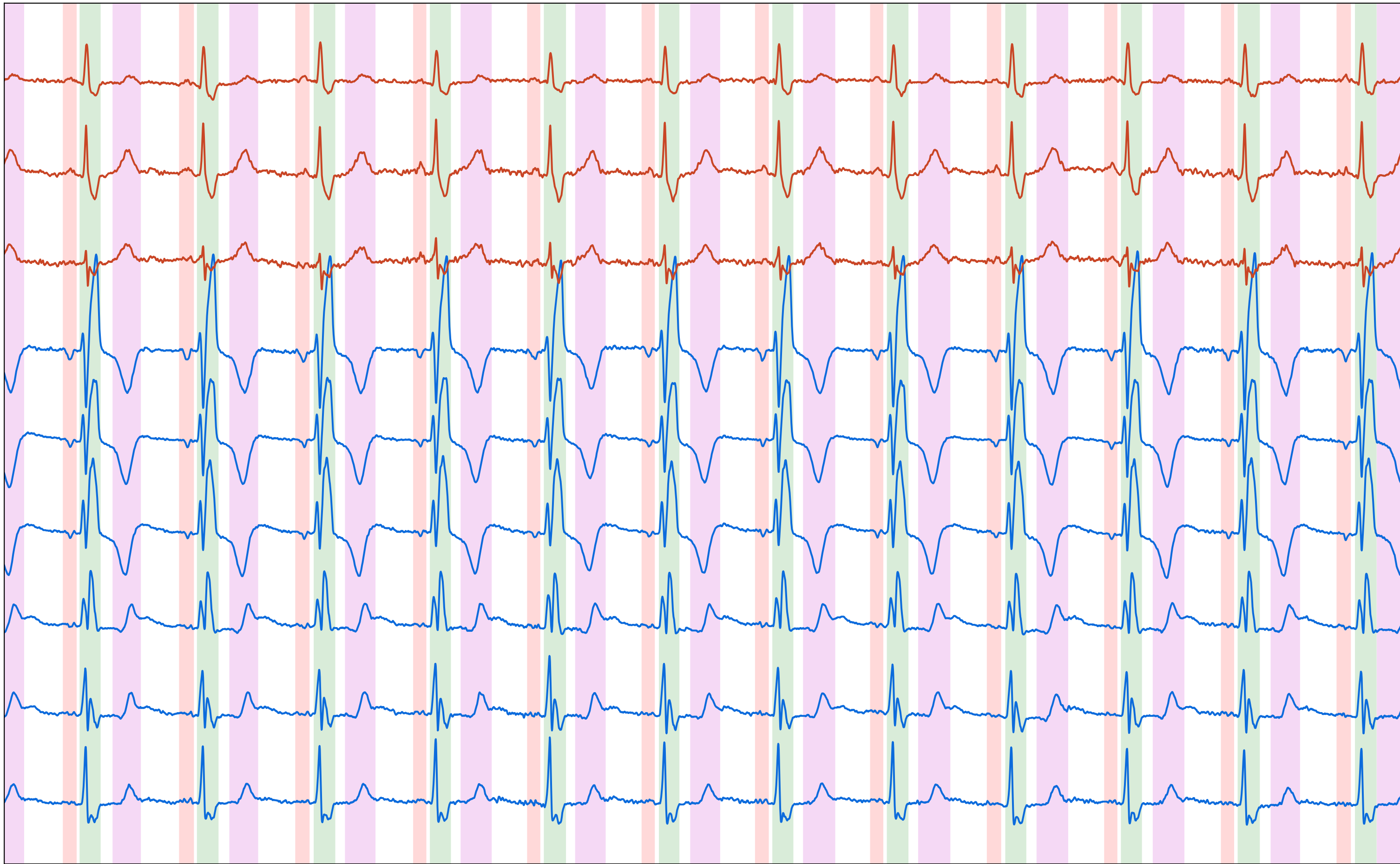

Supplement: Supplementary file 1 [file Datasheet1.zip › fallot4.pdf]

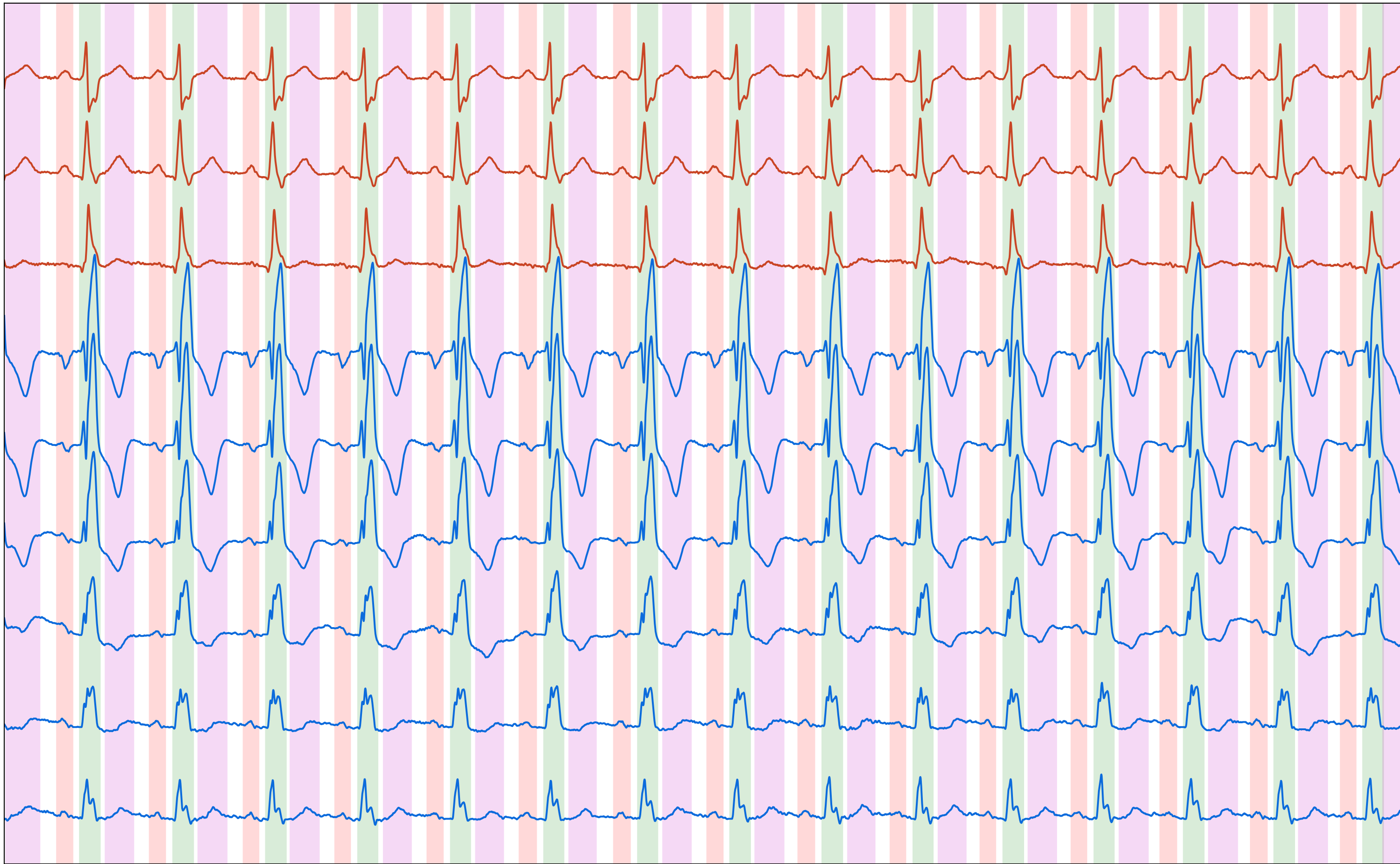

Supplement: Supplementary file 1 [file Datasheet1.zip › fallot5.pdf]

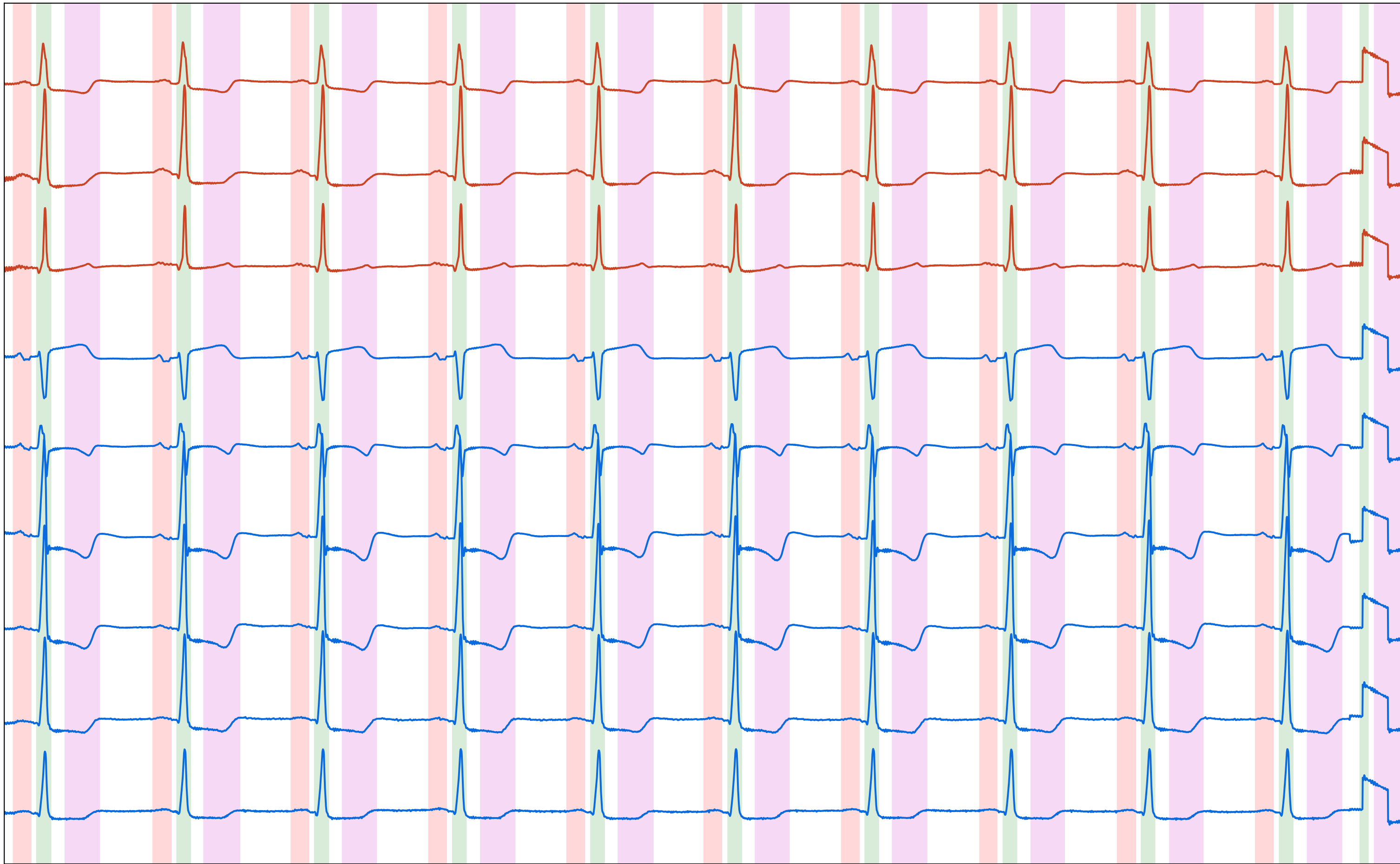

Supplement: Supplementary file 1 [file Datasheet1.zip › hcm1.pdf]

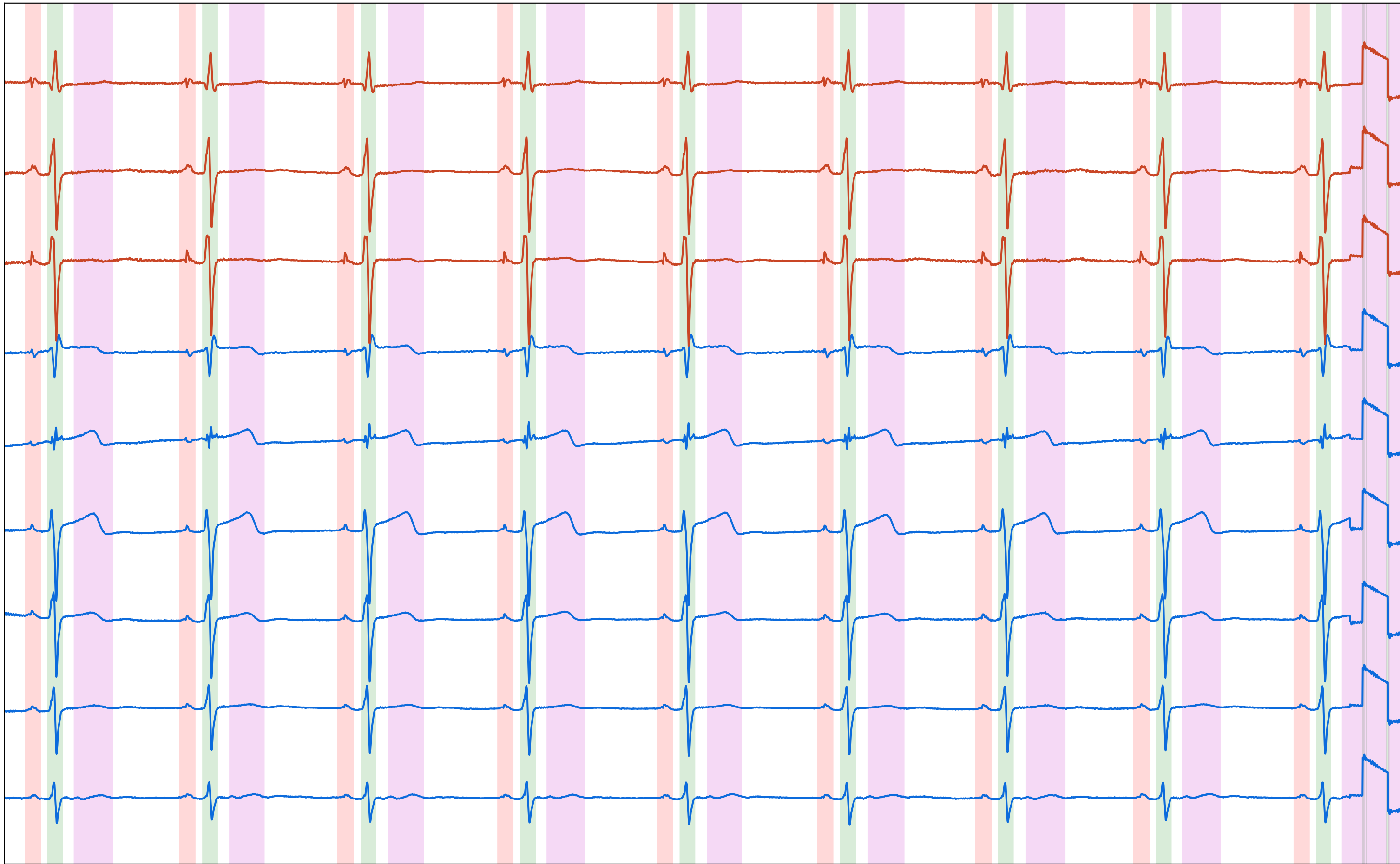

Supplement: Supplementary file 1 [file Datasheet1.zip › hcm2.pdf]

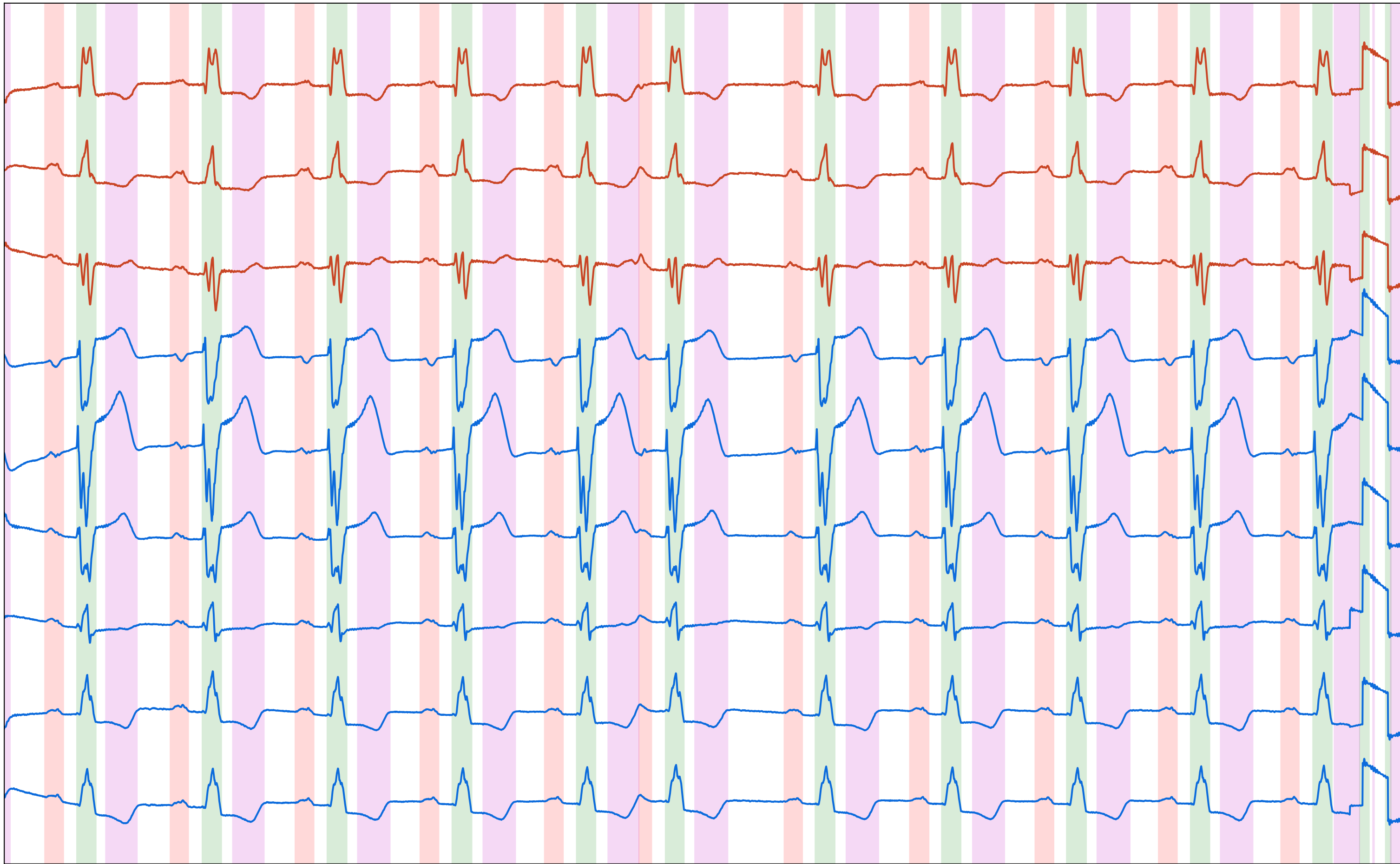

Supplement: Supplementary file 1 [file Datasheet1.zip › hcm3.pdf]

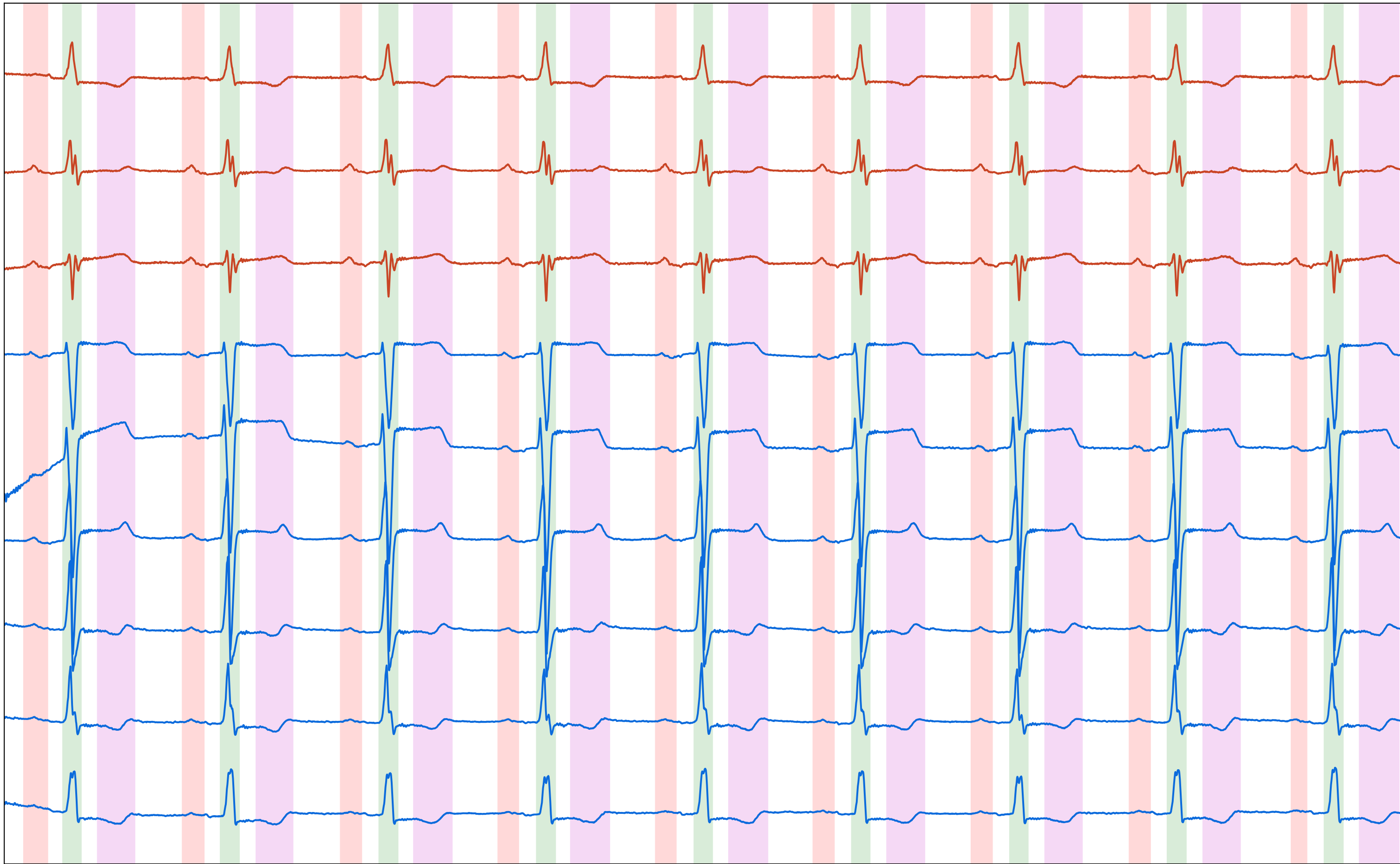

Supplement: Supplementary file 1 [file Datasheet1.zip › hcm4.pdf]

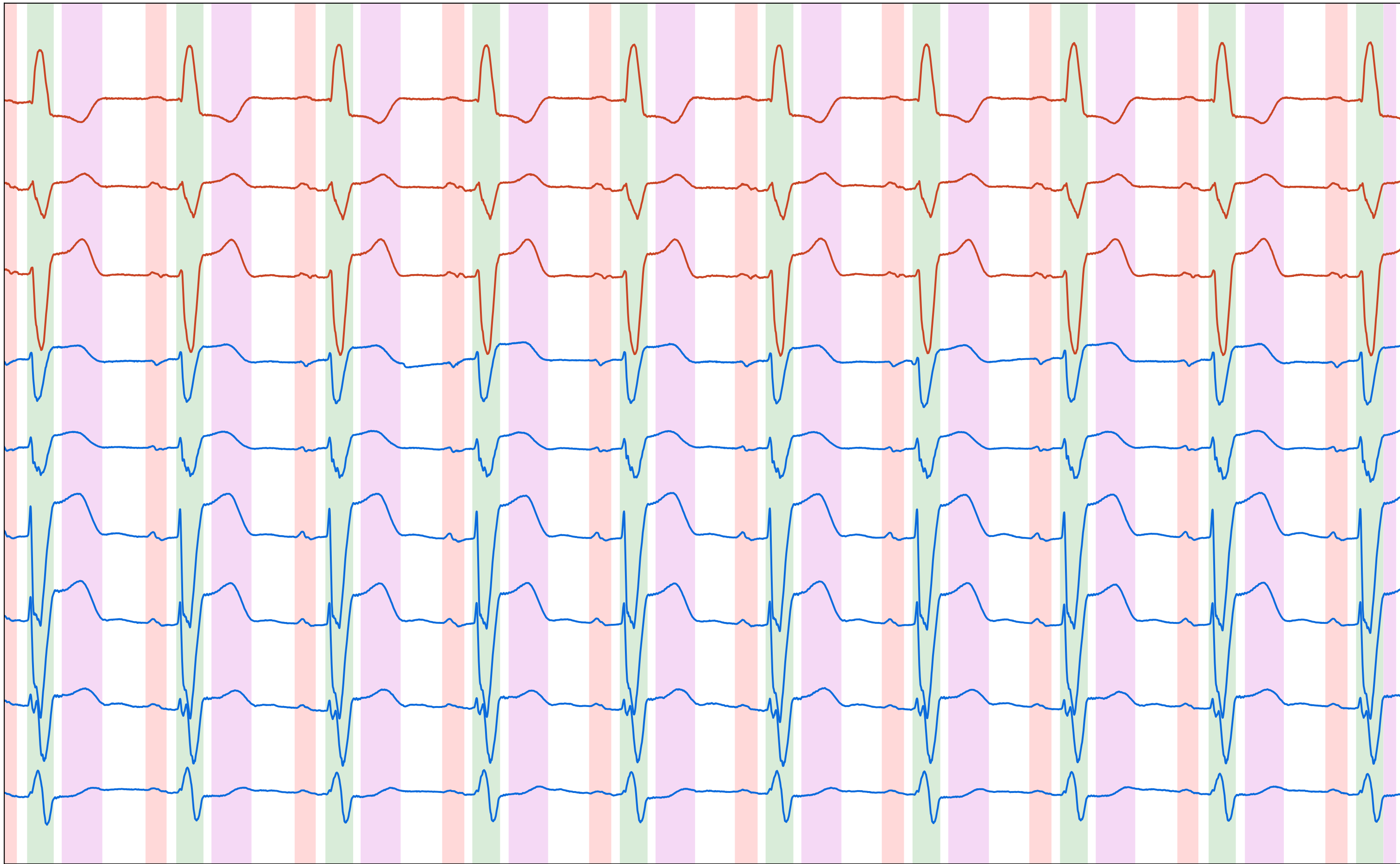

Supplement: Supplementary file 1 [file Datasheet1.zip › hcm5.pdf]

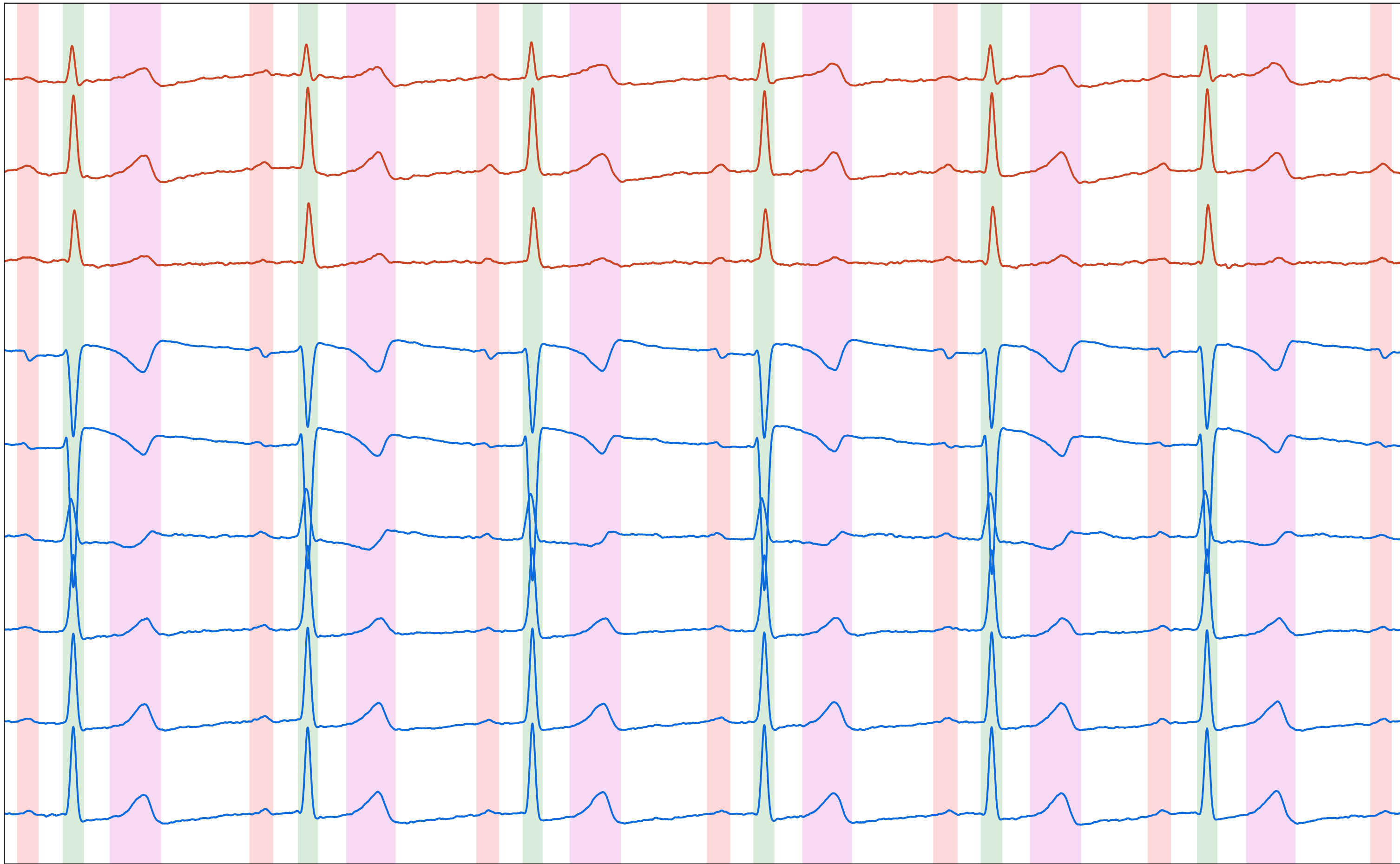

Supplement: Supplementary file 1 [file Datasheet1.zip › iugr_adol1.pdf]

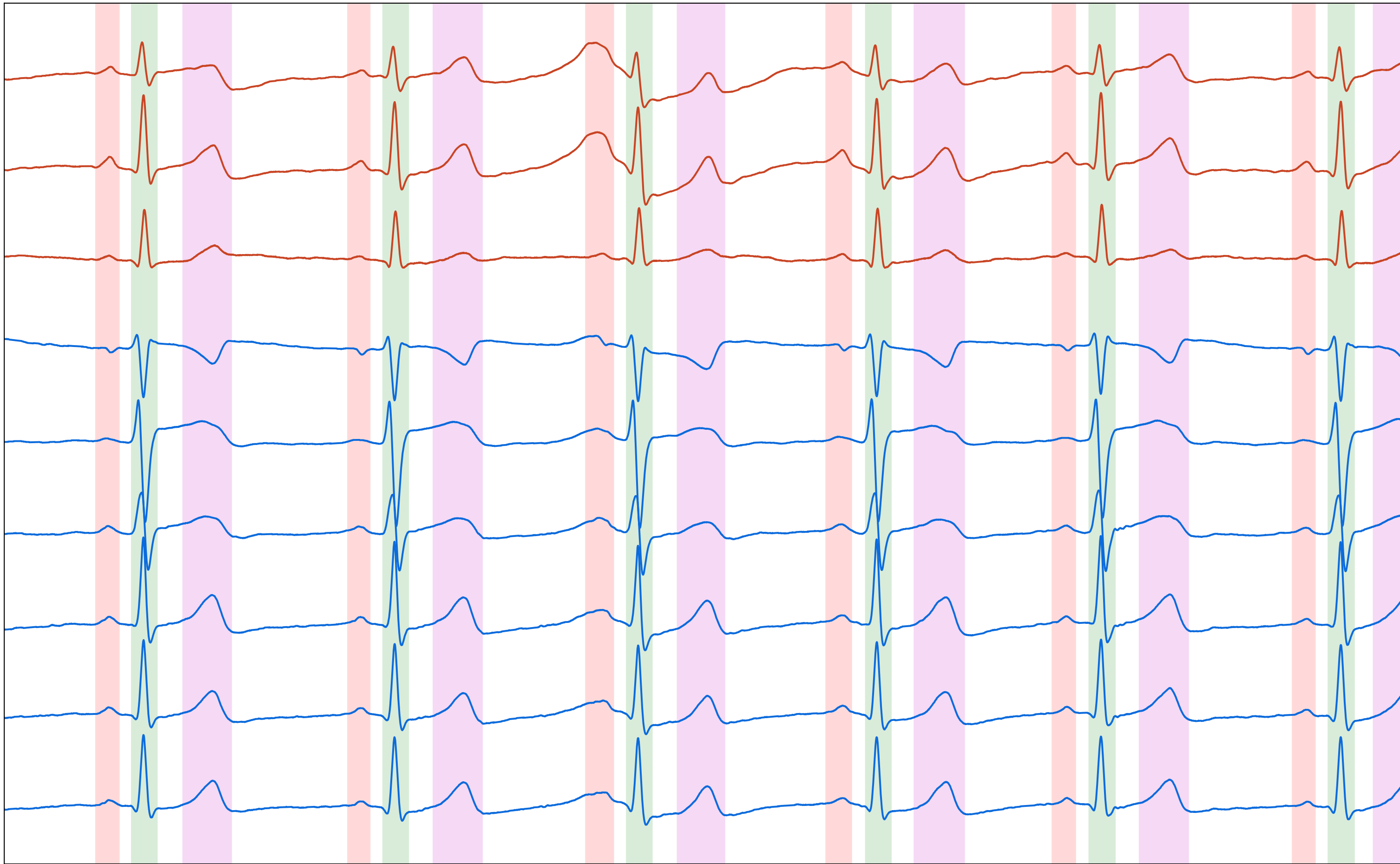

Supplement: Supplementary file 1 [file Datasheet1.zip › iugr_adol2.pdf]

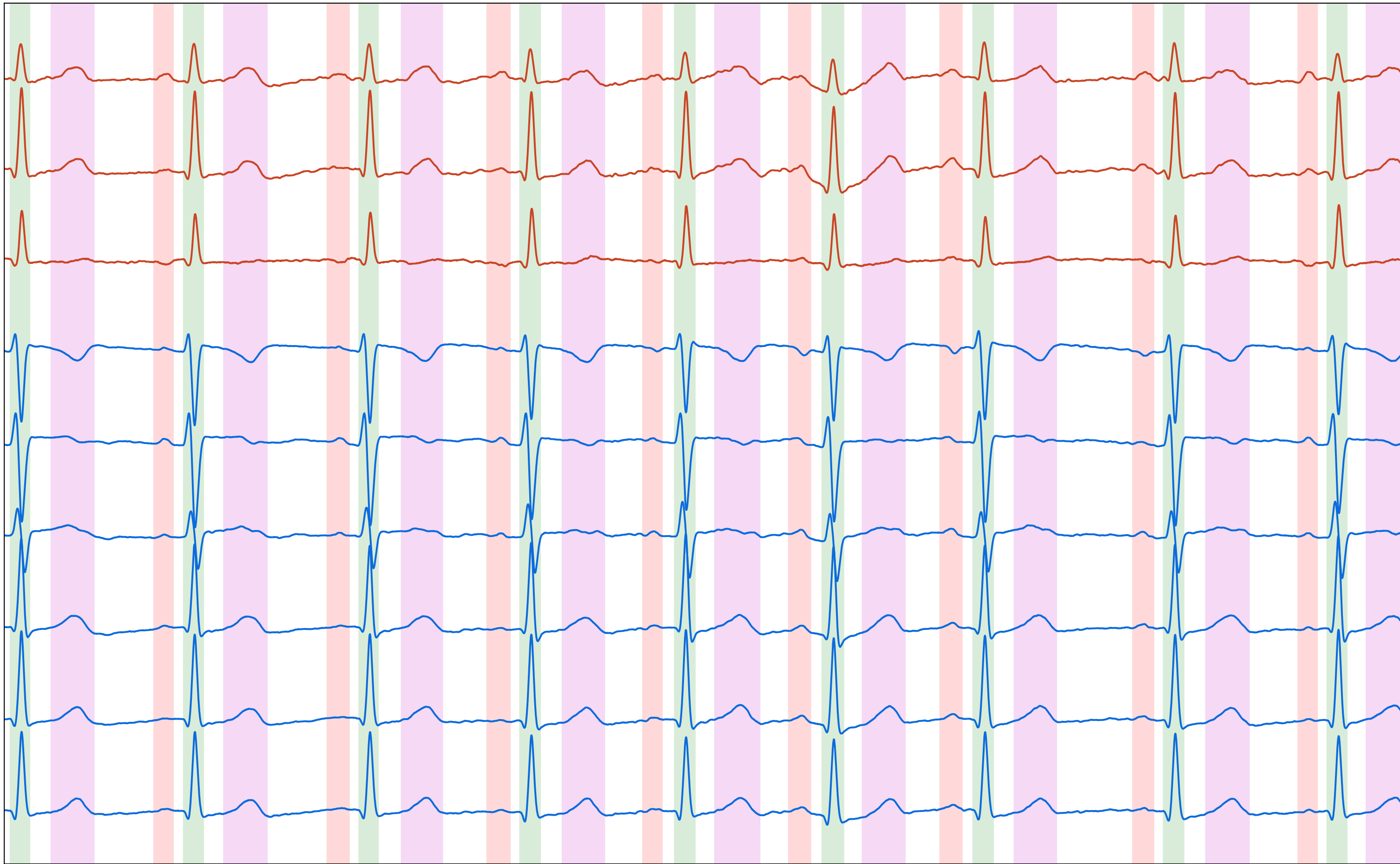

Supplement: Supplementary file 1 [file Datasheet1.zip › iugr_adol3.pdf]

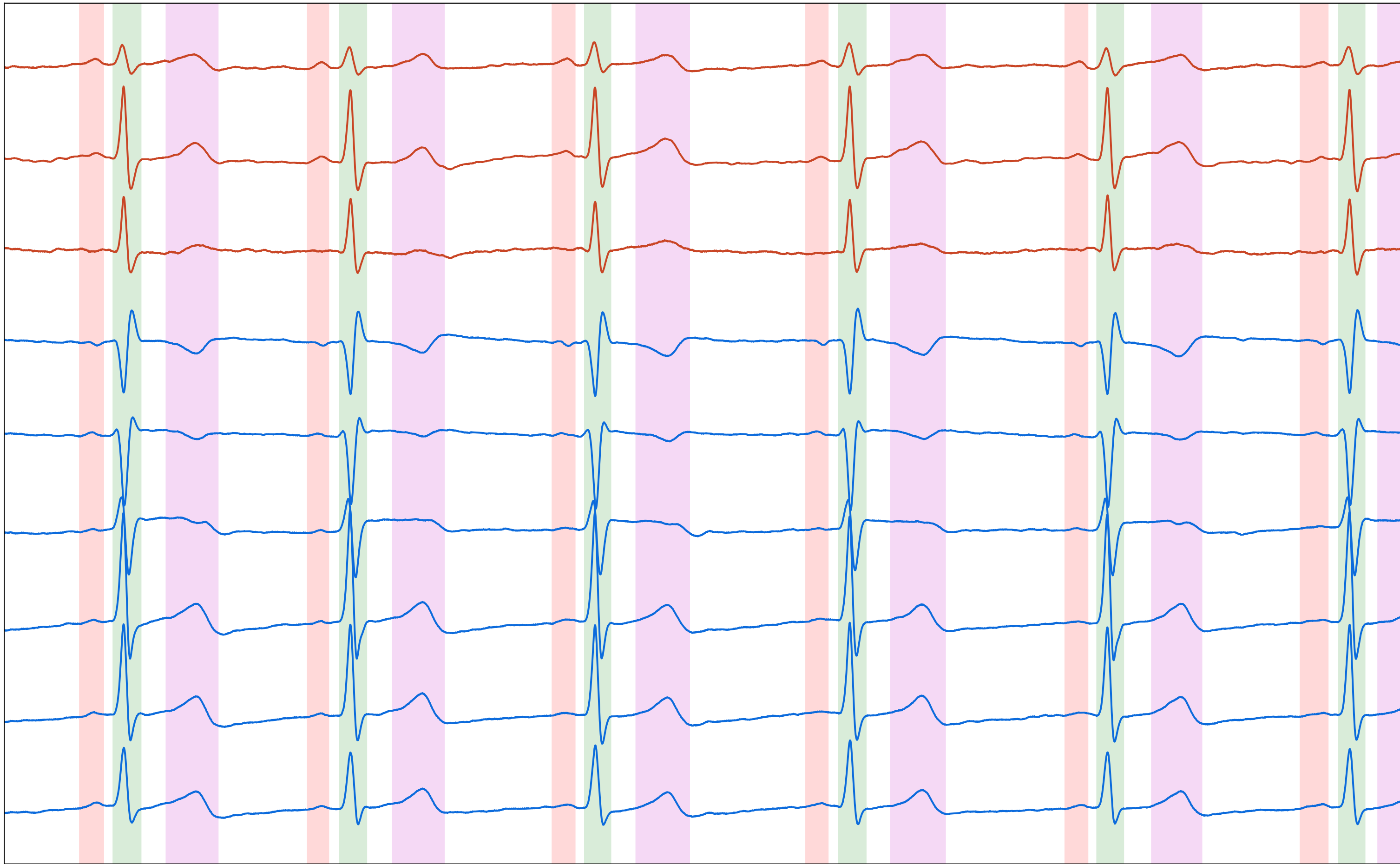

Supplement: Supplementary file 1 [file Datasheet1.zip › iugr_adol4.pdf]

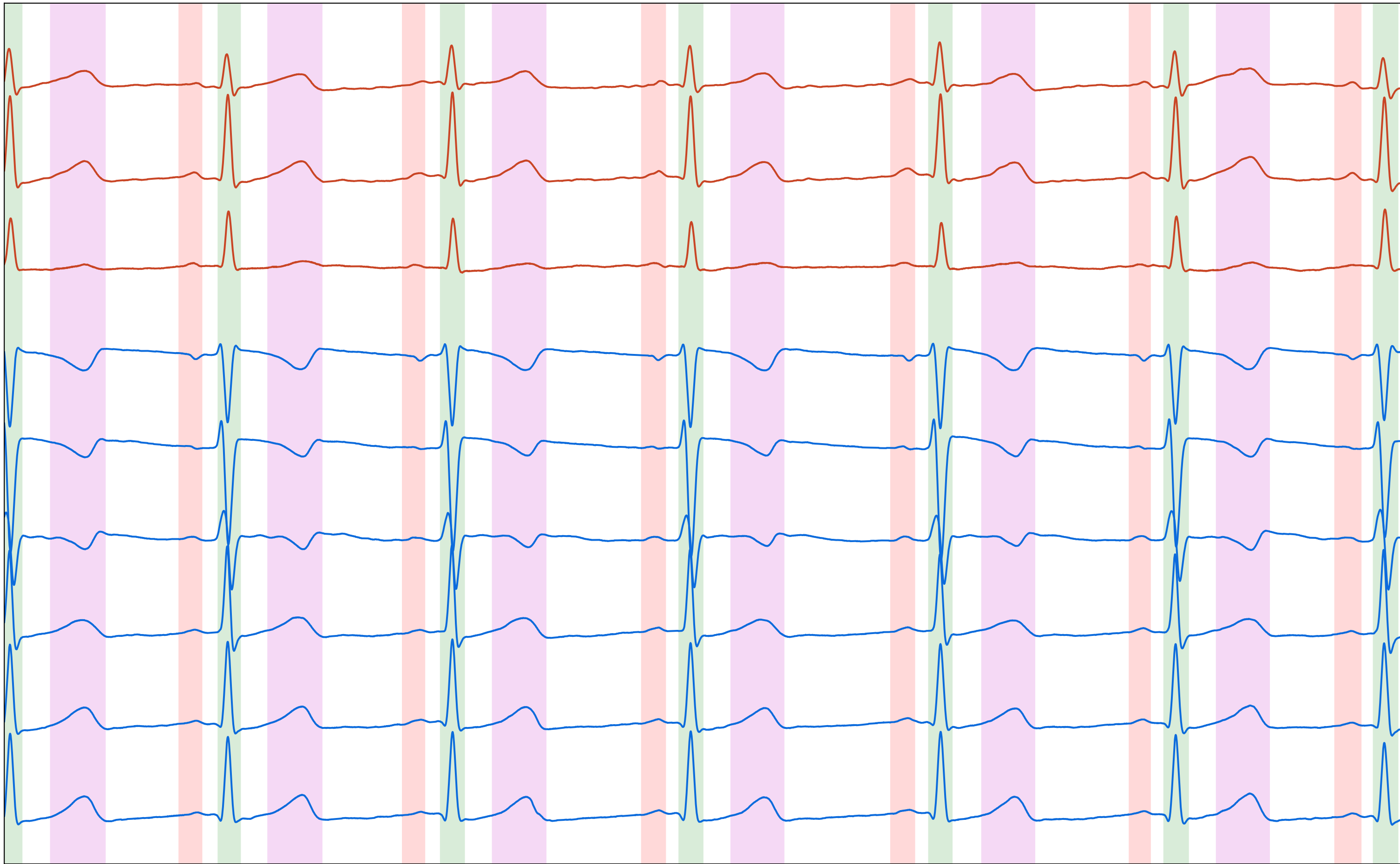

Supplement: Supplementary file 1 [file Datasheet1.zip › iugr_adol5.pdf]

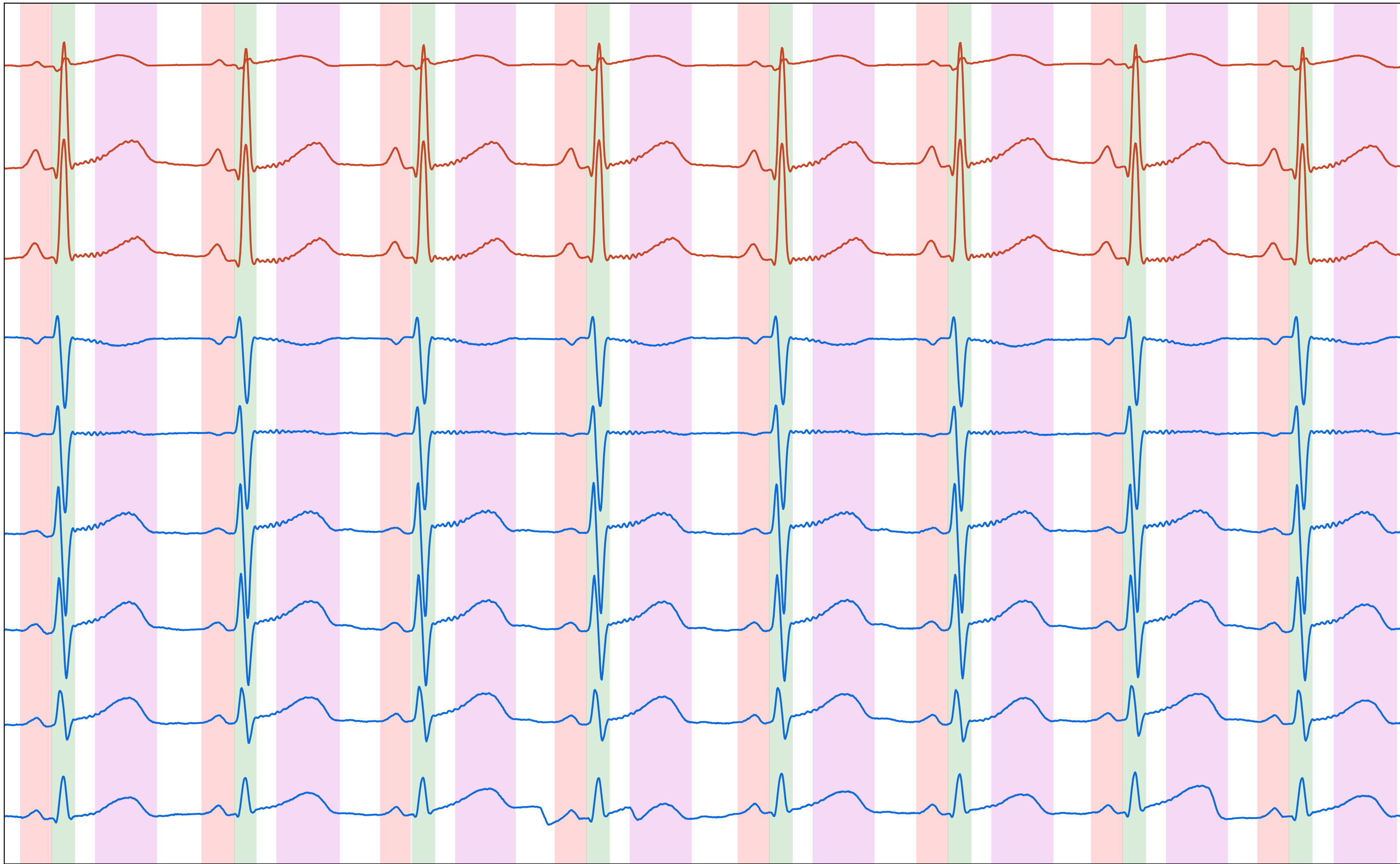

Supplement: Supplementary file 1 [file Datasheet1.zip › longqt1.pdf]

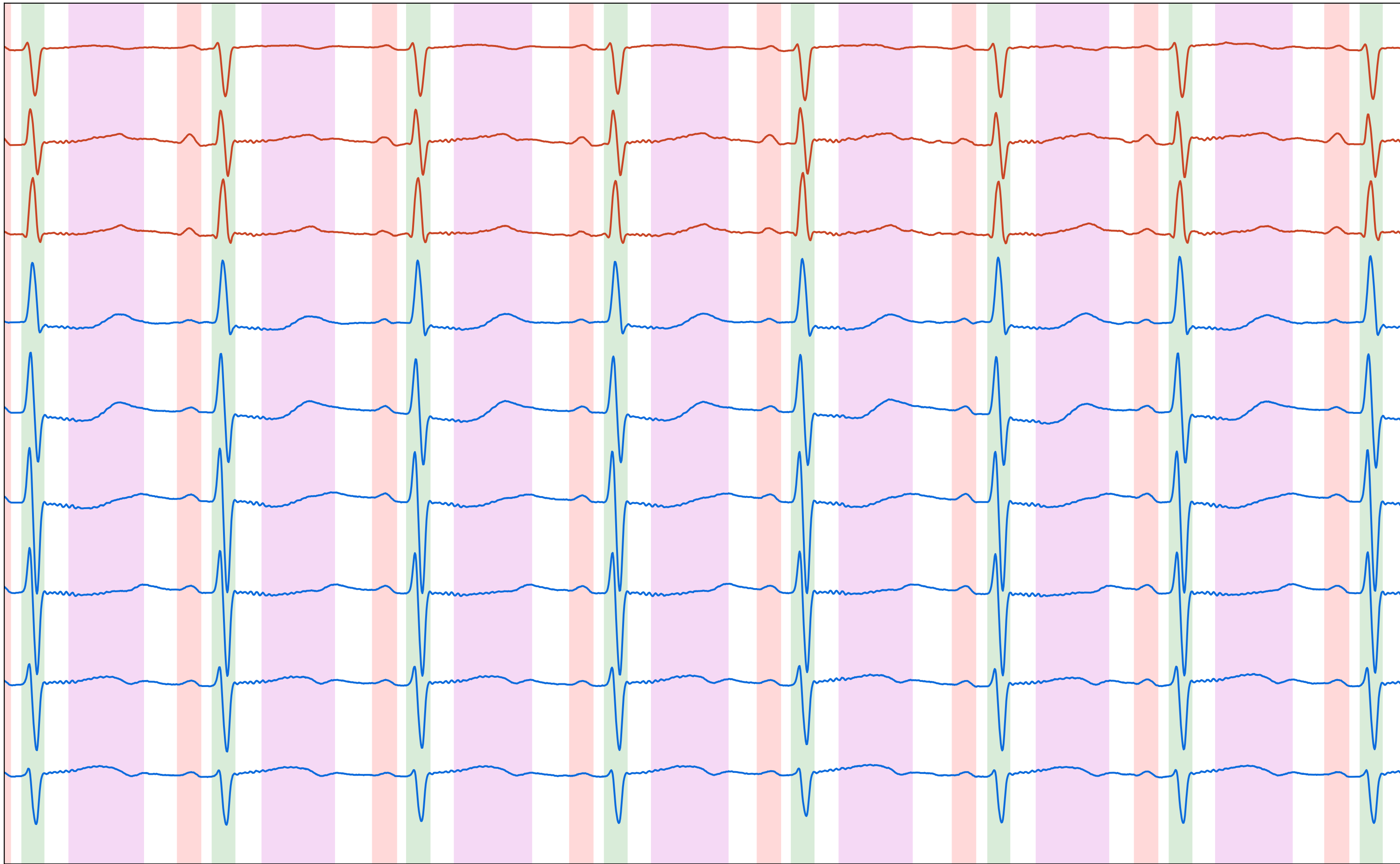

Supplement: Supplementary file 1 [file Datasheet1.zip › longqt2.pdf]

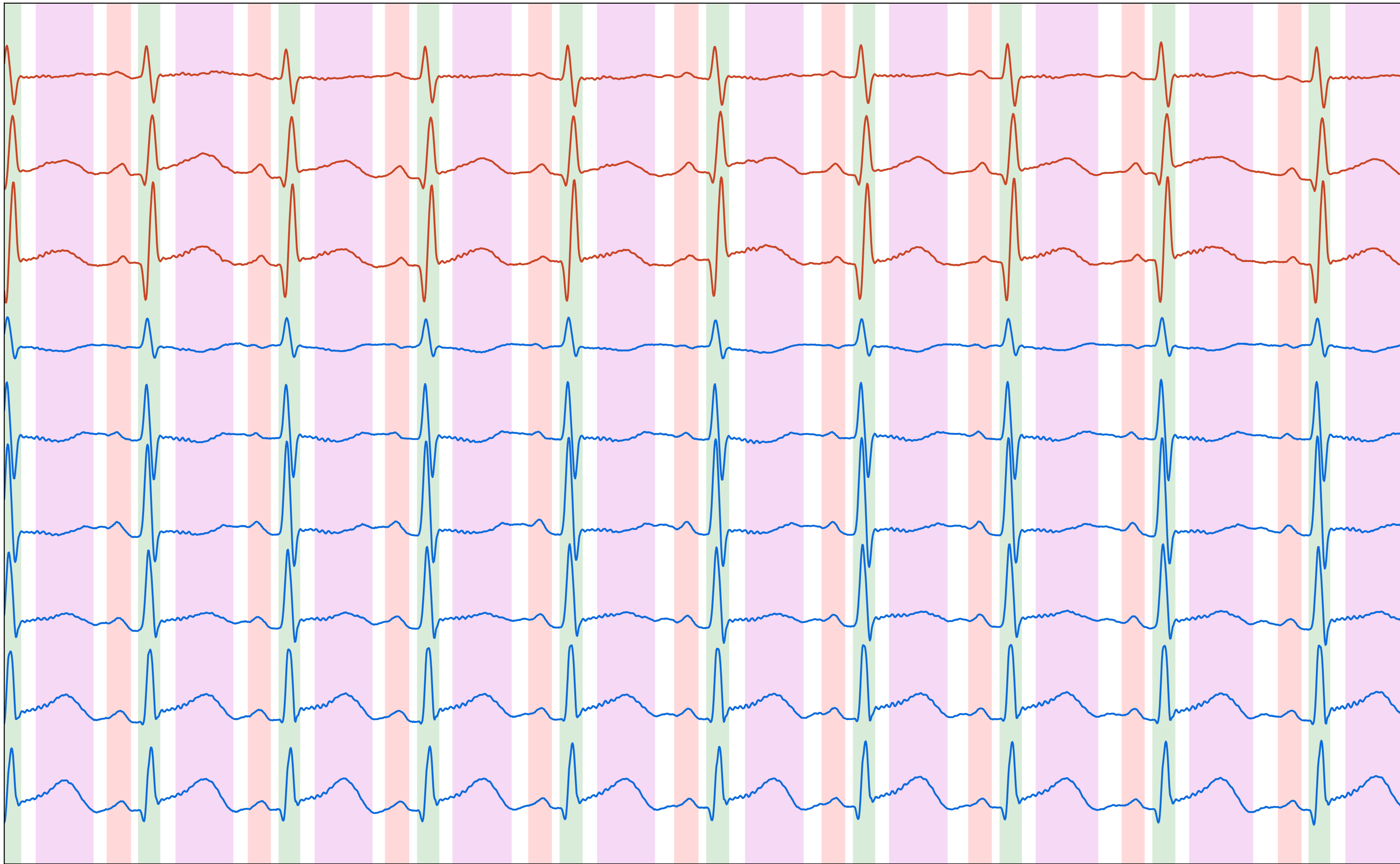

Supplement: Supplementary file 1 [file Datasheet1.zip › longqt3.pdf]

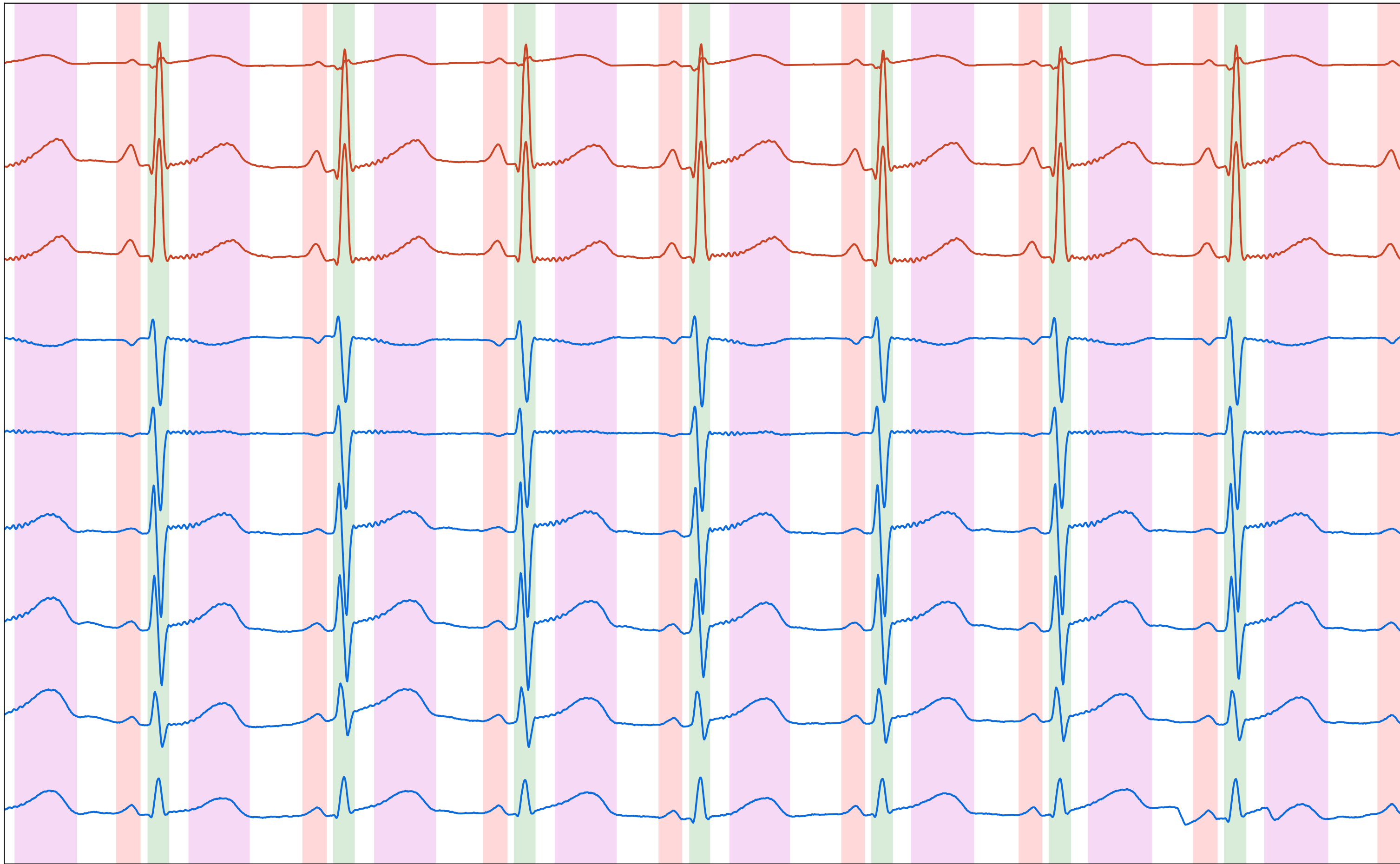

Supplement: Supplementary file 1 [file Datasheet1.zip › longqt4.pdf]
